# Genomic Characterization and Establishment of a Genetic Manipulation System for *Trichoderma* sp. (*Harzianum* Clade) LZ117

**DOI:** 10.3390/jof10100697

**Published:** 2024-10-07

**Authors:** Jie Yang, Cristopher Reyes Loaiciga, Hou-Ru Yue, Ya-Jing Hou, Jun Li, Cheng-Xi Li, Jing Li, Yue Zou, Shuai Zhao, Feng-Li Zhang, Xin-Qing Zhao

**Affiliations:** 1 State Key Laboratory of Microbial Metabolism, Joint International Research Laboratory of Metabolic & Developmental Sciences, School of Life Sciences and Biotechnology, Shanghai Jiao Tong University, Shanghai 200240, China; yangjie1994@sjtu.edu.cn (J.Y.); cris111@sjtu.edu.cn (C.R.L.); yuehouru@sjtu.edu.cn (H.-R.Y.); jing.li@sjtu.edu.cn (J.L.); zhangfengli@sjtu.edu.cn (F.-L.Z.); 2 State Key Laboratory for Conservation and Utilization of Subtropical Agro-Bioresources, Guangxi Research Center for Microbial and Enzyme Engineering Technology, College of Life Science and Technology, Guangxi University, Nanning 530004, China; 2308391012@st.gxu.edu.cn (Y.-J.H.); shuaizhao0227@163.com (S.Z.); 3 Shanghai CHANDO Group Co., Ltd., Shanghai 200233, China; lijun2@chandogroup.com (J.L.); zouyue3@chandogroup.com (Y.Z.); 4 Anhui Key Laboratory of Infection and Immunity, Department of Microbiology, Bengbu Medical University, Bengbu 233000, China; chengx-li@bbmu.edu.cn

**Keywords:** *Trichoderma* sp. LZ117, *T. atrobrunneum*, whole genome sequencing (WGS), genetic manipulation, cellulase production

## Abstract

*Trichoderma* species have been reported as masters in producing cellulolytic enzymes for the biodegradation of lignocellulolytic biomass and biocontrol agents against plant pathogens and pests. In our previous study, a novel *Trichoderma* strain LZ117, which shows potent capability in cellulase production, was isolated. Herein, we conducted multilocus phylogenetic analyses based on DNA barcodes and performed time-scaled phylogenomic analyses using the whole genome sequences of the strain, annotated by integrating transcriptome data. Our results suggest that this strain represents a new species closely related to *T. atrobrunneum* (*Harzianum* clade). Genes encoding carbohydrate-active enzymes (CAZymes), transporters, and secondary metabolites were annotated and predicted secretome in *Trichoderma* sp. LZ117 was also presented. Furthermore, genetic manipulation of this strain was successfully achieved using PEG-mediated protoplast transformation. A putative transporter gene encoding maltose permease (Mal1) was overexpressed, which proved that this transporter does not affect cellulase production. Moreover, overexpressing the native Cre1 homolog in LZ117 demonstrated a more pronounced impact of glucose-caused carbon catabolite repression (CCR), suggesting the importance of Cre1-mediated CCR in cellulase production of *Trichoderma* sp. LZ117. The results of this study will benefit further exploration of the strain LZ117 and related species for their applications in bioproduction.

## 1. Introduction

Species from the genus *Trichoderma* have been widely studied because of their ability to produce cellulolytic enzymes that can degrade lignocellulosic biomass [1,2,3]. Many species of *Trichoderma* spp., including *T. reesei*, *T. viride*, *T. longibrachiatum*, *T. koningii*, and *T. harzianum*, are ubiquitous colonizers of cellulolytic materials. Various variants or new species of *Trichoderma* sp. appearing with superior cellulase production and secretion are screened or isolated for further studies on the mechanism of cellulose degradation. In addition, *Trichoderma* species are also famous for their biocontrol capabilities in agriculture. Among these species, *T. harzianum* strains, famous for their biocontrol application, were also reported to have great potentials in cellulase production, such as *T. harzianum* EM0925 [4], *T. harzianum* EUA20 [5], *T. harzianum* TRIC03 [6], *T. harzianum* KUC1716 [7], and *T. harzianum* LZ117 [8].

*T. harzianum* was previously recognized as an “aggregate species”, potentially including two or more morphologically cryptic but biologically distinct species [9]. Since the internal transcribed spacer rDNA regions (ITS) are limited in the delimitation of *Trichoderma*, DNA barcodes, such as the second largest nuclear RNA polymerase subunit encoding gene (*rpb2*), and translation elongation factor 1-alpha encoding gene (*tef1*), have been introduced to assist in species identification [9]. To revise the taxonomy of *T. harzianum* species in sensu stricto, the taxonomy of the *T. harzianum* species complex has been revised to include at least 14 species, including *T. guizhouense*, *T. harzianum*, *T. inhamatum*, *T. lentiforme*, *T. lixii*, *T. afarasin*, *T. afroharzianum*, *T. atrobrunneum*, *T. camerunense*, *T. endophyticum*, *T. neotropicale*, *T. pyramidale*, *T. rifaii*, and *T. simmonsii* [9]. *T. harzianum* ITEM908 was reclassified as *T. atrobrunneum* via ITS-TEF1 analysis [10], indicating the importance of polyphasic taxonomy. Multilocus phylogenetic studies of *T. harzianum* have shown the phylogenetic diversity of morphological species [11]. 

The bioconversion of lignocellulosic biomass benefits sustainable development, and it is highly desired to develop robust cellulase producers. In our previous studies, *T. harzianum* LZ117 was proven to be a promising cellulase producer for the bioconversion of lignocellulosic biomass [8,12]. LZ117 is remarkable in its dramatically earlier enzyme induction (48 h) and shorter fermentation time (4–5 days) in cellulase production, compared with the widely studied mutant strain *T. reesei* QM9414 [8]. Genome-scale studies of *Trichoderma* strains are significant for exploring their bioproduction potential. However, so far, studies on the genome sequences of *Trichoderma* strains have mainly focused on biocontrol potential and the biosynthesis of active natural products [13,14,15,16,17,18,19]. Genomic studies on cellulase-producing *Trichoderma* species remain limited [20,21,22], and phylogenomic studies of *Trichoderma* sp. LZ117 have not been reported.

To further characterize the LZ117 strain and explore its biosynthesis potential, herein, we performed multilocus phylogenetic analysis and whole genome sequencing (WGS) analyses [23,24]. As a result, LZ117 was re-identified as a new species close to *T. atrobrunneum* (*Harzianum* clade) and was renamed *Trichoderma* sp. LZ117. Then, we analyzed predicted proteins, focusing on those associated with biomass degradation, such as carbohydrate-active enzymes (CAZymes) and transporters. In addition, to develop this strain as a new platform producer for biotechnological purposes, we also established a genetic manipulation system via PEG-mediated protoplast transformation. The results in this work suggest novel aspects of natural *Trichoderma* isolates and provide a basis for the further application of natural *Trichoderma* strains in the bioconversion of lignocellulosic biomass.

## 2. Materials and Methods

### 2.1. Genome Sequencing, Assembly, and Assessing

*Trichoderma* sp. (formerly *T. harzianum*) LZ117 was initially isolated from Tibet, China [12], and the strain is deposited in China General Microbiological Culture Collection Center (CGMCC) with the accession number of CGMCC17184. 

A monoconidial culture of LZ117 was grown on MEA (Malt Extract Agar, Sangon Biotech, Shanghai, China) for 5–7 days [8]. A fungal mycelium was collected and ground to a fine powder via liquid nitrogen. DNA extraction was performed as described previously [25]. The genome sequencing was subjected to an integration of the Nanopore PromethION platform (for the library of 1D mate-paired reads) and the Illumina NovaSeq PE150 platform (for the library of 350 bp mate-paired reads) for library preparation workflow. The genome assembly was performed by Unicycler, available at https://github.com/rrwick/Unicycler (accessed on 22 June 2020).

An integrity assessment of the assembled LZ117 genome was evaluated using the fungi_odb10 database within the BUSCO program package [26].

### 2.2. Phylogenetic Analysis

Species assignment of LZ117 was achieved by applying the genealogical concordance phylogenetic species recognition concept based on the gene sequences of the RNA-polymerase II encoding gene (*rpb2*) and translation elongation factor 1-alpha encoding gene (*tef1*). 

To construct the phylogenetic tree, *rpb2* datasets (Supplementary Materials S1) and *tef1* datasets (Supplementary Materials S2) were retrieved from Genbank with 47 *Trichoderma* strains, including the species *T. guizhouense*, *T. harzianum*, *T. lentiforme*, *T. afarasin*, *T. afrohazianum*, *T. atrobrunneum*, *T. camerunense*, *T. endophyticum*, *T. rifaii*, *T. simmonsii*, *T. lixii*, *T. neotropicale*, and *T. intamatum*. Maximum likelihood (ML) phylogenetic analyses were performed to determine the evolutionary pattern using MEGA7 (Molecular Evolutionary Genetics Analysis software, Version 7.0.21) [27], and further polishing was conducted via the online Interactive Tree Of Life (iTOL) platform [28].

### 2.3. Generation of a Time-Scaled Phylogeny of Trichoderma Species

Species genomes and proteome files were retrieved from the NCBI database (http://www.ncbi.nlm.nih.gov/) (assessed on 26 September 2022). The species involved in the time-scaled phylogeny included *Trichoderma* sp. LZ117 (SAMN41090611), *T. harzianum* CBS 226.95 (SAMN00769983), *T. atrobrunneum* ITEM 908 (SAMN08325511), *T. guizhouense* (SAMN04535176), *T. afroharzianum* (SAMN22210987), *T. virens* Gv29-8 (SAMN17838940), *T. citrinoviride* (SAMN05369575), *T*. *longibrachiatum* ATCC 18648 (SAMN00767620), *T. koningii* (SAMD00028335), *T. reesei* QM6a (SAMN05250858), *T. brevicompactum* (SAMN06320626), *T. asperellum* (SAMN00769595), *T. erinaceum* (SAMN14917834), *T. atroviride* (SAMN02744066), *T. gamsii* (SAMN02849381), *T. koningiopsis* (SAMD00028335), *T. hamatum* (SAMN02981472), *T. koningiopsis* POS7 (SAMN06106985), *T. parareesei* (SAMN03784587), *Claviceps purpurea* (SAMEA2272775), and *Neurospora crassa* (SAMN02953583).

For the species with no available proteome data, including *T. atrobrunneum*, *T. erinaceum*, *T. brevicompactum*, *T. koningii*, and *T. koningipsis*, an additional annotation was conducted to extract coding regions of their genomes. We built a blast database with each genome of the five species. The protein sequences of the remaining *Trichoderma* species were used as queries in the alignment step using TBlastN. Only the best high-scoring pair with a match higher than 80% was kept. Sequences without a canonical starting codon were removed. 

ProteinOrtho5 from Galaxy Europe version 6.0.32 (www.bioinf.uni-leipzig.de (assessed on 26 September 2022)) was used to identify orthologous sequences into groups. This step produced 19,287 groups, but only groups with at least one line for each species were kept. If one species was represented by more than one sequence, only the first one was held for further analysis. Five hundred seventy-six groups remained. For each of the groups, MAFFT was used with auto-settings to produce a series of multiple alignments that were then passed through Gblocks v0.19b in Linux to remove all the regions that were not conserved. The sequences were concatenated for a final string of 208,364 amino acid positions for each species.

The aligned strings were used as input for the Beast2 CladeAge app [29]. The following time calibration points were provided [24]: a common ancestor for the Clavicipitaceae crown group for a 95% range of 114–120 Mya [30] and a common ancestor Hypocreales calibrated for a 95% range of 190–196 Mya [31]. Species in these clades were forced into monophyletic groups. The MCMC analyses were performed using a chain of length 20,000,000 sampling every 1000 generations in Beast v2.6.6, and 25% of the trees were discarded as burned up as estimated by Tracer.

### 2.4. Gene Prediction and Functional Annotation

Gene prediction of *Trichoderma* sp. LZ117 was implemented with the assistance of previously available transcriptome [12] (Accession: PRJNA613881) and whole genome sequencing (WGS, Accession: PRJNA1105021, https://dataview.ncbi.nlm.nih.gov/object/PRJNA1105021?reviewer=rjj2iol6bp232arov7o0dd55rl (assessed on 26 September 2022)) via the Augustus and Funannotate pipeline [32]. The predicted proteins were then annotated by Gene Ontology (GO), Eukaryotic Orthologous Groups (KOGs), Kyoto Encyclopedia of Genes and Genomes (KEGG), and Pfam. The protein sequences of the predicted genes were compared with each functional database by Diamond (evalue ≤ 1 × 10^−5^). For the comparison result of each line, the highest score (default identity ≥ 40%, coverage ≥ 40%) of the comparison result was selected for comment.

The secreted proteins of *Trichoderma* sp. LZ117 were predicted via SignalP (Version 4.1) [33] and TMHMM (Version 2.0c) [34]. When signal peptides and transmembrane structures were detected, protein sequences were considered as secreted proteins.

The gene expression profile of the CAZymes (carbohydrate-active enzymes) was annotated via dbCAN2 meta serve (https://bcb.unl.edu/dbCAN2/ (assessed on 26 September 2022)) with the following tools: HMMER, eCAMI, and DIAMOND. The selected data were analyzed using at least two tools.

### 2.5. Vector Construction and Protoplast Transformation

To overexpress targeted genes under the control of the inducible promoter, the promoter P*pdc* in the pCZF3 plasmid [35] was replaced by P*chb1* via N*co* I and P*ac* I sites. The total length of transcription factor Cre1 (648 bp) in *Trichoderma* sp. LZ117, which was amplified from LZ117 genomic DNA via LZ117-OECre1-F and LZ117-OECre1-R (Supplementary Materials S3, Table S1), was then fused into the redesigned pCQS plasmid (11,222 bp) via X*ba* I and N*co* I sites to obtain the plasmid pCQS-OECre1 (11,770 bp) (Supplementary Materials S3, Figure S1). 

The overexpression plasmid pCQS-OEMal1 (12,865 bp) for transporter Mal1 (1643 bp) was similarly constructed. The overexpression cassettes were amplified via PgpdA-F and TtrpC-R (Supplementary Materials S3, Table S1). The overexpression cassettes were then transformed into the protoplasts of LZ117 via PEG-mediated transformation, as previously described by Gruber et al. (1990) with modification [36]. Transformants with hygromycin-resistant transformants were selected on TB3 medium (yeast extract 3 g/L, casamino acid 3 g/L, sucrose 200 g/L, and agar 15 g/L) with hygromycin B (200 μg/mL). The transformants were then transferred to an MEA medium for sporulation after verification via PCR.

### 2.6. Quantitative Reverse Transcription Polymerase Chain Reaction Analysis

The methods used for RNA extraction and RT-qPCR (real-time reverse transcription polymerase chain reaction) were described by Meng et al. 2020 [37]. In brief, mycelia were cultured and harvested at 48 h, and total RNA was extracted using the HiPure Yeast/Bacterial RNA Kit (Magen, Guangzhou, China), and cDNA (complementary DNA) synthesis was carried out using the GoldenstarTM RT6 cDNA Synthesis Kit Ver2 (Tsingke, China). RT-qPCR analysis was carried out with 2×T5 Fast qPCR Mix (SYBR Green I) (Tsingke, Beijing, China) using the primers listed in Supplementary Materials S3, Table S1. The relative transcription of the *cre1* gene was normalized according to the 2^−ΔΔCt^ method [38].

### 2.7. Assays for Cellulase and Mycelial Biomass

Cellulase production in shake flasks was performed using previously described methods with cellulose Avicel as the inducer [8]. Filter paper activity (FPase) was measured as described in [39]. In brief, the diluted crude enzyme (0.5 mL) was reacted with a rolled Whatman No.1 filter paper strip in a colorimetric cylinder filled with 1.5 mL of 50 mM citrate buffer (pH 4.8) for one hour in a 50 °C water bath. DNS (3,5-dinitrosalicylic acid) was then added to stop the reaction. The absorbance of the reaction solution samples (200 μL in a 96-well plate) was measured at 540 nm via a spectrophotometer after boiling the DNS mixture and diluting the reaction solution to 25 mL with deionized water. All experiments were performed in at least three biological replicates with *p* < 0.05, and representative data were shown.

Since Avicel was used as the substrate for cellulase production. The mycelial biomass was characterized indirectly by measuring intracellular proteins extracted via 1 M NaOH following the protocol previously described in [40]. After the samples were incubated for 24 h at room temperature for NaOH extraction, the supernatant protein samples were assayed using the BCA Kit (Vazyme, Nanjing, China).

## 3. Results

### 3.1. Taxonomic Assignment of Trichoderma sp. LZ117

**Figure 1:**
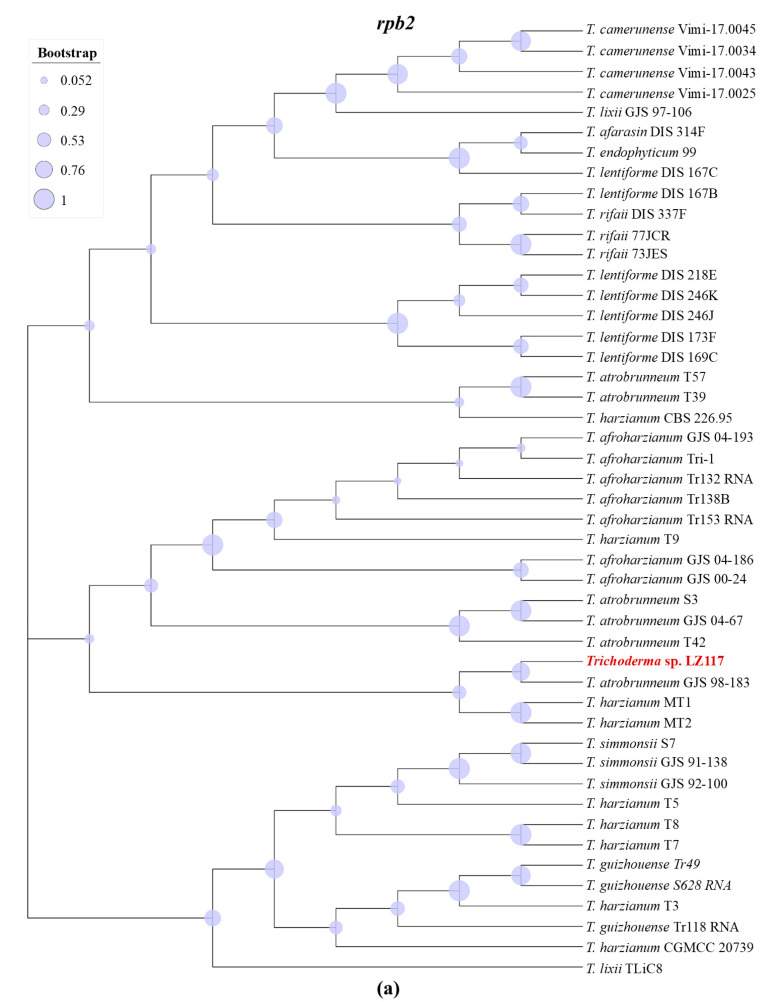

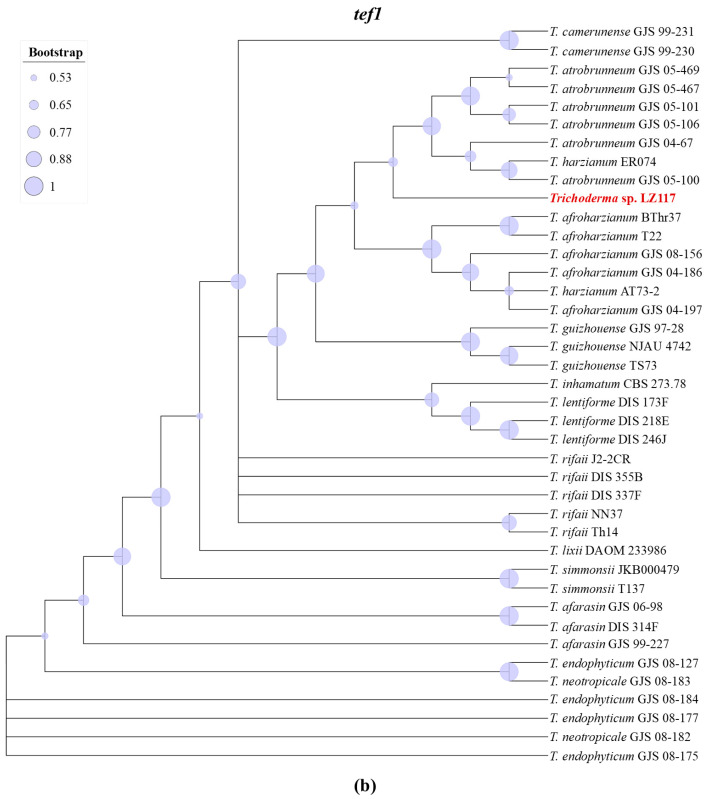
Phylogenetic analysis of *Trichoderma* sp. LZ117 and its closely related strains based on the sequences of *rpb2* (**a**) and *tef1* (**b**).

Firstly, the molecular identification of the LZ117 *Trichoderma* isolate was performed based on the cumulative analysis of the following three DNA barcodes: partial *tef1* and *rpb2* gene fragments via *Tricho*MARK (www.trichokey.com) (assessed on 28 January 2021), a website containing accessory tools for the accurate and precise identification of *Trichoderma* at the species level. The result (Supplementary Materials S3, Figure S2) showed that the LZ117 isolate belongs to a putative new species close to *T. atrobrunneum* (*Harzianum* clade) with a threshold sufficient for species identification at ≥99% for *rpb2* and ≥97% for *tef1* DNA barcode loci [41]. Maximum likelihood (ML) phylogenetic analyses were performed with the *rpb2* datasets of 47 *Trichoderma* spp. strains (Supplementary Materials S1) and *tef1* datasets of 40 *Trichoderma* spp. strains (Supplementary Materials S2). The phylogenetic results further revealed that the strain *Trichoderma* sp. LZ117 is evolutionarily close to *T. atrobrunneum* (Figure 1). 

**Figure 2:**
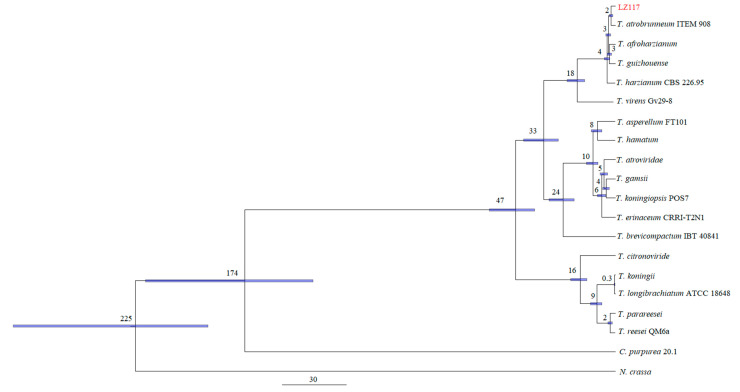
Phylogenomic trees based on 20 *Trichoderma* species inferred by the analysis of Beast2 CladeAge. Bayesian chronogram given in millions of years, based on the concatenated alignment of 576 orthologous proteins of *Trichoderma* species and two additional species added for time calibration. Numbers correspond to the estimated node age. Bars represent the 95% confidence interval in the time estimation based on the lognormal relaxed clock.

To further figure out which group *Trichoderma* sp. LZ117 is close to, we subjected 20 strains, including 17 other *Trichoderma* fungi and two phylogenetically more distant species—*Claviceps purpurea* and *Neurospora crassa*—to a time-scaled phylogenomic analysis using 576 orthologous genes (see Materials and Methods Section 2.3) [24]. The phylogenetic tree (Figure 2) showed that the LZ117 isolate, together with *T. atrobrunneum*, *T. afroharzianum*, and *T. guizhouense*, was speciated from *T. harzianum* around 4 Mya. In the period of the speciation of *T. afroharzianum* and *T. guizhouense* about 3 Mya, these two species were separated from *T. atrobrunneum* and the LZ117 isolate. The speciation of *T. atrobrunneum* and LZ117 can be dated to about 2–3 Mya, comparable to that between *T. reesei* and *T. parareesei*, indicating that they are separate species. Therefore, according to the latest comparison, the isolate, formerly known as *T. harzianum* LZ117 [12], is now reclassified as a new species close to *T. atrobrunnuem* (*Harzianum* clade).

### 3.2. Morphological Characterization

**Figure 3:**
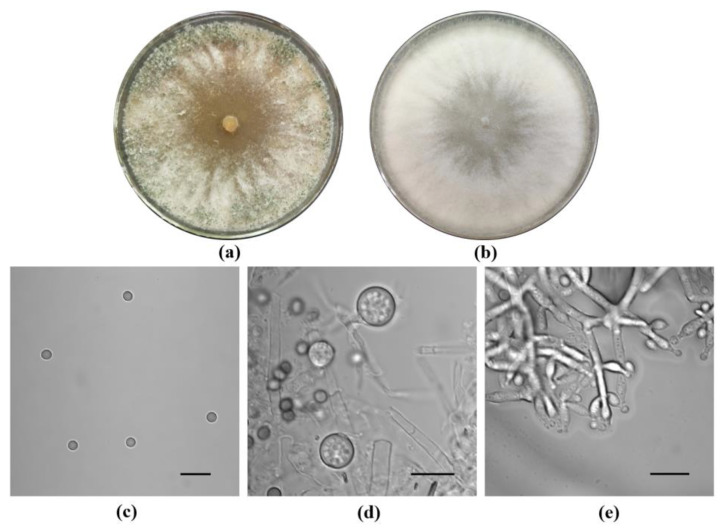
Morphology of *Trichoderma* sp. LZ117. Colony morphology on (**a**) MEA medium and (**b**) PDA after 7 days of growth at 28 °C. (**c**) Conidia, (**d**) chlamydospores, (**e**) conidiophores and phialides. Scale bars: 10 μm (**c**,**d**).

White hypha and dark green spores of *Trichoderma* sp. LZ117 were harvested after seven days of cultivation at 28 °C (Figure 3a). However, no conidia but abundant aerial mycelia were produced on the PDA medium, without pigment diffusion (Figure 3b). The conidia were about 3.0–3.5 μm, smooth, and subglobose to globose (Figure 3c). Abundant chlamydospores were also observed, with sizes of 5.0–9.0 μm (Figure 3d). The conidiophores comprised a central axis with side branches slightly inclined upward (Figure 3e). The phialides were almost stout to distinctly swollen and nearly ampulliform, commonly in whorls of two to three (Figure 3e).

### 3.3. Properties of the Trichoderma sp. LZ117 Genome 

The DNA from the evaluated isolate was extracted from the hypha and spores collected. The genome of LZ117 was sequenced by integrating the Nanopore PromethION platform and Illumina NovaSeq PE150 platform, generating 277,355 reads (Table 1), assembled for a total of 10 contigs, with a GC% of 47.38. Compared with the other *Trichoderma* genome references, the genomes of *Trichoderma* spp. (*T. atrobrunneum*, *T. harzianum*, *T. simmonsii*) belonging to the *T. harzianum* complex were similar in size and GC content (Table 2). Considering the genome sequencing coverage, even though LZ117 presented a medium value (90×), the genome assembled was of supreme quality, showing a lower degree of fragmentation than most previously assembled, with an N50 of 6,585,579 bp. The longest assembled fragment was 7,918,765 bp in length (performed by Unicycler), and the total assembly length was 41,121,236 bp. To assess the quality of the LZ117 genome assembly, we employed the BUSCO tool and a set of fungal conserved single-copy homologous genes as a reference [26]. The results demonstrated that among the 758 fungal conserved single-copy homologous genes, 751 were fully represented in the comparison. The comparison identified three genes as duplicates, and only four were missed. The completeness of the genome assembly reached 99.5% BUSCO (Table 1, Supplementary Materials S4), indicating that the genome assembly results were highly reliable and of excellent quality.

### 3.4. Gene Prediction and Classification

In the genome of *Trichoderma* sp. LZ117, with the assistance of the previously available transcriptome, a total of 25,846 transcripts were predicted, from which 12,707 protein-coding genes were predicted via Augustus and Funannotate (Supplementary Materials S5). According to the annotation from Gene Ontology (GO), genes in LZ117 are mainly involved in binding, catalytic activity, transporter activity, cells, cell parts, macromolecular complexes, organelle parts, biological regulation, cellular component organization or biogenesis, cellular processes, establishment of localization, localization, metabolic processes, regulation of biological processes, responses to stimuli, and signaling (Supplementary Materials S3, Figure S3).

Based on the statistical results of the functional annotation of the Eukaryotic Orthologous Groups (KOG) database, the encoding gene of *Trichoderma* sp. LZ117 is mainly involved in RNA processing and modification, energy production and conversion, amino acid transport and metabolism of amino acids, lipids, and carbohydrates, translation, ribosome structure and biogenesis, post-translational modifications, protein turnover, chaperons, general function mechanism, signal transduction mechanisms, intracellular transport, secretion, and vesicular transport. Typically, energy production and conversion, amino acid transport, translation, ribosome structure and biogenesis, secondary metabolites biosynthesis, transport, and catabolism account for the most matched genes among these function classes (Supplementary Materials S3, Figure S4). 

**Figure 4:**
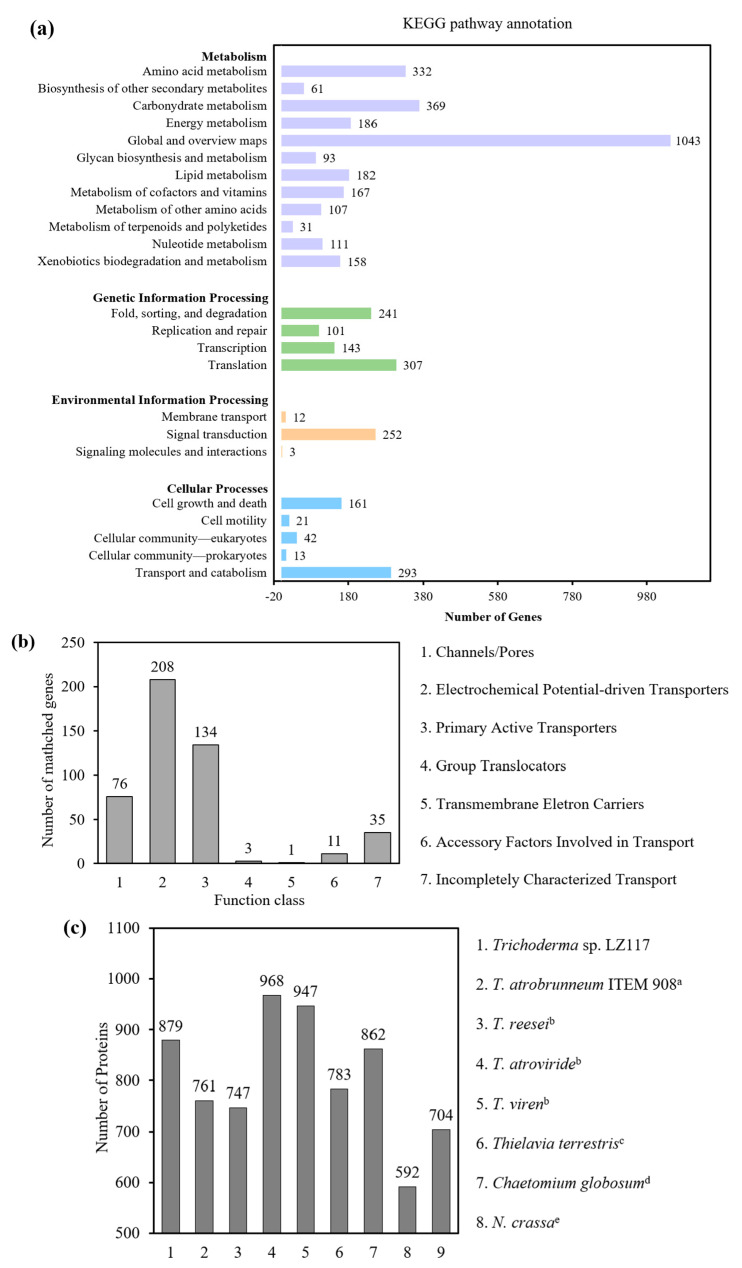
Functional annotation of *Trichoderma* sp. LZ117. (**a**) KEGG functional annotation of *Trichoderma* sp. LZ117. The y-axis indicates the enriched pathway names, and the x-axis denotes the number of coding genes in the enriched pathways. (**b**) TCDB functional annotation of *Trichoderma* sp. LZ117. The y-axis shows the number of matched coding genes, and the x-axis denotes the name of the function class. (**c**) Prediction of secreted proteins in *Trichoderma* sp. LZ117 and other *Trichoderma* spp. The y-axis indicates the number of the predicted proteins, and the x-axis denotes the name of the selected *Trichoderma* spp. ^a^ Data from Fanelli F, Liuzzi VC, et al., 2018 [10]. ^b^ Data from Druzhinina IS, Shelest E, et al., 2012 [48]. ^c^ Data from Berka RM, Grigoriev IV, et al., 2011 [49]. ^d^ Data from Cuomo CA, Untereiner WA, et al., 2015 [50]. ^e^ Data from Galagan JE, Calvo SE, et al., 2003 [51]. ^f^ Data from Galagan JE, Calvo SE, et al., 2005 [52].

The KEGG pathway enrichment analysis showed that the coding genes of LZ117 are closely related to the metabolism pathway (Figure 4a), especially to the global and overview maps and carbohydrate metabolism. The latter, according to numerous research studies, contributes to the production of lignocellulosic enzymes. In terms of genetic information processing, genes were enriched in translation, protein folding, sorting, and degradation, indicating the ability of LZ117 to secrete cellulase early may come from the protein regulation, and translational or post-translational regulators may play important roles in regulating the production of lignocellulosic enzymes.

#### 3.4.1. Transporters Annotated from TCDB and Initial Function Analysis

The annotation from the Transporter Classification Database (TCDB) showed that genes related to membrane transporters in LZ117 mainly involve channels/pores, electrochemical potential-driven transporters, and primary active transporters (Figure 4b).

In the previous comparative transcriptomic analysis of *Trichoderma* sp. LZ117 and *T. harzianum* K223452, a strain isolated from the forest soil in Heilongjiang Province (China) presented poor cellulase production, and most of the transporters in LZ117 were significantly downregulated. In contrast, the transcription level of the homologous transporter of M431DRAFT_480915 (*T. harzianum* CBS 226.95) was significantly increased [8], indicating that this putative transporter may be important in cellulase production. The transporter in *T. harzuanum* CBS226.95 is annotated as a maltose permease, which is highly conserved in filamentous fungi, with 99% and 87.6% homology to *T. harzuanum* CBS226.95 and *T. reesei* QM6a, respectively. The maltose permease has a conserved domain of the major facilitator superfamily (MFS), a large and diverse group of secondary transporters essential for moving multiple substrates across biomembranes [47]. It was found that the loss of maltose permease (TRIREDRAFT_65191) in cellulase hyperproduction strain *T. reesei* Rut-C30 had a serious impact on the absorption and assimilation of α-linked oligo- and poly-glucosides; as a result, the growth of the deletion mutant on dextrin, starch, maltose, and maltose was severely hindered [25]. This motivated us to study the effects of TRIREDRAFT_65191 on cellulase production, which is presented in the below section. 

#### 3.4.2. Predicted Secretome

A total of 879 proteins are potentially secreted, which is more than that of *T. atrobrunneum* ITEM 908 (761) and comparable to that of close *Trichoderma* species and other species [48] (Figure 4c). It should be noted that these comparisons were restricted by the different approaches for gene/protein models used in different genome projects. In addition, 1098 signal peptide structural proteins and 2189 transmembrane structural proteins were also predicted in the genome.

#### 3.4.3. CAZymes

*Trichoderma* is a model system for producing carbohydrate-active enzymes (CAZymes). This group comprises a list of modules that are classified in the CAZY database (www.cazy.org) (assessed on 26 September 2022) as belonging to different families, including glycoside hydrolases (GHs), which catalyze the hydrolysis and rearrangement of glycosidic bonds, glycosyl transferases (GTs), which are responsible for the formation of glycosidic bonds, polysaccharide lyases (PLs), which catalyze the non-hydrolytic cleavage of glycosidic bonds, carbohydrate esterases (CEs), which hydrolyze carbohydrate esters, and auxiliary activities (AAs), which are redox enzymes that act in conjunction with CAZymes. 

**Figure 5:**
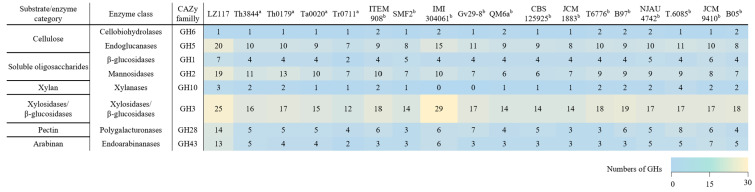
Quantitative comparison of glycoside hydrolases (GHs) related to biomass degradation in the genomes of the *Trichoderma* strains presented as a heatmap. ^a^ Data from Rosolen RR, Horta MAC, et al., 2023 [18]. ^b^ Data from Fanelli F, Liuzzi VC, et al., 2018 [10]. Th3844: *T. harzianum* IOC-3844; Th0179: *T. harzianum* CBMAI-0179; Ta0020: *T. atroviride* CBMAI-0020; Tr0711: *T. reesei* CBMAI-0711; SMF2: *T. longibrachiatum* SMF2; IMI 304061: *T. virens* IMI 304061; Gv29-8: *T. virens* Gv29-8; QM6a: *T. reesei* QM6a; CBS 125925: *T. parareesei* CBS 125925; JCM 1883: *T. koningii* JCM 1883; T6776: *T. harzianum* T6776; B97: *T. harzianum* B97; NJAU 4742: *T. guizhouense* NJAU 4742; T.6085: *T. gamsii* T.6085; JCM 9410: *T. atroviride* JCM 9410; B05: *T. asperellum* B05. The color indicates the enrichment extent of GH number as shown in the footnote of the heatmap.

According to the annotation of dbCAN2 meta server (https://bcb.unl.edu/dbCAN2/) (assessed on 26 September 2022) [53], 1062 domains are potentially associated with CAZymes, including 136 CBMs, 62 CEs, 504 GHs, 111 AAs, 237 GTs, and 12 PLs (Supplementary Materials S6). More CAZyme-encoding genes were predicted in *Trichoderma* sp. LZ117 (Supplementary Materials S3, Table S2) than in the other *Trichoderma* strains reported so far [10,18]. In particular, GHs, GTs, AAs, and CBMs were highly enriched in *Trichoderma* sp. LZ117 (Supplementary Materials S3, Table S2). Regarding cellulose degradation, in the LZ117 strain, genes encoding CAZymes belonging to the GH1, GH2, GH3, GH5, GH28, and GH43 families were also present in relatively larger numbers than the other strains (Figure 5), which may explain the outstanding cellulase production performance of this strain [8,12]. Additionally, significantly more members of the GH18 family enzymes (Supplementary Materials S3, Figure S5), which are related to chitin degradation, were found in *Trichoderma* sp. LZ117.

#### 3.4.4. Clusters of Secondary Metabolism

Generally, biosynthetic gene clusters can be classified into several classes, including nonribosomal peptide synthetase (NRPS); NRPS-like fragment (NRPS-like); type I polyketide synthase (T1PKS); NRPS, T1PKS; NRPS-like, T1PKS; ladderane; fungal-RiPP; terpene; indole; NRPS, T1PKS, beta-lactone; and terpene, NRPS-like [54]. To inspect the distribution of biosynthetic gene clusters encoding potential secondary metabolites, the antiSMASH program (version 2.0.2) [55] was used to predict the genome of *Trichoderma* sp. LZ117. Overall, ten kinds of secondary metabolism clusters were predicted in the LZ117 strain, which comprised 718 genes in total (Supplementary Materials S3, Figure S6). Among the clusters, the T1PKS class was the most represented cluster with 215 genes, followed by the NRPS class with 119 genes. A total of 61 gene clusters showing 100% similarity with the known clusters were identified (Supplementary Materials S3, Table S3).

### 3.5. Protoplast Formation of Trichoderma sp. LZ117

To further improve the bioproduction efficiency of *Trichoderma* sp. LZ117, the genetic manipulation platform of this strain should be established. Techniques for the genetic manipulation of *Trichoderma* species include transformation by shock waves, electroporation, biolistic transformation, Agrobacterium-mediated transformation, and polyethylene glycol (PEG)-mediated protoplast transformation [56]. Among these methods, PEG-mediated protoplast transformation is relatively simple in operation with lower cost and high transformation efficiency. Therefore, we established protoplast-mediated genetic transformation of *Trichoderma* sp. LZ117. 

Protoplast preparation is the fundamental step. Based on current PEG-mediated protoplast transformation applied on *Trichoderma* spp. [40,56], here, we optimized and fine-tuned the methods for protoplast preparation on LZ117. Briefly, after spore suspension was absorbed and cultured in liquid complete media (1% sucrose, 0.6% casamino acid, 0.6% yeast extract) at 28 °C at 150 rpm for 36 h, the mycelia were filtered and dried with sterile filter paper and transferred into an empty sterile 50 mL centrifuge tube. The “lysing enzyme from *Trichoderma harzianum*” (Sigma L1412, Berlin, Germany) is a general enzyme used to lyse the cell wall of *Trichoderma* spp. [40,56]. However, we found that the protoplasts could not be extracted using only this enzyme. Therefore, we combined the “lysing enzyme from *Trichoderma harzianum*” (Sigma L1412, Germany) with “lyticase from *Arthrobacter luteus*” (Sigma L2524, Berlin, Germany), a type of zymlolyase previously used for the isolation of protoplasts from different fungi [51], to prepare the protoplasts of LZ117. We found that 10 mg/mL of the lysing enzyme from *Trichoderma harzianum* (Sigma L1412, Germany) and 10 μL of the lyticase from *Arthrobacter luteus* (Sigma L2524, Germany) were enough for the protoplast extraction of LZ117. They were dissolved in 1 M sorbitol and filtered into a 50 mL centrifuge tube containing mycelium followed by an incubation at 30 °C, 90 rpm for 3.5 h. The lysed protoplasts were filtered via a micracloth (EMD Millpore, Germany) and collected by centrifugation under 4 °C, 5000 rpm for 10 min after microscopy (Supplementary Materials S3, Figure S7). Finally, the protoplasts were resuspended gently with STC solution (21.8 g sorbitol, 5 mL 1 M Tris-HCl (pH8.0), 0.5549 g CaCl_2_, filled with distilled water to 100 mL, sterilized in advance), with the final concentration of the protoplasts at 1 × 10^8^. After adding 7% DMSO (Dimethyl sulfoxide), the protoplasts were divided into 1.5 mL EP tubes, with a total of 100 μL per tube, and stored at −80 °C or directly used.

### 3.6. Overexpression of Maltose Permease Mal1 in Trichoderma sp. LZ117

To detect the feasibility and efficiency of the PEG-mediated protoplast transformation, we explored the function of the maltose permease Mal1 in LZ117. We constructed a knockout cassette with a hygromycin resistance label to delete the maltose permease coding gene *mal1* via protoplast transformation. However, no *mal1*-deletion transformant was screened. The underlying reason may be the low homologous recombination efficiency of LZ117, which is potentially regulated by DNA repair enzymes *ku70* or *mus53* [57,58]. The homologs of these DNA repair enzymes were also found in the genome of LZ117, and truncation may also improve the efficiency of homologous recombination. 

**Figure 6:**
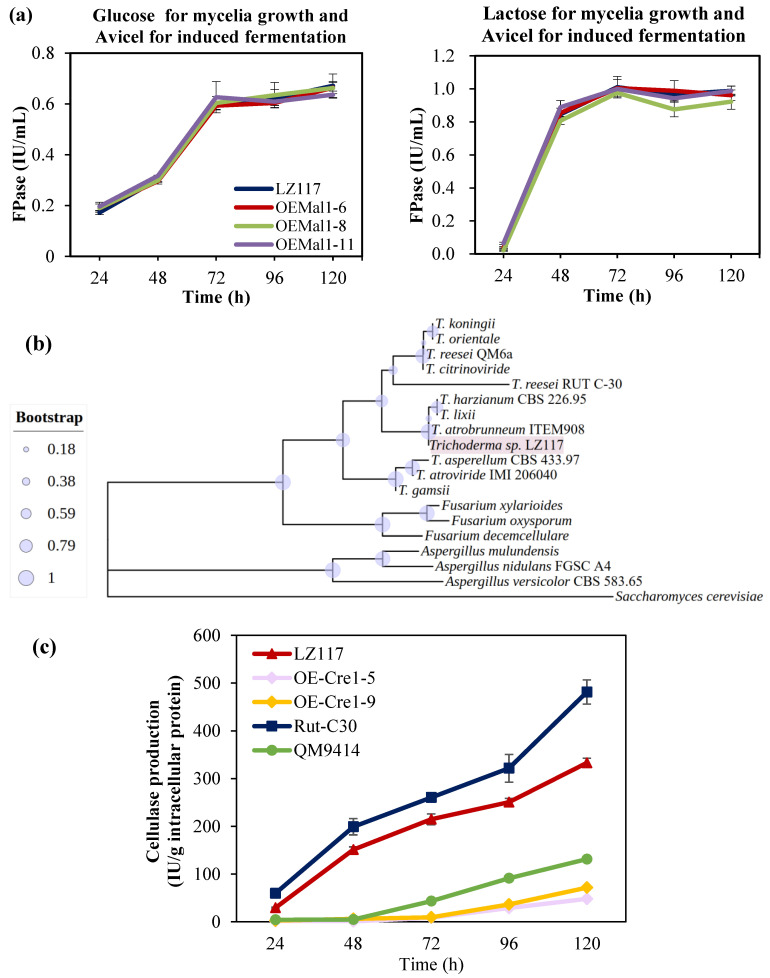
(**a**) For cellulase fermentation of the wildtype strain LZ117 and Mal1 overexpression transformants, glucose (left) and lactose (right) were used as the seed carbon sources, respectively, with Avicel as the inducer. (**b**) Phylogenetic tree of Cre1 from *Trichoderma*, *Aspergillus*, *Fusarium*, and *Saccharomyces cerevisiae*. (**c**) Analysis of cellulase production in *T. atrobunneum* LZ117, Cre1 overexpression strains (OE-Cre1-5 and OE-Cre1-9), *T. reesei* Rut-C30, and *T. reesei* QM9414. Glucose was used as the carbon source for seed preparation.

Subsequently, we overexpressed Mal1 in *Trichoderma* sp. LZ117 by random integration driven using the P*cbh1* promoter, which is a strong inducible promoter induced by cellulose, sophorose, and lactose [59]. More than 10 transformants were selected for the flask fermentation test, and the results for three of these transformants are shown in Figure 6a. The overexpression of maltose permease Mal1 in LZ117 did not lead to significant changes in cellulase production, regardless of whether the seeds were pre-incubated with glucose or lactose before transferring to Avicel for cellulase induction. 

During the cultivation and fermentation of *Trichoderma* sp. LZ117, the curves showed that carbon sources for the mycelia preparation exert significant impact on the cellulase production of LZ117. Compared with glucose (favorable carbon source), which served as the seed carbon source, the cellulase activity increased by around 60% when lactose, a less-favorable carbon source, served as the seed carbon source before the hyphae were transferred to Avicel for cellulase induction (Figure 6a). This result suggests that carbon catabolite repression (CCR) might profoundly influence the cellulase production in *Trichoderma* sp. LZ117.

### 3.7. Carbon Catabolite Repression (CCR) in Trichoderma sp. LZ117

It has been shown that during cellulase production, the presence of glucose can trigger Cre1-mediated CCR via signal transduction, resulting in the downregulation of the related cellulolytic enzymes in *T. reesei* [60]. The amino acid sequence of *Trichoderma* sp. LZ117-annotated Cre1 was used to conduct a BLASTp search for homologs from *Trichoderma* species (including *T. atrobrunneum* ITEM908, *T. asperellum* CBS433.97, *T. atroviride* IMI206040, *T. citrinoviride*, *T. gamsii*, *T. harzianum* CBS226.95, *T. koningii*, *T. lixii*, *T. orientale*, *T. reesei* QM6a, and *T. reesei* Rut C-30), *Fusarium* species (*F. xylarioides*, *F. decemcellulare*, and *F. oxysporum*), *Aspergillus* species (*A. mulundensis*, *A. nidulans* FGSC A4, and *A. versicolor* CBS583.65), and *Saccharomyces cerevisiae* (Supplementary Materials S7). Phylogenetic analyses and further polishing were performed to determine the evolutionary pattern of Cre1 using MEGA7 and the iTOL platform, respectively. Results from the analyses revealed that Cre1 from *Trichoderma* sp. LZ117 shares a common evolutionary clade with Cre1 identified in *T. atrobrunneum* ITEM908, which is evolutionarily close to that identified in *T. harzianum* CBS226.95 and *T. lixii*. Cre1 from *Trichoderma* sp. LZ117, but evolutionarily distant from that identified in *F. xylarioides*, *F. oxysporum*, *F. decemcellulare*, *A. mulundensis*, *A. nidulans* FGSC A4, *A. versicolor* CBS583.65, and *S. cerevisiae* (Figure 6b).

To further explore the influence of CCR on cellulase production in *Trichoderma* sp. LZ117, Cre1 was overexpressed under the control of inducible promoter P*cbh1* in the LZ117 strain (Supplementary Materials S3, Figure S8). All mycelia were cultured in glucose before Avicel-induced fermentation. The cellulase production of the wildtype LZ117 was comparable to that of the cellulase-hyperproducing strain *T. reesei* Rut-C30 (Figure 6c). In *T. reesei* Rut-C30, Cre1 was truncated, resulting in a reduced CCR effect [61], while both LZ117 and *T. reesei* Rut-C30 exhibited more superior cellulase production than *T. reesei* QM9414, in which Cre1 is intact (Figure 6c). The results further confirmed the robust natural enzyme production performance of LZ117. Interestingly, the overexpression of Cre1 in LZ117 showed a dramatic decrease in cellulase activity (Figure 6c). The results suggested that despite *Trichoderma* sp. LZ117′s natural superiority in cellulase production, similar to *T. reesei*, Cre1-mediated CCR remains a significant regulation repressor in the cellulase production of LZ117. 

## 4. Discussion

The genus *Trichoderma* (Ascomycota, Sordariomycetes, Hypocreales) is ubiquitously distributed in varied ecosystems [62], such as soil, wood, plant tissues, and so on. Studies and applications of *Trichoderma* spp. have contributed multiple facets to human life. In agriculture, *Trichoderma* species, especially *T. harzianum*, *T. viride*, *T. citrinoviride*, *T. brevicompactum*, *T. asperellum*, *T. longibrachiatum*, *T. asperellum*, and *T. koningii*, have served as useful reagents for biocontrol because of their superior ability in controlling plant diseases caused by fungi, insects, and nematodes [63]. In industrial applications, *Trichoderma* species also play a crucial role in producing enzymes and bioactive compounds [64,65,66]. It is therefore of great interest to explore novel *Trichoderma* species and strains for biotechnology applications. 

Both morphology and growth characteristics are traditional criteria for identifying *Trichoderma* [67]. With the increased isolation of novel *Trichoderma* strains, the difficulties in distinguishing them have also increased. The introduction of DNA barcoding, internal transcribed spacer (ITS), the gene encoding the second largest nuclear RNA polymerase subunit (*rpb2*), and the gene encoding translation elongation factor 1-alpha (*tef1*), has standardized and enhanced reliability in *Trichoderma* identification, becoming indispensable in recognition [23,68]. Based on these loci, new *Trichoderma* species can be identified using the criteria ITS ≥ 76%, *rpb2* ≤ 99%, and *tef1* ≤ 97%, which have been established as a reliable tool for molecular identification of *Trichoderma* that is accessible through the online tool TrichoMARK 2020 (https://trichokey.com/index.php/trichomark) (assessed on 28 January 2021) [23]. Even though only 10% of *Trichoderma* species have undergone WGS to date, WGS can provide sufficient data to resolve the evolutionary positions of species, especially those with non-concordant phylogenies between *rpb2* and *tef1*, despite sharing ITS sequences [23,24,69]. This approach also offers insights into the evolutionary history of the genus *Trichoderma*.

The strain *Trichoderma* sp. LZ117, which distinguished itself as a superior cellulase producer, was isolated from a moss surface in Tibet, China [12]. However, its previous identification as *T. harzianum* based solely on the DNA barcoding locus ITS might have been less precise [23]. To achieve precise species identification, we used the online tool TrichoMARK for re-evaluation. According to this protocol, the similarities in *rpb2* and *tef1* to reference sequences were 98.15% and 96.66%, respectively, suggesting that isolate LZ117 represents a potentially new species. Then, *rbp2* and *tef1* datasets along with a time-scaled phylogenomic analysis based on WGS were employed for further phylogenetic analysis to elucidate the position of the new species. In this study, we conducted a time-scaled phylogenomic analysis following the method used by Kubicek et al. (2019), in which 12 common species of *Trichoderma* (13 strains) and two phylogenetically more distant Sordariomycetes—*N. crassa*, *C. globosum*—were subjected using 638 orthologous genes [24]. In addition to the 12 species, we also added *T. atrobrunneum,* which is the closest species of LZ117 according to the comparison results based on TrichoMARK and the phylogenetic tree of *rpb2* and *tef1*. In addition, five other species that were reported to be isolated in China, including *T. koningiopsis*, *T. erinaceum*, *T. brevicompactum*, and *T. koningii,* were also included. Compared with the 12 species previously analyzed by Kubicek et al. (2019) [24], the evolution timing at the corresponding branches was delayed. This discrepancy might be because this study used only two ancestral nodes for time calibration, whereas the reported work utilized four nodes including (1) a common ancestral node of the order Hypocreales calibrated for a central 95% range of 190–196 Mya, (2) a common ancestral node between the families Hypocreaceae, Ophiocordycipitaceae, and Clavicipitaceae calibrated for a central 95% range of 162–168 Mya, (3) a common ancestral node of the Clavicipitaceae crown group for a central 95% range of 114–120 Mya, and (4) a common ancestral node of the Nectriaceae crown group for a central 95% range of 122–128 Mya [24]. Nonetheless, the 12 species remained clustered together, as observed previously, suggesting that the evolutionary analysis here is reliable. The speciation of *Trichoderma* sp. LZ117 and *T. atrobrunneum* can be dated to around 2 million years ago, comparable to the divergence between *T. reesei* and *T. parareesei*, supporting their classification as distinct species [9,10,24]. Therefore, *Trichoderma* sp. LZ117 can be identified as a separate species closely related to *T. atrobrunneum* within the *Harzianum* clade. At least eight infrageneric clades are included in the genus *Trichoderma*, of which *the Harzianum* clade is large [70]. Recently, several other new species belonging to *the Harzianum* clade have also been found and identified in China, such as *T. densissimum*, *T. paradensissimum* [71], *T. auriculariae*, *T. pholiotae* [61], *T. lentinulae*, *T. xixiacum*, *T. vermifimicola*, and *T. zelobreve* [72]. The discovery of these *Trichoderma* species could promote studies on the taxonomy and application of these species in cellulase production and agriculture applications [73]. 

In terms of genome size, *Trichoderma* sp. LZ117 is comparable to *T. atrobrunneum* and *T. harzianum*, with a wide range of putative CAZymes predicted. Compared with the currently reported *Trichoderma* species, LZ117 exhibited a high number of GH families involved in biomass degradation, specifically GH families 1, 2, 3, 5, 28, and 43. This is consistent with the potentially superior enzyme activity of this strain, even though not all of the predicted CAZymes are necessarily part of the secretome, and the number of genes associated with biomass degradation does not always correlate with the extent of degradation [18,74]. The efficient conversion of lignocellulosic biomass is crucial for sustainable economic development, yet the high cost of cellulase limits the industrial application of related technologies. Breeding cellulase-hyperproducing strains is integral to improving biorefinery [75]. *Trichoderma* sp. LZ117 shows promise for efficient biomass degradation, which underscores its potential to reduce cellulase production costs. In addition, the genomes of *Trichoderma* species generally contain a high number of chitinolytic genes, which are related to the mycoparasitic characteristics of fungi [24]. Specifically, the GH18 family is notably abundant in *T. harzianum*, *T. virens*, *T. atroviride*, *T. gamsii*, *T. asperellum*, and *T. atrobrunneum* [10]. Similarly, *Trichoderma* sp. LZ117 also harbors a significant number of GH18 genes, indicating the strain’s considerable potential for mycoparasitic activity or biocontrol ability. In addition, secondary metabolites from *Trichoderma* species usually exhibit antifungal activity against pathogens [60]. AntiSMASH analysis revealed a wide array of secondary metabolites produced by *Trichoderma* sp. LZ117, including several associated with antifungal properties, such as coelicheli [76]. In addition, several secondary metabolites produced by *Trichoderma* sp. LZ117 are also relevant to human healthcare, such as himastatin [77] and antimycin [78], indicating a broad potential application of this strain. 

Transporters play essential roles in cellulase production. Sugar transporters facilitate the uptake of monosaccharides, disaccharides, and oligosaccharides released from cellulose by cellulase into cells, thereby triggering the expression of cellulase-related genes [79,80]. In *T. reesei*, the absence of transporter Ctr1 negatively influenced the expression of cellulase genes under cellulose or lactose induction [81]. Maltose permease Mal1 was differentially upregulated compared with a poor cellulase-production strain *T. harzianum* K223452 under a cellulase-induced condition in previous work [8]. For the purpose of exploring the function of the transporter maltose permease Mal1 and further enhancing cellulase production in LZ117, we genetically manipulated LZ117 through PEG-mediated protoplast transformation. However, we failed to screen out the *mal1* deletion mutant, which may be due to the low homologous recombination efficiency regulated by DNA repair enzymes *ku70/ku80* or *mus53* in LZ117. To facilitate the further genetic manipulation of *Trichoderma* sp. LZ117, knockout or knockdown of *ku70/80* or *mus53*, which regulate non-homologous end joining, would be beneficial [51,52,82]. Homologous recombination and non-homologous end joining are the two main pathways to repair double-strand breaks [83], while non-homologous end joining is the dominant mechanism in most eukaryotic organisms [84]. Therefore, we overexpressed the maltose permease Mal1 randomly to evaluate the potential function in cellulase production. However, no significant cellulase enhancement was observed in the transformants. This result indicated that the basal level of Mal1 in LZ117 may be adequate for cellulase synthesis, whereas excessive Mal1 is unnecessary for enhancing cellulase production. While Mal1 plays a crucial role in the absorption and assimilation of α-linked oligo- and poly-glucosides in *T. reesei* [25], its function in *Trichoderma* sp. LZ117 may differ because of species specificity. 

In this study, we found that CCR may be a key factor in restricting the cellulase production of *Trichoderma* sp. LZ117. As a regulatory circuit widely conserved among microorganisms, CCR is involved in many biological events including adaption to the environment, the survival of microorganisms [85], plant–microbe interactions [86], and the synthesis of enzymes involved in lignocellulosic degradation [60]. In filamentous fungi, transcriptional repressor Cre1 (catabolite responsive element 1), a homolog of Mig1 in *Saccharomyces cerevisiae*, plays a crucial role in CCR [85]. And Cre1-mediated CCR is another key restriction for cellulase production [87]. In a previous study, compared with *T. harzianum* K223452, which exhibited poor cellulase production, Cre1 in *Trichoderma* sp. LZ117 showed a lower expression level [8]. The low expression level of Cre1 may be one of the reasons for the high cellulase production observed in the wildtype LZ117, comparable to cellulase-hyperproducing strain *T. reesei* Rut-C30. The low expression level of Cre1 may relieve the CCR level in restricting cellulase production. However, in this study, we found that the type of carbon source for seed preparation still has a profound impact on the outcomes of cellulase-induced fermentation, and the overexpression of Cre1 in LZ117 significantly suppressed cellulase production, highlighting that Cre1-mediated CCR still occupies a pivotal status in this strain.

In summary, we conclude that *Trichoderma* sp. LZ117 is a new species belonging to the *Harzianum* clade, and provide insight into its natural biosynthetic potentials by genome mining. The establishment of the genetic manipulation system of LZ117 benefits further metabolic engineering of this promising strain for various biotechnology applications. As a promising cellulase hyperproducer, *Trichoderma* sp. LZ117 serves as a good model to reveal the regulatory mechanism for cellulase induction and production and has great potential to be further developed as a robust cellulase producer for the bioconversion of lignocellulosic biomass. 

## Figures and Tables

**Table 1:** Genome-wide statistics of *Trichoderma* sp. LZ117 sequencing, assembly, and assessing results.

	Item	Count
Sequencing	Total sequenced bases	6,784,405,156 bp
	Number of reads	277,355
	Mean read length	24,461 bp
	N50 reads	29,320
	Mean read quality	9.5
Assembly	N50_length	6,585,579 bp
	Number of contigs	10
	Number of scaffolds	9
	Genome size	41,121,336 bp
	GC content	47.38%
	Predicted genes	12,707
Assessing	Complete and single-copy BUSCOs (S)	751 (99,1%)
	Complete and duplicated BUSCOs (D)	3 (0.4%)
	Missing BUSCOs (M)	4 (0.5%)
	Complete BUSCOs (C)	754 (99.5%)

**Table 2:** Comparison of the genome features of *Trichoderma* spp. genomes.

Species	Strain	Coverage	Genome Size (Mb)	GC Content (%)	N50 Reads	Assembly Level	Genes	Reference
*Trichoderma* sp.	LZ117	92×	41	47.4	6,585,579	Contigs (10)	12,707	This study
*T. atrobrunueum*	ITEM 908	60×	39	49.18	129,299	Scaffolds (804)	8649	[10]
*T. atroviride*	CBMAI-0020	229×	36	49.5	3,146,023	Contigs (14)	10,082	[18]
*T. atroviride*	IMI206040	8×	36	49.7	2,007,903	Contigs (29)	11,809	[42]
*T. harzianum*	IOC-3844	164×	40	47.5	3,607,994	Contigs (15)	10,786	[18]
*T. harzianum*	CBMAI-0178	219×	39	49.4	2,983,622	Contigs (18)	11,322	[18]
*T. harzianum*	T6776	85×	39	48.5	68,846	Scaffolds (1572)	11,501	[43]
*T. harzianum*	CBS 226.95	120×	41	47.6	2,414,909	Scaffolds (532)	14,269	-
*T. virens*	Gv29-8	8×	39	49.2	1,836,662	Scaffolds (93)	12,405	[42]
*T. simmonsii*	GH-Sj1	-	40	48.1	6,451,197	Scaffolds (7)	13,296	[44]
*T. reesei*	QM6a	80×	35	51.0	18,236	Chromosomes (7)	10,877	[45]
*T. reesei*	QM6a	9×	33	52.8	1,219,543	Scaffolds (77)	9109	[46]

## Data Availability

The assembled genome sequence *Trichoderma* sp. LZ117 was deposited in DDBJ/ENA/GenBank under the BioProject accession number PRJNA1105021 (https://dataview.ncbi.nlm.nih.gov/object/PRJNA1105021?reviewer=rjj2iol6bp232arov7o0dd55rl) (assessed on 26 April 2024). Transcriptome data of *Trichoderma* sp. LZ117 is available with an accession number PRJNA613881.

## Supplementary Materials

The following supporting information can be downloaded at https://www.mdpi.com/article/10.3390/jof10100697/s1: Supplementary Materials S1–S7.
