# Supplementary material for: Genomic Characterization and Establishment of a Genetic Manipulation System for Trichoderma sp. (Harzianum Clade) LZ117
Source: J Fungi (Basel). 2024 Oct 7;10(10):697. doi: 10.3390/jof10100697 (PMC11508783; doi:10.3390/jof10100697)
Supplement: Supplementary file 1 [file jof-10-00697-s001.zip › Supplementary Materials S1.pdf]

>*Trichoderma sp.* LZ117
AGGAAATCTGTGGCGGTCCTGCTGGCCAGCTGTTCCGTGGTATCATGCGAAGGATGAA
CACTGAGTTGGCCAACTACCTGAGACGATGCGTTGAGGGCAACCGACATTTCAACCTT
GCTGTTGGTATCAAGCCCGGCACGCTTTCAAACGGATTGAAGTATTCGCTTGCCACAG
GCAACTGGGGTGATCAGAAGAAGGCCATGAGCTCAACTGCAGGTGTGTCCCAGGTGC
TTAACCGATACACGTTTGCTTCGACCCTGTCACATTTGCGTCGTACCAACACTCCCATC
GGAAGAGATGGTAAGCTGGCAAAGCCTCGACAGCTTCACAACACGCATTGGGGTTTG
GTCTGCCCAGCCGAGACACCCGAAGGACAGGCCTGTGGTTTGGTCAAGAACTTGTCT
TTGATGTGTTACGTCAGTGTCGGTTCTCCCTCTGAACCTCTCATTGAGTTCATGATCAAC
AGAGGTATGGAAGTCGTCGAAGAGTACGAGCCTCTGCGGTATCCTCATGCTACAAAGA
TTTTTGTGAACGGTGTCTGGGTTGGAGTCCACCAAGACCCTAAGCACTTGGTGAACCA
GGTTCTGGATACTCGTCGCAAGTCCTATCTGCAATACGAAGTCTCTCTGGTGAGAGAAA
TTCGAGATCAGGAATTCAAAATCTTTTCCGACGCTGGCCGTGTCATGCGACCAGTCTTT
ACCGTTCAGCAGGAAGATGACCCGGAAACGGGCATCAACAAGGGTCACCTGGTATTG
ACCAAGGAGCTCGTCAATAGGTTGGCCAAGGAGCAGGCTGAACCTCCGGAAGACCCC
AGCATGAAGATTGGATGGGAGGGATTGATCAGGGCTGGTGCGGTTGAATATCTCGACG
CCGAGGAAGAGGAGACGTCCATGATCTGCATGACGCCAGAGGATCTCGAGCTGTATCG
TCTTCAGAAGGCCGGTATTAACACTGAGGAAGACATGGGAGATGACCCGAACAAGCG
ACTGAAGACCAAGACCAACCCGACAACTCACATGTACACCCATTGCGAGATTCACCCA
AGTATGATCTTAGGTATCTGTGCTAGTATCATTCCTTTCCCCGATCACAACCAGGTATGT
TGTCCACCCTTCCAGTCTTATAAACGAAGAAAAAATACTAATGGTGTGCATATCCCCGG
TACCCCC
>OP709971.1 *Trichoderma guizhouense* Tr49
TGCTGGCTAAGCTGTTCCGTGGTATCATGCGAAGGATGAACACTGAGTTGGCCAACTAT
CTGAGACGTTGCGTTGAGGGTAACCGACACTTCAACCTGGCTGTGGGTATCAAGCCCG
GCACTCTTTCAAACGGATTGAAGTATTCGCTTGCCACAGGAAACTGGGGTGATCAGAA
GAAGGCCATGAGCTCAACTGCTGGTGTGTCCCAGGTGCTTAACCGTTACACATTTGCTT
CGACCTTGTCACATTTGCGTCGTACCAACACCCCTATCGGAAGAGATGGTAAGCTGGC
AAAGCCTCGACAGCTTCACAACACGCATTGGGGTTTGGTCTGCCCAGCCGAGACACC
CGAAGGACAGGCTTGTGGTCTGGTCAAGAACTTGTCTTTGATGTGTTACGTCAGTGTC
GGTTCTCCCTCCGAACCTCTGATTGAGTTCATGATCAACAGAGGTATGGAAGTCGTCGA
AGAGTACGAGCCGCTGCGGTATCCTCATGCTACGAAGATTTTCGTGAACGGTGTCTGG
GTTGGAGTCCACCAAGACCCTAAGCACTTGGTGAACCAGGTCCTGGACACTCGTCGC
AAGTCCTATCTGCAATACGAAGTCTCTCTCGTGAGAGAAATTCGAGACCAGGAATTCA
AAATCTTTTCCGACGCAGGCCGTGTCATGCGACCAGTCTTTACCGTTCAGCAGGAAGA
TGACCCGGAAACGGGCATCAACAAGGGCCACCTGGTATTGACCAAGGAGCTCGTCAA
TAGATTGGCCAAGGAGCAGGCTGAGCCTCCGGAAGACCCCAGCATGAAGATTGGATG
GGAGGGATTGATTAGGGCTGGTGCAGTTGAATATCTCGACGCCGAGGAAGAGGAGAC
GTCCATGATCTGCATGACGCCAGAGGATCTCGAGCTGTATCGTCTTCAGAAGGCCGGTA
TTAACACTGAGGAAGACATGGGAGATGATCCGAACAAGCGACTGAAGACCAAGACGA
ACCCGACAACTCACATGTACACCCATTGCGAGATTCACCCAAGTATGATCTTAGGTATC
TGTGCTAGTATCATTCCTTTCCCCGATCACAACCAGGTATGTTGTCC

>KJ665273.1 *Trichoderma guizhouense* S628

TGCTGGCTAAGCTGTTCCGTGGTATCATGCGAAGGATGAACACTGAGTTGGCCAACTAT
CTGAGACGTTGCGTTGAGGGTAACCGACACTTCAACCTGGCTGTGGGTATCAAGCCCG
GCACTCTTTCAAACGGATTGAAGTATTCGCTTGCCACAGGAAACTGGGGTGATCAGAA
GAAGGCCATGAGCTCAACTGCTGGTGTGTCCCAGGTGCTTAACCGTTACACATTTGCTT
CGACCTTGTCACATTTGCGTCGTACCAACACCCCTATCGGAAGAGATGGTAAGCTGGC
AAAGCCTCGACAGCTTCACAACACGCATTGGGGTTTGGTCTGCCCAGCCGAGACACC
CGAAGGACAGGCTTGTGGTCTGGTCAAGAACTTGTCTTTGATGTGTTACGTCAGTGTC
GGTTCTCCCTCCGAACCTCTGATTGAGTTCATGATCAACAGAGGTATGGAAGTCGTCGA
AGAGTACGAGCCGCTGCGGTATCCTCATGCTACGAAGATTTTCGTGAACGGTGTCTGG
GTTGGAGTCCACCAAGACCCTAAGCACTTGGTGAACCAGGTCCTGGACACTCGTCGC
AAGTCCTATCTGCAATACGAAGTCTCTCTCGTGAGAGAAATTCGAGACCAGGAATTCA
AAATCTTTTCCGACGCAGGCCGTGTCATGCGACCAGTCTTTACCGTTCAGCAGGAAGA
TGACCCGGAAACGGGCATCAACAAGGGCCACCTGGTATTGACCAAGGAGCTCGTCAA
TAGATTGGCCAAGGAGCAGGCTGAGCCTCCGGAAGACCCCAGCATGAAGATTGGATG
GGAGGGATTGATTAGGGCTGGTGCAGTTGAATATCTCGACGCCGAGGAAGAGGAGAC
GTCCATGATCTGCATGACGCCAGAGGATCTCGAGCTGTATCGTCTTCAGAAGGCCGGTA
TTAACACTGAGGAAGACATGGGAGATGATCCGAACAAGCGACTGAAGACCAAGACGA
ACCCGACAACTCACATGTACACCCATTGCGAGATTCACCCAAGTATGATCTTAGGTATC
TGTGCTAGTATCATTCCTTTCCCCGATCACAACCAGGTATG

>OP709972.1 *Trichoderma guizhouense* Tr118
TGCTGGCTAAGCTGTTCCGTGGTATCATGCGAAGGATGAACACTGAGTTGGCCAACTAT
CTGAGACGGTGCGTTGAGGGCAACCGACACTTCAACCTGGCTGTGGGTATCAAGCCTG
GCACACTTTCAAACGGATTGAAGTATTCGCTTGCCACAGGAAACTGGGGTGATCAGAA
GAAGGCCATGAGCTCAACTGCTGGTGTGTCCCAGGTGCTTAACCGTTACACATTTGCTT
CGACCTTGTCACATTTGCGTCGTACCAACACCCCTATCGGAAGAGATGGTAAGCTGGC
AAAGCCTCGACAGCTTCACAACACGCATTGGGGTTTGGTCTGCCCAGCCGAAACACC
CGAAGGACAGGCCTGTGGTCTGGTCAAGAACTTGTCTTTGATGTGTTACGTCAGTGTC
GGTTCTCCCTCCGAACCTCTGATTGAGTTCATGATCAACAGAGGTATGGAAGTCGTCGA
AGAGTACGAGCCGCTGCGGTACCCTCATGCTACAAAGATTTTCGTGAATGGTGTCTGG
GTTGGAGTTCACCAAGACCCTAAGCACTTGGTGAACCAGGTCCTGGACACTCGTCGCA
AGTCCTATCTGCAATACGAAGTCTCTCTCGTGAGAGAAATTCGAGACCAGGAATTTAA
AATCTTTTCCGACGCAGGCCGTGTCATGCGACCAGTCTTTACCGTTCAGCAGGAAGAT
GACCCGGAAACGGGCATCAACAAGGGCCACCTGGTATTGACCAAGGAGCTCGTCAAT
AGATTGGCCAAGGAGCAGGCTGAGCCTCCGGAAGACCCCAGCATGAAGATTGGATGG
GAGGGATTGATTAGGGCTGGTGCAGTTGAATATCTCGACGCCGAGGAAGAGGAGACGT
CCATGATCTGCATGACGCCAGAGGATCTCGAGCTGTATCGTCTTCAGAAGGCCGGTATT
AACACTGAGGAAGACATGGGAGATGATCCGAACAAGCGACTGAAGACCAAGACGAA
CCCGACAACTCACATGTACACCCATTGCGAGATTCACCCAAGTATGATCTTAGGTATCT
GTGCTAGTATCATTCCTTTCCCCGATCACAACCAGGTATGTTGTCC

>MG917680.1 *Trichoderma harzianum* T9
GGTCCCNTGCTGGCCAAGCTGTTCCGTGGTATCATGCGAAGGATGAACACTGAGTTGG
CCAACTACCTGAGACGATGCGTTGAGGGCAACCGACACTTCAACCTGGCTGTTGGTAT

CAAGCCCGGCACGCTTTCAAACGGACTGAAGTATTCGCTTGCCACAGGAAACTGGGG
TGATCAGAAGAAGGCCATGAGCTCAACTGCCGGTGTGTCCCAGGTGCTTAACCGTTAC
ACGTTTGCTTCGACCTTGTCACATTTGCGTCGTACCAACACTCCTATCGGGAGAGATGG
TAAGCTGGCGAAGCCTCGACAGCTTCACAACACGCATTGGGGCTTGGTCTGCCCAGCC
GAGACACCCGAAGGACAGGCCTGTGGTCTGGTCAAGAACTTGTCTTTGATGTGTTACG
TCAGTGTCGGTTCTCCCTCCGAGCCTCTGATTGAGTTCATGATCAACAGAGGTATGGAA
GTCGTCGAAGAGTACGAGCCGCTGCGGTATCCTCATGCTACAAAGATTTTTGTGAACG
GTGTCTGGGTTGGAGTTCACCAAGACCCTAAGCACTTGGTGAACCAGGTTCTGGATAC
TCGTCGCAAGTCCTATCTGCAATACGAAGTCTCTCTCGTGAGAGAAATTCGAGACCAG
GAATTCAAAATCTTTTCCGATGCAGGTCGTGTCATGCGACCAGTCTTTACCGTTCAGCA
GGAAGATGATCCGGAAACGGGCATCAACAAGGGCCACCTGGTATTGACCAAGGAGCT
CGTCAATAGATTGGCCAAGGAGCAGGCTGAGCCTCCGGAAGACCCCAGCATGAAGATT
GGATGGGAGGGATTGATCAGGGCTGGTGCGGTTGAATATCTCGACGCCGAGGAAGAG
GAGACGGCCATGATCTGCATGACACCAGAGGATCTCGAGCTGTATCGTCTTCAGAAGG
CCGGTATCAACACTGAGGAAGACATGGGAGATGATCCGAACAAGCGACTCAAGACCA
AGACGAACCCGACAACTCACATGTACACCCATTGCGAGATTCACCCAAGTATGATCTTA
GGTATCTGTGCTAGTATCATTCCTTTCCCCGATCACAACCAGGTATGTTGTCCACGCTTT
CAGTCTTATGAACGAAGAAAAAAACTAATGGTGTGCATATCCCC

>MT586212.1 *Trichoderma harzianum* MT1
CAGCTGTTCCGTGGCATCATGCGAAGGATGAACACTGAGTTGGCCAACTACCTGAGAC
GATGCGTTGAGGGCAACCGACACTTCAACCTTGCTGTTGGTATCAAGCCCGGCACGCT
TTCAAACGGATTGAAGTATTCGCTTGCCACAGGCAACTGGGGTGATCAGAAGAAGGCC
ATGAGCTCAACTGCAGGTGTGTCCCAGGTGCTTAACCGATACACGTTTGCTTCGACCCT
GTCACATTTGCGTCGTACCAACACTCCCATCGGAAGAGATGGTAAGCTGGCAAAGCCT
CGACAGCTTCACAACACGCATTGGGGTTTGGTCTGCCCAGCCGAAACACCCGAAGGA
CAGGCCTGTGGTCTGGTCAAGAACTTGTCTTTGATGTGTTACGTCAGTGTCGGTTCTCC
CTCTGAGCCTCTAATTGAGTTCATGATCAACAGAGGCATGGAAGTCGTCGAAGAGTAC
GAGCCGCTGCGGTATCCTCATGCTACAAAGATTTTTGTGAACGGTGTCTGGGTTGGAGT
TCACCAAGACCCTAAGCACTTGGTGAACCAGGTCCTGGACACTCGTCGCAAGTCCTAT
CTGCAGTACGAAGTCTCTCTTGTGAGAGAAATTCGAGACCAGGAATTCAAAATCTTTT
CCGACGCTGGTCGTGTCATGCGACCAGTCTTTACCGTTCAGCAGGAAGATGACCCGGA
AACGGGCATCAACAAGGGCCACCTGGTATTGACCAAGGAACTCGTCAATAGATTGGCC
AAGGAGCAGGCTGAGCCTCCGGAAGACCCCAGCATGAAGATTGGATGGGAGGGATTG
ATTAGGGCTGGTGCAGTTGAATATCTCGACGCCGAGGAAGAGGAAACGTCCATGATCT
GCATGACGCCAGAGGATCTCGAGCTGTATCGTCTTCAGAAGGCCGGTATTAACACTGA
GGAAGACATGGGAGATGATCCGAACAAGCGACTGAAGACAAAGACAAACCCCACAA
CTCACATGTACACCCACTGCGAGATTCACCCAAGTATGATCTTAGGTATCTGTGCTAGTA
TCATTCCTTTCCCCGATCACAACCAGGTATGTTGTCCGCCCTTCCAGTCTTAATAACGAA
GAAAAATACTAATGG

>MG917685.1 *Trichoderma harzianum* T3
AGCTGTTCCGTGGTATCATGCGAAGGATGAACACTGAGTTGGCCAACTATCTGAGACG
TTGCGTTGAGGGTAACCGACACTTCAACCTGGCTGTGGGTATCAAGCCCGGCACACTT

TCAAACGGATTGAAGTATTCGCTTGCCACAGGAAACTGGGGTGATCAGAAGAAGGCC
ATGAGCTCAACTGCTGGTGTGTCCCAGGTGCTTAACCGTTACACATTTGCTTCGACCTT
GTCACATTTGCGTCGTACCAACACCCCTATCGGAAGAGATGGTAAGCTGGCAAAGCCT
CGACAGCTTCACAACACGCATTGGGGTTTGGTCTGCCCAGCCGAGACACCCGAAGGA
CAGGCTTGTGGTCTGGTCAAGAACTTGTCTTTGATGTGTTACGTCAGTGTCGGTTCTCC
CTCCGAACCTCTGATTGAGTTCATGATCAACAGAGGTATGGAAGTCGTCGAAGAGTAC
GAGCCGCTGCGGTATCCTCATGCTACGAAGATTTTCGTGAACGGTGTCTGGGTTGGAG
TCCACCAAGACCCTAAGCACTTGGTGAACCAGGTCCTGGACACTCGTCGCAAGTCCTA
TCTGCAATACGAAGTCTCTCTCGTGAGAGAAATTCGAGACCAGGAATTCAAAATCTTTT
CCGACGCAGGCCGTGTCATGCGACCAGTCTTTACCGTTCAGCAGGAAGATGACCCGGA
AACGGGCATCAACAAGGGCCACCTGGTATTGACCAAGGAGCTCGTCAATAGATTGGCC
AAGGAGCAGGCTGAGCCTCCGGAAGACCCCAGCATGAAGATTGGATGGGAGGGATTG
ATTAGGGCTGGTGCAGTTGAATATCTCGACGCCGAGGAAGAGGAGACGTCCATGATCT
GCATGACGCCAGAGGATCTCGAGCTGTATCGTCTTCAGAAGGCCGGTATTAACACTGA
GGAAGACATGGGAGATGATCCGAACAAGCGACTGAAGACCAAGACGAACCCGACAA
CTCACATGTACACCCATTGCGAGATTCACCCAAGTATGATCTTAGGTATCTGTGCTAGTA
TCATTCCTTTCCCCGATCACAACCAGGTATGTTGTCCGCCCTTCCAGTCTTATTAACGAA
GAAAAATACTAATGGTGTGCATAGTCCCCCGTAC

>MT641352.1 *Trichoderma harzianum* MT2
CTGGCCAAGCTGTTCCGTGGCATCATGCGAAGGATGAACACTGAGTTGGCCAACTACC
TGAGACGATGCGTTGAGGGCAACCGACACTTCAACCTTGCTGTTGGTATCAAGCCCGG
CACGCTTTCAAACGGATTGAAGTATTCGCTTGCCACAGGCAACTGGGGTGATCAGAAG
AAGGCCATGAGCTCAACTGCAGGTGTGTCCCAGGTGCTTAACCGATACACGTTTGCTT
CGACCCTGTCACATTTGCGTCGTACCAACACTCCCATCGGAAGAGATGGTAAGCTGGC
AAAGCCTCGACAGCTTCACAACACGCATTGGGGTTTGGTCTGCCCAGCCGAAACACC
CGAAGGACAGGCCTGTGGTCTGGTCAAGAACTTGTCTTTGATGTGTTACGTCAGTGTC
GGTTCTCCCTCTGAGCCTCTAATTGAGTTCATGATCAACAGAGGCATGGAAGTCGTCGA
AGAGTACGAGCCGCTGCGGTATCCTCATGCTACAAAGATTTTTGTGAACGGTGTCTGG
GTTGGAGTTCACCAAGACCCTAAGCACTTGGTGAACCAGGTCCTGGACACTCGTCGCA
AGTCCTATCTGCAGTACGAAGTCTCTCTTGTGAGAGAAATTCGAGACCAGGAATTCAA
AATCTTTTCCGACGCTGGTCGTGTCATGCGACCAGTCTTTACCGTTCAGCAGGAAGATG
ACCCGGAAACGGGCATCAACAAGGGCCACCTGGTATTGACCAAGGAACTCGTCAATA
GATTGGCCAAGGAGCAGGCTGAGCCTCCGGAAGACCCCAGCATGAAGATTGGATGGG
AGGGATTGATTAGGGCTGGTGCAGTTGAATATCTCGACGCCGAGGAAGAGGAAACGTC
CATGATCTGCATGACGCCAGAGGATCTCGAGCTGTATCGTCTTCAGAAGGCCGGTATTA
ACACTGAGGAAGACATGGGAGATGATCCGAACAAGCGACTGAAGACAAAGACAAAC
CCCACAACTCACATGTACACCCACTGCGAGATTCACCCAAGTATGATCTTAGGTATCTG
TGCTAGTATCATTCCTTTCCCCGATCACAACCAGGTATGTTGTCCGCCCTTCCAGTGTTA
ATAACGAAGAAAAATATAATGGT

>MG917683.1 *Trichoderma harzianum* T5
AGCTGTTCCGTGGTATCATGCGAAGGATGAACACTGAATTGGCCAACTATCTGAGACG
GTGCGTTGAGGGCAACCGACACTTCAACCTGGCTGTGGGTATCAAGCCCGGCACACTT

TCAAACGGATTGAAGTATTCGCTTGCCACAGGAAACTGGGGTGATCAGAAGAAGGCC
ATGAGCTCAACTGCTGGTGTGTCCCAGGTGCTTAACCGTTACACATTTGCTTCGACCTT
GTCACATTTGCGTCGTACCAACACCCCTATCGGAAGAGATGGTAAGCTGGCAAAGCCT
CGACAGCTTCACAACACGCATTGGGGTTTGGTCTGCCCAGCCGAAACACCCGAAGGA
CAGGCCTGTGGTCTGGTCAAGAACTTGTCTTTGATGTGTTACGTCAGTGTCGGTTCTCC
CTCCGAACCTCTGATTGAGTTCATGATCAACAGAGGTATGGAAGTCGTCGAAGAGTAC
GAGCCGCTGCGGTATCCTCATGCTACAAAGATTTTCGTGAACGGTGTCTGGGTTGGAG
TTCACCAAGACCCTAAGCACTTGGTGAACCAGGTTCTAGATACTCGTCGCAAGTCCTAT
CTGCAATACGAAGTCTCTCTCGTGAGAGAAATTCGAGACCAGGAATTCAAAATCTTTT
CCGACGCAGGCCGTGTCATGCGACCAGTCTTTACCGTTCAGCAGGAAGATGACCCGGA
AACGGGCATCAACAAGGGCCACCTGGTATTGACCAAGGAGCTCGTCAATAGGTTGGCC
AAGGAGCAGGCTGAGCCCCCGGAAGACCCCAGCATGAAGATTGGATGGGAGGGATTG
ATTAGGGCTGGTGCGGTCGAATATCTCGACGCCGAGGAAGAGGAGACGTCCATGATCT
GCATGACGCCAGAGGATCTCGAGCTGTATCGTCTTCAGAAGGCCGGTATTAACACCGA
GGAAGACATGGGAGATGATCCGAACAAGCGACTAAAGACCAAGACGAACCCGACAAC
TCATATGTACACCCACTGCGAGATTCACCCAAGTATGATCTTGGGTATCTGTGCCAGTAT
CATTCCTTTCCCCGATCACAACCAGGTATGTTGTCCACCCTTCCAGTCTTATGAACGAA
GAAAAATACTAATGGTGTACATATCC

>MZ603731.1 *Trichoderma harzianum* CGMCC 20739
TGGCGGGTCCCTTGCTGGCTAAGCTGTTCCGTGGTATCATGCGAAGGATGAACACTGA
ATTGGCCAACTATCTGAGACGGTGCGTTGAGGGCAACCGACACTTCAACCTGGCTGTG
GGTATCAAGCCCGGCACACTTTCAAACGGATTGAAGTATTCGCTTGCCACAGGAAACT
GGGGTGATCAGAAGAAGGCCATGAGCTCAACTGCTGGTGTGTCCCAGGTGCTTAACCG
TTACACATTTGCTTCGACCTTGTCACATTTGCGTCGTACCAACACCCCTATTGGAAGAG
ATGGTAAGCTGGCAAAGCCTCGACAGCTTCACAACACGCATTGGGGTTTGGTCTGCCC
AGCCGAGACACCCGAAGGACAGGCTTGTGGTCTGGTCAAGAACTTGTCTTTGATGTGT
TACGTCAGTGTCGGTTCTCCCTCTGAGCCTCTGATTGAGTTCATGATCAACAGAGGTAT
GGAAGTCGTTGAAGAGTATGAGCCGCTGCGGTATCCTCATGCTACAAAGATTTTCGTGA
ACGGTGTCTGGGTTGGAGTTCACCAAGACCCTAAGCACTTGGTGAACCAGGTCCTGG
ACACTCGTCGCAAGTCCTATCTGCAATACGAAGTCTCTCTCGTGAGAGAAATTCGAGA
CCAGGAATTCAAAATCTTTTCCGACGCAGGCCGTGTCATGCGACCAGTCTTTACCGTTC
AACAGGAAGATGACCCGGAAACGGGCATCAACAAGGGCCACCTGGTATTGACCAAGG
AGCTCGTCAATAGATTGGCCAAGGAGCAGGCTGAGCCTCCGGAAGACCCCAGCATGA
AGATTGGATGGGAGGGATTGATTAGGGCTGGTGCAGTTGAATATCTCGACGCCGAGGA
AGAGGAGACGTCCATGATCTGCATGACGCCAGAGGATCTCGAGCTGTATCGTCTTCAG
AAGGCCGGTATTAACACCGAAGAAGACATGGGAGATGATCCGAATAAGCGACTGAAG
ACCAAGACGAACCCGACAACTCACATGTACACCCATTGCGAGATTCACCCAAGTATGA
TCTTAGGTATCTGTGCTAGTATCATTCCTTTCCCCGATCACAACCAGGTATGTTGTCCAC
CCTTCCAGTCTTATTAATGAAGAAAAATACTAATGGTGTACATAGTCCCC

>MG873465.1 *Trichoderma harzianum* T8
AGCTGTTCCGTGGTATCATGCGAAGGATGAACACTGAATTGGCCAACTATCTGAGACG
GTGCGTTGAGGGCAACCGACACTTCAACCTGGCTGTGGGTATCAAGCCCGGCACACTT

TCAAACGGATTGAAGTATTCGCTTGCCACAGGAAACTGGGGTGATCAGAAGAAGGCC
ATGAGCTCAACTGCTGGTGTGTCCCAGGTGCTTAACCGTTACACATTTGCTTCGACCTT
GTCACATTTGCGTCGTACCAACACCCCTATCGGAAGAGATGGTAAGCTGGCAAAGCCT
CGACAGCTTCACAACACGCATTGGGGTTTGGTCTGCCCAGCCGAGACACCCGAAGGA
CAGGCTTGTGGTCTGGTCAAGAACTTGTCTTTGATGTGTTACGTCAGTGTCGGTTCTCC
CTCCGAACCTCTGATTGAGTTCATGATCAACAGAGGTATGGAAGTCGTCGAAGAGTAC
GAGCCTCTGCGGTATCCTCATGCTACAAAGATTTTTGTGAACGGTGTCTGGGTTGGAGT
TCACCAAGACCCTAAGCACTTGGTGAACCAGGTTCTAGATACTCGTCGCAAGTCCTATC
TGCAATACGAAGTCTCTCTCGTGAGAGAAATTCGAGACCAGGAATTTAAAATCTTTTCC
GACGCAGGCCGTGTCATGCGACCAGTCTTTACCGTTCAGCAGGAAGATGACCCGGAA
ACGGGCATCAACAAGGGCCACCTGGTATTGACCAAGGAGCTCGTCAATAGATTGGCCA
AGGAGCAGGCTGAGCCTCCGGAAGACCCCAGCATGAAGATTGGATGGGAGGGATTGA
TTAGGGCTGGTGCAGTTGAATATCTCGACGCCGAGGAAGAGGAGACGTCTATGATCTG
CATGACGCCAGAGGATCTCGAGCTGTATCGTCTTCAGAAGGCCGGTATTAACACTGAA
GAAGACATGGGAGATGATCCAAACAAGCGACTGAAGACCAAGACGAATCCGACAACT
CACATGTACACCCATTGCGAGATTCACCCAAGTATGATCTTAGGTATCTGTGCCAGTATC
ATTCCTTTCCCCGATCACAATCAGGTATGTTGTCCACCCTTCCAGACTTATCAACGAAG
AAAAATACTAATAGTGTATATAGTCCC

>MG873464.1 *Trichoderma harzianum* T7
AGCTGTTCCGTGGTATCATGCGAAGGATGAACACTGAATTGGCCAACTATCTGAGACG
GTGCGTTGAGGGCAACCGACACTTCAACCTGGCTGTGGGTATCAAGCCCGGCACACTT
TCAAACGGATTGAAGTATTCGCTTGCCACAGGAAACTGGGGTGATCAGAAGAAGGCC
ATGAGCTCAACTGCTGGTGTGTCCCAGGTGCTTAACCGTTACACATTTGCTTCGACCTT
GTCACATTTGCGTCGTACCAACACCCCTATCGGAAGAGATGGTAAGCTGGCAAAGCCT
CGACAGCTTCACAACACGCATTGGGGTTTGGTCTGCCCAGCCGAGACACCCGAAGGA
CAGGCTTGTGGTCTGGTCAAGAACTTGTCTTTGATGTGTTACGTCAGTGTCGGTTCTCC
CTCCGAACCTCTGATTGAGTTCATGATCAACAGAGGTATGGAAGTCGTCGAAGAGTAC
GAGCCTCTGCGGTATCCTCATGCTACAAAGATTTTTGTGAACGGTGTCTGGGTTGGAGT
TCACCAAGACCCTAAGCACTTGGTGAACCAGGTTCTAGATACTCGTCGCAAGTCCTATC
TGCAATACGAAGTCTCTCTCGTGAGAGAAATTCGAGACCAGGAATTTAAAATCTTTTCC
GACGCAGGCCGTGTCATGCGACCAGTCTTTACCGTTCAGCAGGAAGATGACCCGGAA
ACGGGCATCAACAAGGGCCACCTGGTATTGACCAAGGAGCTCGTCAATAGATTGGCCA
AGGAGCAGGCTGAGCCTCCGGAAGACCCCAGCATGAAGATTGGATGGGAGGGATTGA
TTAGGGCTGGTGCAGTTGAATATCTCGACGCCGAGGAAGAGGAGACGTCTATGATCTG
CATGACGCCAGAGGATCTCGAGCTGTATCGTCTTCAGAAGGCCGGTATTAACACTGAA
GAAGACATGGGAGATGATCCAAACAAGCGACTGAAGACCAAGACGAATCCGACAACT
CACATGTACACCCATTGCGAGATTCACCCAAGTATGATCTTAGGTATCTGTGCCAGTATC
ATTCCTTTCCCCGATCACAATCAGGTATGTTGTCCACCCTTCCAGACTTATCAACGAAG
AAAAATACTAATAGTGTATATAGTCCC

>XM_024924757.1 *Trichoderma harzianum* CBS 226.95
CCTGCTGGCAAAGCTGTTCCGTGGTATCATGCGAAGGATGAACACTGAGTTGGCCAAC
TATCTGAGACGATGCGTCGAGGGCAACCGACACTTCAACCTTGCTGTGGGTATCAAGC

CCGGCACGCTTTCAAACGGATTGAAGTATTCGCTTGCCACAGGAAACTGGGGTGATCA
GAAGAAGGCCATGAGCTCAACTGCAGGTGTGTCCCAGGTGCTTAACCGTTACACGTTT
GCTTCGACCCTATCACATTTGCGTCGTACCAATACTCCTATCGGAAGAGATGGTAAGCT
CGCAAAGCCTCGACAGCTTCACAACACGCACTGGGGTTTGGTCTGCCCGGCCGAGAC
ACCCGAGGGACAGGCTTGTGGTCTGGTCAAGAACTTGTCTTTGATGTGTTACGTCAGT
GTCGGTTCTCCCTCCGAACCTCTGATTGAGTTCATGATCAACAGAGGTATGGAAGTCGT
GGAAGAGTACGAGCCGCTGCGGTATCCTCATGCTACAAAGATTTTTGTGAACGGTGTC
TGGGTTGGAGTCCACCAAGACCCTAAGCACTTGGTGAACCAGGTCCTGGACACTCGTC
GCAAGTCCTATCTGCAATACGAAGTCTCTCTCGTGAGAGAAATTCGAGACCAGGAATT
CAAAATCTTTTCCGACGCAGGCCGTGTAATGCGGCCAGTCTTTACCGTTCAGCAGGAA
GATGACCCGGAAACGGGCATCAACAAGGGCCACCTGGTATTGACCAAGGAGCTCGTC
AATAGATTGGCCAAGGAGCAGGCTGAACCTCCGGAAGACCCCAGCATGAAGATTGGAT
GGGAGGGATTGATTAGGGCTGGTGCGGTTGAATATCTCGACGCCGAGGAAGAGGAGA
CGTCCATGATCTGCATGACGCCAGAGGATCTCGAGCTGTATCGTCTTCAGAAGGCTGGT
ATTAACACTGAGGAAGACATGGGAGATGACCCGAACAAGCGACTAAAGACCAAGACA
AACCCGACTACTCACATGTACACCCATTGCGAGATTCACCCAAGTATGATCTTAGGCAT
CTGTGCTAGTATCATTCCTTTCCCCGATCACAACCAG

>FJ442793.1 *Trichoderma lentiforme* DIS 218E
CCTGCTGGCCAAGCTGTTCCGTGGTATCATGCGAAGGATGAACACTGAATTGGCCAAC
TATCTGAGACGATGCGTTGAGGGTAACCGACACTTCAACCTTGCTGTTGGTATCAAGCC
CGGCACCCTTTCAAACGGATTGAAGTATTCGCTTGCCACAGGAAACTGGGGTGATCAG
AAGAAGGCCATGAGCTCAACTGCAGGCGTGTCCCAGGTGCTTAACCGTTACACGTTTG
CTTCGACCCTATCACATTTGCGTCGTACCAACACTCCTATCGGAAGAGATGGTAAGCTG
GCAAAGCCTCGACAGCTTCACAACACGCATTGGGGTTTGGTCTGCCCAGCCGAGACA
CCCGAAGGACAGGCCTGTGGTCTGGTCAAGAACTTGTCTTTGATGTGTTACGTCAGTG
TCGGTTCTCCCTCCGAACCTCTGATTGAGTTCATGATCAACAGAGGTATGGAAGTCGTT
GAAGAGTACGAGCCGCTGCGGTATCCTCATGCTACAAAGATTTTTGTGAACGGTGTCT
GGGTTGGAGTTCACCAAGACCCTAAGCACTTGGTGAACCAGGTCCTGGACACTCGTC
GCAAGTCCTATCTGCAGTACGAAGTCTCTCTCGTGAGAGAAATTCGAGACCAGGAATT
CAAAATCTTTTCCGACGCAGGTCGTGTCATGCGACCAGTCTTTACCGTTCAGCAGGAA
GATGATCCGGAAACGGGCATCAACAAGGGCCACCTGGTATTGACCAAGGAGCTCGTCA
ATAGATTGGCCAAGGAGCAGGCTGAGCCTCCGGAAGACCCCAGCATGAAGATTGGATG
GGAGGGATTGATTAGGGCTGGTGCAGTTGAATATCTCGACGCCGAAGAAGAGGAGAC
GTCCATGATCTGCATGACGCCAGAGGATCTCGAGCTGTATCGTCTTCAGAAGGCCGGTA
TTAACACTGAGGAAGACATGGGAGATGACCCGAACAAGCGACTAAAGACAAAGACGA
ATCCGACAACTCATATGTACACCCATTGCGAGATTCACCCAAGTATGATCTTAGGTATCT
GTGCTAGTA

>FJ442787.1 *Trichoderma lentiforme* DIS 173F
CCTGCTGGCCAAGCTGTTCCGTGGTATCATGCGAAGGATGAACACTGAATTGGCCAAC
TATCTGAGACGATGCGTTGAGGGTAACCGACACTTCAACCTTGCTGTTGGTATCAAGCC
CGGCACCCTTTCAAACGGATTGAAGTATTCGCTTGCCACAGGAAACTGGGGTGATCAG
AAGAAGGCCATGAGCTCAACTGCAGGCGTGTCCCAGGTGCTTAACCGTTACACGTTTG

CTTCGACCCTATCACATTTGCGTCGTACCAACACTCCTATCGGAAGAGATGGTAAGCTG
GCAAAGCCTCGACAGCTTCACAACACGCATTGGGGTTTGGTCTGCCCAGCCGAGACA
CCCGAAGGACAGGCCTGTGGTCTGGTCAAGAACTTGTCTTTGATGTGTTACGTCAGTG
TCGGTTCTCCCTCCGAACCTCTGATTGAGTTCATGATCAACAGAGGTATGGAAGTCGTT
GAAGAGTACGAGCCGCTGCGGTATCCTCATGCTACAAAGATTTTTGTGAACGGTGTCT
GGGTTGGAGTTCACCAAGACCCTAAGCACTTGGTGAACCAGGTCCTGGACACTCGTC
GCAAGTCCTATCTGCAGTACGAAGTCTCTCTCGTGAGAGAAATTCGAGACCAGGAATT
CAAAATCTTTTCCGACGCAGGTCGTGTCATGCGACCAGTCTTTACCGTTCAGCAGGAA
GATGATCCGGAAACGGGCATCAACAAGGGCCACCTGGTATTGACCAAGGAGCTCGTCA
ATAGATTGGCCAAGGAGCAGGCTGAGCCTCCGGAAGACCCCAGCATGAAGATTGGATG
GGAGGGATTGATTAGGGCTGGTGCAGTTGAATATCTCGACGCCGAAGAAGAGGAGAC
GTCCATGATCTGCATGACGCCAGAGGATCTCGAGCTGTATCGTCTTCAGAAGGCCGGTA
TTAACACTGAGGAAGACATGGGAGATGACCCGAACAAGCGACTAAAGACAAAGACGA
ATCCGACAACTCATATGTACACCCATTGCGAGATTCACCCAAGTATGATCTTAGGTATTT
GTGCTAGTA

>FJ442695.1 *Trichoderma lentiforme* DIS 246J
CCTGCTGGCCAAGCTGATCCGTGGTATCATGCGAAGGATGAACACTGAATTGGCCAAC
TATCTGAGACGATGCGTTGAGGGTAACCGACACTTCAACCTTGCTGTTGGTATCAAGCC
CGGCACCCTTTCAAACGGATTGAAGTATTCGCTTGCCACAGGAAACTGGGGTGATCAG
AAGAAGGCCATGAGCTCAACTGCAGGCGTGTCCCAGGTGCTTAACCGTTACACGTTTG
CTTCGACCCTATCACATTTGCGTCGTACCAACACTCCTATCGGAAGAGATGGTAAGCTG
GCAAAGCCTCGACAGCTTCACAACACGCATTGGGGTTTGGTCTGCCCAGCCGAGACA
CCCGAAGGACAGGCCTGTGGTCTGGTCAAGAACTTGTCTTTGATGTGTTACGTCAGTG
TCGGTTCTCCCTCCGAACCTCTGATTGAGTTCATGATCAACAGAGGTATGGAAGTCGTT
GAAGAGTACGAGCCGCTGCGGTATCCTCATGCTACAAAGATTTTTGTGAACGGTGTCT
GGGTTGGAGTTCACCAAGACCCTAAGCACTTGGTGAACCAGGTCCTGGACACTCGTC
GCAAGTCCTATCTGCAGTACGAAGTCTCTCTCGTGAGAGAAATTCGAGACCAGGAATT
CAAAATCTTTTCCGACGCAGGTCGTGTCATGCGACCAGTCTTTACCGTTCAGCAGGAA
GATGATCCGGAAACGGGCATCAACAAGGGCCACCTGGTATTGACCAAGGAGCTCGTCA
ATAGATTGGCCAAGGAGCAGGCTGAGCCTCCGGAAGACCCCAGCATGAAGATTGGATG
GGAGGGATTGATTAGGGCTGGTGCAGTTGAATATCTCGACGCCGAAGAAGAGGAGAC
GTCCATGATCTGCATGACGCCAGAGGATCTCGAGCTGTATCGTCTTCAGAAGGCCGGTA
TTAACACTGAGGAAGACATGGGAGATGACCCGAACAAGCGACTAAAGACAAAGACGA
ATCCGACAACTCATATGTACACCCATTGCGAGATTCACCCAAGTATGATCTTAGGTATCT
GTGCTAGTA

>FJ442707.1 *Trichoderma lentiforme* DIS 169C
CCTGCTGGCCAAGTTGTTCCGTGGTATCATGCGAAGGATGAACACTGAATTGGCCAAC
TATCTGAGACGATGCGTTGAGGGTAACCGACACTTCAACCTTGCTGTTGGTATCAAGCC
CGGCACCCTTTCAAACGGATTGAAGTATTCGCTTGCCACAGGAAACTGGGGTGATCAG
AAGAAGGCCATGAGCTCAACTGCAGGCGTGTCCCAGGTGCTTAACCGTTACACGTTTG
CTTCGACCCTATCACATTTGCGTCGTACCAACACTCCTATCGGAAGAGATGGTAAGCTG
GCAAAGCCTCGACAGCTTCACAACACGCATTGGGGTTTGGTCTGCCCAGCCGAGACA

CCCGAAGGACAGGCCTGTGGTCTGGTCAAGAACTTGTCTTTGATGTGTTACGTCAGTG
TCGGTTCTCCCTCCGAACCTCTGATTGAGTTCATGATCAACAGAGGTATGGAAGTCGTT
GAAGAGTACGAGCCGCTGCGGTATCCTCATGCTACAAAGATTTTTGTGAACGGTGTCT
GGGTTGGAGTTCACCAAGACCCTAAGCACTTGGTGAACCAGGTCCTGGACACTCGTC
GCAAGTCCTATCTGCAGTACGAAGTCTCTCTCGTGAGAGAAATTCGAGACCAGGAATT
CAAAATCTTTTCCGACGCAGGTCGTGTCATGCGACCAGTCTTTACCGTTCAGCAGGAA
GATGATCCGGAAACGGGCATCAACAAGGGCCACCTGGTATTGACCAAGGAGCTCGTCA
ATAGATTGGCCAAGGAGCAGGCTGAGCCTCCGGAAGACCCCAGCATGAAGATTGGATG
GGAGGGATTGATTAGGGCTGGTGCAGTTGAATATCTCGACGCCGAAGAAGAGGAGAC
GTCCATGATCTGCATGACGCCAGAGGATCTCGAGCTGTATCGTCTTCAGAAGGCCGGTA
TTAACACTGAGGAAGACATGGGAGATGACCCGAACAAGCGACTAAAGACAAAGACGA
ATCCGACAACTCATATGTACACCCATTGCGAGATTCACCCAAGTATGATCTTAGGTATTT
GTGCTAGTA

>FJ442692.1 *Trichoderma lentiforme* DIS 67B
CCTGCTGGCCAAGCTGTTCCGTGGTATCATGCGAAGGATGAACACTGAATTGGCCAAC
TATCTGAGACGATGCGTTGAGGGTAACCGACACTTCAACCTTGCTGTTGGTATCAAGCC
CGGCACCCTTTCAAACGGATTGAAGTATTCGCTTGCCACAGGAAACTGGGGTGATCAG
AAGAAGGCCATGAGCTCAACTGCAGGCGTGTCCCAGGTGCTTAACCGTTACACGTTTG
CTTCGACCCTATCACATTTGCGTCGTACCAACACTCCTATCGGAAGAGATGGTAAGCTG
GCAAAGCCTCGACAGCTTCACAACACGCATTGGGGTTTGGTCTGCCCAGCCGAGACA
CCCGAAGGACAGGCCTGTGGTCTGGTCAAGAACTTGTCTTTGATGTGTTACGTCAGTG
TCGGTTCTCCCTCCGAACCTCTGATTGAGTTCATGATCAACAGAGGTATGGAAGTCGTT
GAAGAGTACGAGCCGCTGCGGTATCCTCATGCTACAAAGATTTTTGTGAACGGTGTCT
GGGTTGGAGTTCACCAAGACCCTAAGCACTTGGTGAACCAGGTCCTGGACACTCGTC
GCAAGTCCTATCTGCAATACGAAGTCTCTCTCGTGAGAGAAATTCGAGACCAGGAATT
CAAAATCTTTTCCGACGCAGGTCGTGTCATGCGACCAGTCTTTACCGTTCAGCAGGAA
GATGATCCGGAAACGGGCATCAACAAGGGCCACCTGGTATTGACCAAGGAGCTCGTCA
ATAGATTGGCCAAGGAGCAAGCTGAACCTCCGGAAGACCCCAGCATGAAGATCGGAT
GGGAGGGACTGATTAGGGCTGGTGCGGTTGAATATCTCGACGCCGAGGAAGAGGAGA
CGTCCATGATCTGCATGACGCCTGAGGATCTCGAGCTGTATCGCCTTCAGAAGGCCGGT
ATTAACACTGAGGAAGACATGGGAGATGATCCGAACAAGCGACTAAAGACGAAGACG
AACCCGACAACTCATATGTACACCCACTGCGAGATTCACCCAAGTATGATCTTAGGTAT
CTGTGCTAGTA

>FJ442758.1 *Trichoderma lentiforme* DIS 246K
CCTGCTGGCCAAGCTGTTCCGTGGTATCATGCGAAGGATGAACACTGAATTGGCCAAC
TATCTGAGACGATGCGTTGAGGGTAACCGACACTTCAACCTTGCTGTTGGTATCAAGCC
CGGCACCCTTTCAAACGGATTGAAGTATTCGCTTGCCACAGGAAACTGGGGTGATCAG
AAGAAGGCCATGAGCTCAACTGCAGGCGTGTCCCAGGTGCTTAACCGTTACACGTTTG
CTTCGACCCTATCACATTTGCGTCGTACCAACACTCCTATCGGAAGAGATGGTAAGCTG
GCAAAGCCTCGACAGCTTCACAACACGCATTGGGGTTTGGTCTGCCCAGCCGAGACA
CCCGAAGGACAGGCCTGTGGTCTGGTCAAGAACTTGTCTTTGATGTGTTACGTCAGTG
TCGGTTCTCCCTCCGAACCTCTGATTGAGTTCATGATCAACAGAGGTATGGAAGTCGTT

GAAGAGTACGAGCCGCTGCGGTATCCTCATGCTACAAAGATTTTTGTGAACGGTGTCT
GGGTTGGAGTTCACCAAGACCCTAAGCACTTGGTGAACCAGGTCCTGGACACTCGTC
GCAAGTCCTATCTGCAGTACGAAGTCTCTCTCGTGAGAGAAATTCGAGACCAGGAATT
CAAAATCTTTTCCGACGCAGGTCGTGTCATGCGACCAGTCTTTACCGTTCAGCAGGAA
GATGATCCGGAAACGGGCATCAACAAGGGCCACCTGGTATTGACCAAGGAGCTCGTCA
ATAGATTGGCCAAGGAGCAGGCTGAGCCTCCGGAAGACCCCAGCATGAAGATTGGATG
GGAGGGATTGATTAGGGCTGGTGCAGTTGAATATCTCGACGCCGAAGAAGAGGAGAC
GTCCATGATCTGCATGACGCCAGAGGATCTCGAGCTGTATCGTCTTCAGAAGGCCGGTA
TTAACACTGAGGAAGACATGGGAGATGACCCGAACAAGCGACTAAAGACAAAGACGA
ATCCGACAACTCATATGCACACCCATTGCGAGATTCACCCAAGTATGATCTTAGGTATCT
BTKCTAGTA

>FJ442689.1 *Trichoderma lentiforme* DIS 167C
CCTGCTGGCCAAGTTGTTCCGTGGTATAATGCGAAGGATGAACACTGAGTTGGCCAAC
TACCTGAGACGGTGTGTTGAGGGTAACCGACACTTCAACCTTGCTGTTGGTATCAAGC
CCGGCACGCTCTCAAACGGATTGAAGTATTCGCTTGCCACAGGAAACTGGGGTGATCA
GAAGAAGGCCATGAGCTCGACTGCAGGTGTGTCACAGGTGCTTAACCGTTACACGTTT
GCTTCGACCTTGTCACATTTGCGTCGTACCAATACTCCTATCGGAAGAGATGGTAAGCT
GGCAAAGCCTCGACAGCTTCACAACACGCATTGGGGTTTGGTCTGTCCTGCCGAGACA
CCCGAAGGACAGGCCTGTGGTCTGGTCAAGAACTTGTCTTTGATGTGTTACGTCAGTG
TCGGTTCTCCCTCCGAGCCTCTGATTGAATTCATGATCAACAGAGGTATGGAGGTCGTC
GAAGAGTATGAGCCGCTGCGGTATCCTCATGCTACAAAGATTTTTGTGAACGGTGTCTG
GGTTGGAGTTCACCAAGACCCTAAGCACTTGGTGAACCAGGTTCTGGATACTCGTCGC
AAGTCCTATCTGCAATACGAAGTCTCTCTCGTGAGAGAAATTCGAGACCAGGAATTCA
AAATCTTTTCCGACGCAGGCCGTGTCATGCGACCAGTCTTTACCGTTCAGCAGGAAGA
TGACCCGGAAACGGGCATCAACAAGGGCCACCTGGTTTTGACCAAGGAGCTCGTCAA
TAGATTGGCCAAGGAGCAAGCTGAGCCTCCGGAAGACCCCAGCATGAAGATCGGATG
GGAGGGACTGATTAGGGCTGGTGCGGTTGAATATCTCGACGCCGAGGAAGAGGAGAC
GTCCATGATCTGCATGACGCCTGAGGATCTCGAGCTGTATCGCCTTCAGAAGGCCGGTA
TCAACACTGAGGAAGACATGGGAGATGATCCGAACAAGCGACTAAAGACGAAGACGA
ACCCGACAACTCATATGTACACCCACTGCGAGATTCACCCAAGTATGATCTTAGGTATC
TGTGCTAGTA

>FJ442778.1 *Trichoderma afarasin* DIS 314F
CCTGCTGGCCAAGCTGTTCCGTGGTATCATGCGAAGGATGAACACTGAATTGGCCAAC
TACCTGAGACGGTGTGTTGAGGGTAACCGACACTTCAACCTTGCTGTTGGTATCAAGC
CCGGCACGCTATCAAACGGATTGAAGTATTCGCTTGCCACAGGAAACTGGGGTGATCA
GAAGAAGGCCATGAGCTCGACTGCAGGTGTGTCACAGGTGCTTAACCGTTACACGTTT
GCTTCGACCTTGTCACATTTGCGTCGTACCAATACTCCTATCGGAAGAGATGGTAAGCT
GGCAAAGCCTCGACAGCTTCACAACACGCATTGGGGTTTGGTCTGTCCTGCCGAGACA
CCCGAAGGACAGGCCTGTGGTCTGGTCAAGAACTTGTCTTTGATGTGTTACGTCAGTG
TCGGTTCTCCCTCCGAGCCTCTGATTGAATTCATGATCAACAGAGGTATGGAGGTCGTC
GAAGAGTATGAGCCGCTGCGGTATCCTCATGCTACAAAGATTTTTGTGAACGGTGTCTG
GGTTGGAGTTCACCAAGACCCTAAGCACTTGGTGAACCAGGTTCTGGATACTCGTCGC

AAGTCCTATCTGCAATACGAAGTCTCTCTCGTGAGAGAAATTCGAGACCAGGAATTCA
AAATCTTTTCCGACGCAGGCCGTGTCATGCGACCAGTCTTTACCGTTCAGCAGGAAGA
TGACCCGGAAACGGGCATCAACAAGGGCCACCTGGTTTTGACCAAGGAGCTCGTCAA
TAGATTGGCCAAGGAGCAAGCTGAACCTCCGGAAGACCCTAGCATGAAGATCGGATG
GGAGGGACTGATTAGGGCTGGTGCGGTTGAATATCTCGACGCCGAGGAAGAGGAGAC
GTCCATGATCTGCATGACGCCTGAGGATCTCGAGCTGTATCGCCTTCAGAAGGCCGGTA
TTAACACTGAGGAAGACATGGGAGATGATCCGAACAAGCGACTAAAGACGAAGACGA
ACCCGACAACTCATATGTACACCCACTGCGAGATTCACCCAAGCATGATCTTAGGTATC
TGTGCTAGTA

>OP102132.1 *Trichoderma afroharzianum* Tri-1
CCTGCTGGCCAAGCTGTTCCGTGGTATCATGCGAAGGATGAACACTGAGTTGGCCAAC
TACCTGAGACGATGCGTTGAGGGCAACCGACACTTCAACCTGGCTGTTGGTATCAAGC
CCGGCACGCTTTCAAACGGACTGAAGTATTCGCTTGCCACAGGAAACTGGGGTGATCA
GAAGAAGGCCATGAGCTCAACTGCCGGTGTGTCCCAGGTGCTTAACCGTTACACGTTT
GCTTCGACCTTGTCACATTTGCGTCGTACCAACACTCCTATCGGGAGAGATGGTAAGCT
GGCGAAGCCTCGACAGCTTCACAACACGCATTGGGGCTTGGTCTGCCCAGCCGAGAC
ACCCGAAGGACAGGCCTGTGGGCTGGTCAAGAACTTGTCTTTGATGTGTTACGTCAGT
GTCGGTTCTCCCTCCGAGCCTTTGATTGAGTTCATGATCAACAGAGGTATGGAAGTCGT
CGAAGAGTACGAGCCGCTGCGGTATCCTCATGCTACAAAGATTTTTGTGAACGGTGTCT
GGGTTGGAGTTCACCAAGACCCTAAGCACTTGGTGAACCAGGTTCTGGATACTCGTCG
CAAGTCCTATCTGCAATACGAAGTCTCTCTCGTGAGAGAAATTCGAGACCAGGAATTC
AAAATCTTTTCCGATGCAGGTCGTGTCATGCGACCAGTCTTTACCGTTCAGCAGGAAG
ATGATCCGGAAACGGGCATCAACAAGGGCCACCTGGTATTGACCAAGGAGCTCGTCAA
TAGATTGGCCAAGGAGCAGGCTGAGCCTCCGGAAGACCCCAGCATGAAGATTGGATG
GGAGGGATTGATCAGGGCTGGTGCGGTTGAATATCTCGACGCCGAGGAAGAGGAGAC
GGCCATGATCTGCATGACACCAGAGGATCTCGAGCTGTATCGTCTTCAGAAGGCCGGT
ATCAACACTGAGGAAGACATGGGAGATGATCCGAACAAGCGACTCAAGACCAAGACG
AACCCGACAACTCACATGTACACCCATTGCGAGATTCACCCAAGTATGATCTTAGGTAT
CTGTGCTAGTATCATTCCTTTCCCCGATCACAACCAGGTATGTTGTCCACGCTTCCAGTC
TTATGAACGAAGAAAAAAACTAATGGTGTGCATAGTCCCC

>OP374186.1 *Trichoderma afroharzianum* Tr153
CTGCTGGCCAAGCTGTTCCGTGGTATCATGCGAAGGATGAACACTGAGTTGGCCAACT
ACCTGAGACGATGCGTTGAGGGCAACCGACACTTCAACCTGGCTGTTGGTATCAAGCC
CGGCACGCTTTCAAACGGACTGAAGTATTCGCTTGCCACAGGAAACTGGGGTGATCAG
AAGAAGGCCATGAGCTCAACTGCCGGTGTGTCCCAGGTGCTTAACCGTTACACGTTTG
CTTCGACCTTGTCACATTTGCGTCGTACCAACACTCCTATCGGGAGAGATGGTAAGCTG
GCGAAGCCTCGACAGCTTCACAACACGCATTGGGGCTTGGTCTGCCCAGCCGAGACA
CCCGAAGGACAGGCCTGTGGTCTGGTCAAGAACTTGTCTTTGATGTGTTACGTCAGTG
TCGGTTCTCCCTCCGAGCCTCTGATTGAGTTCATGATCAACAGAGGTATGGAAGTCGTC
GAAGAGTACGAGCCGCTGCGGTATCCTCATGCTACAAAGATTTTTGTGAACGGTGTCT
GGGTTGGAGTTCACCAAGACCCTAAGCACTTGGTGAACCAGGTTCTGGATACTCGTCG
CAAGTCCTATCTGCAATACGAAGTCTCTCTCGTGAGAGAAATTCGAGACCAGGAATTC

AAAATCTTTTCCGATGCAGGTCGTGTCATGCGACCAGTCTTTACCGTTCAGCAGGAAG
ATGATCCGGAAACGGGCATCAACAAGGGCCACCTGGTATTGACCAAGGAGCTCGTCAA
TAGATTGGCCAAGGAGCAGGCTGAGCCTCCGGAAGACCCCAGCATGAAGATTGGATG
GGAGGGATTGATCAGGGCTGGTGCGGTTGAATATCTCGACGCCGAGGAAGAGGAGAC
GGCCATGATCTGCATGACACCAGAGGATCTCGAGCTGTATCGTCTTCAGAAGGCCGGT
ATCAACACTGAGGAAGACATGGGAGATGATCCGAACAAGCGACTCAAGACCAAGACG
AACCCGACAACTCACATGTACACCCATTGCGAGATTCACCCAAGTATGATCTTAGGTAT
CTGTGCTAGTATCATTCCTTTCCCCGATCACAACCAGGTATGTTGTCCACGCTTCCAGTC
TTATGAACGAAGAAAAAACTAATGG

>OP374185.1 *Trichoderma afroharzianum* Tr138B
CTGCTGGCCAAGCTGTTCCGTGGTATCATGCGAAGGATGAACACTGAGTTGGCCAACT
ACCTGAGACGATGCGTTGAGGGCAACCGACACTTCAACCTGGCTGTTGGTATCAAGCC
CGGCACGCTTTCAAACGGACTGAAGTATTCGCTTGCCACAGGAAACTGGGGTGATCAG
AAGAAGGCCATGAGCTCAACTGCCGGTGTGTCCCAGGTGCTTAACCGTTACACGTTTG
CTTCGACCTTGTCACATTTGCGTCGTACCAACACTCCTATCGGGAGAGATGGTAAGCTG
GCGAAGCCTCGACAGCTTCACAACACGCATTGGGGCTTGGTCTGCCCAGCCGAGACA
CCCGAAGGACAGGCCTGTGGTCTGGTCAAGAACTTGTCTTTGATGTGTTACGTCAGTG
TCGGTTCTCCCTCCGAGCCTCTGATTGAGTTCATGATCAACAGAGGTATGGAAGTCGTC
GAAGAGTACGAGCCGCTGCGGTATCCTCATGCTACAAAGATTTTTGTGAACGGTGTCT
GGGTTGGAGTTCACCAAGACCCTAAGCACTTGGTGAACCAGGTTCTGGATACTCGTCG
CAAGTCCTATCTGCAATACGAAGTCTCTCTCGTGAGAGAAATTCGAGACCAGGAATTC
AAAATCTTTTCCGATGCAGGTCGTGTCATGCGACCAGTCTTTACCGTTCAGCAGGAAG
ATGATCCGGAAACGGGCATCAACAAGGGCCACCTGGTATTGACCAAGGAGCTCGTCAA
TAGATTGGCCAAGGAGCAGGCTGAGCCTCCGGAAGACCCCAGCATGAAGATTGGATG
GGAGGGATTGATCAGGGCTGGTGCGGTTGAATATCTCGACGCCGAGGAAGAGGAGAC
GGCCATGATCTGCATGACACCAGAGGATCTCGAGCTGTATCGTCTTCAGAAGGCCGGT
ATCAACACTGAGGAAGACATGGGAGATGATCCGAACAAGCGACTCAAGACCAAGACG
AACCCGACAACTCACATGTACACCCATTGCGAGATTCACCCAAGTATGATCTTAGGTAT
CTGTGCTAGTATCATTCCTTTCCCCGATCACAACCAGGTATGTTGTCCACGCTTCCAGTC
TTATGAACGAAGAAAAAACTAATGG

>OP374184.1 *Trichoderma afroharzianum* Tr132
CTGCTGGCCAAGCTGTTCCGTGGTATCATGCGAAGGATGAACACTGAGTTGGCCAACT
ACCTGAGACGATGCGTTGAGGGCAACCGACACTTCAACCTGGCTGTTGGTATCAAGCC
CGGCACGCTTTCAAACGGACTGAAGTATTCGCTTGCCACAGGAAACTGGGGTGATCAG
AAGAAGGCCATGAGCTCAACTGCCGGTGTGTCCCAGGTGCTTAACCGTTACACGTTTG
CTTCGACCTTGTCACATTTGCGTCGTACCAACACTCCTATCGGGAGAGATGGTAAGCTG
GCGAAGCCTCGACAGCTTCACAACACGCATTGGGGCTTGGTCTGCCCAGCCGAGACA
CCCGAAGGACAGGCCTGTGGTCTGGTCAAGAACTTGTCTTTGATGTGTTACGTCAGTG
TCGGTTCTCCCTCCGAGCCTCTGATTGAGTTCATGATCAACAGAGGTATGGAAGTCGTC
GAAGAGTACGAGCCGCTGCGGTATCCTCATGCTACAAAGATTTTTGTGAACGGTGTCT
GGGTTGGAGTTCACCAAGACCCTAAGCACTTGGTGAACCAGGTTCTGGATACTCGTCG
CAAGTCCTATCTGCAATACGAAGTCTCTCTCGTGAGAGAAATTCGAGACCAGGAATTC

AAAATCTTTTCCGATGCAGGTCGTGTCATGCGACCAGTCTTTACCGTTCAGCAGGAAG
ATGATCCGGAAACGGGCATCAACAAGGGCCACCTGGTATTGACCAAGGAGCTCGTCAA
TAGATTGGCCAAGGAGCAGGCTGAGCCTCCGGAAGACCCCAGCATGAAGATTGGATG
GGAGGGATTGATCAGGGCTGGTGCGGTTGAATATCTCGACGCCGAGGAAGAGGAGAC
GGCCATGATCTGCATGACACCAGAGGATCTCGAGCTGTATCGTCTTCAGAAGGCCGGT
ATCAACACTGAGGAAGACATGGGAGATGATCCGAACAAGCGACTCAAGACCAAGACG
AACCCGACAACTCACATGTACACCCATTGCGAGATTCACCCAAGTATGATCTTAGGTAT
CTGTGCTAGTATCATTCCTTTCCCCGATCACAACCAGGTATGTTGTCCACGCTTCCAGTC
TTATGAACGAAGAAAAAACTAATGG

>FJ442709.1 *Trichoderma afroharzianum* GJS 04-193
CCTGCTGGCCAAGCTGTTCCGTGGTATCATGCGAAGGATGAACACTGAGTTGGCCAAC
TACCTGAGACGATGCGTTGAGGGCAACCGACACTTCAACCTGGCTGTTGGTATCAAGC
CCGGCACGCTTTCAAACGGACTGAAGTATTCGCTTGCCACAGGAAACTGGGGTGATCA
GAAGAAGGCCATGAGCTCAACTGCCGGTGTGTCCCAGGTGCTTAACCGTTACACGTTT
GCTTCGACCTTGTCACATTTGCGTCGTACCAACACTCCTATCGGGAGAGATGGTAAGCT
GGCGAAGCCTCGACAGCTTCACAACACGCATTGGGGCTTGGTCTGCCCAGCCGAGAC
ACCCGAAGGACAGGCCTGTGGTCTGGTCAAGAACTTGTCTTTGATGTGTTACGTCAGT
GTCGGTTCTCCCTCCGAGCCTCTGATTGAGTTCATGATCAACAGAGGTATGGAAGTCGT
CGAAGAGTACGAGCCGCTGCGGTATCCTCATGCTACAAAGATTTTTGTGAACGGTGTCT
GGGTTGGAGTTCACCAAGACCCTAAGCACTTGGTGAACCAGGTTCTGGATACTCGTCG
CAAGTCCTATCTGCAATACGAAGTCTCTCTCGTGAGAGAAATTCGAGACCAGGAATTC
AAAATCTTTTCCGATGCAGGTCGTGTCATGCGACCAGTCTTTACCGTTCAGCAGGAAG
ATGATCCGGAAACGGGCATCAACAAGGGCCACCTGGTATTGACCAAGGAGCTCGTCAA
TAGATTGGCCAAGGAGCAGGCTGAGCCTCCGGAAGACCCCAGCATGAAGATTGGATG
GGAGGGATTGATCAGGGCTGGTGCGGTTGAATATCTCGACGCCGAGGAAGAGGAGAC
GGCCATGATCTGCATGACACCAGAGGATCTCGAGCTGTATCGTCTTCAGAAGGCCGGT
ATCAACACTGAGGAAGACATGGGAGATGATCCGAACAAGCGACTCAAGACCAAGACG
AACCCGACAACTCACATGTACACCCATTGCGAGATTCACCCAAGTATGATCTTAGGTAT
CTGTGCTAGTA

>FJ442691.1 *Trichoderma afroharzianum* GJS 04-186
CCTGCTGGCCAAGCTGTTCCGTGGTATCATGCGAAGGATGAACACTGAGTTGGCCAAC
TACCTGAGACGATGCGTTGAGGGCAACCGACACTTCAACCTGGCTGTTGGTATCAAGC
CCGGCACGCTTTCAAACGGACTGAAGTATTCGCTTGCCACAGGAAACTGGGGTGATCA
GAAGAAGGCCATGAGCTCAACTGCCGGTGTGTCCCAGGTGCTTAACCGTTACACGTTT
GCTTCGACCTTGTCACATTTGCGTCGTACCAACACTCCTATCGGGAGAGATGGTAAGCT
AGCGAAGCCTCGACAGCTTCACAACACGCATTGGGGCTTGGTCTGCCCAGCCGAGAC
ACCCGAAGGACAGGCCTGTGGTCTGGTCAAGAACTTGTCTTTGATGTGTTACGTCAGT
GTCGGTTCTCCCTCCGAGCCTCTGATTGAGTTCATGATCAACAGAGGTATGGAAGTCGT
CGAAGAGTACGAGCCGCTGCGGTATCCTCATGCTACAAAGATTTTTGTGAACGGTGTCT
GGGTTGGAGTTCACCAAGACCCTAAGCACTTGGTGAACCAGGTTCTGGATACTCGTCG
CAAGTCCTATCTGCAATACGAAGTCTCTCTCGTGAGAGAAATTCGAGACCAGGAATTC
AAAATCTTTTCCGATGCAGGTCGTGTCATGCGACCAGTCTTTACCGTTCAGCAGGAAG

ATGATCCGGAAACGGGCATCAACAAGGGCCACCTGGTATTGACCAAGGAGCTCGTCAA
TAGATTGGCCAAGGAGCAGGCTGAGCCTCCGGAAGACCCCAGCATGAAGATTGGATG
GGAGGGATTGATCAGGGCTGGTGCGGTTGAATATCTCGACGCCGAGGAAGAGGAGAC
GGCCATGATCTGCATGACACCAGAGGATCTCGAGCTGTATCGTCTTCAGAAGGCCGGT
ATCAACACTGAGGAAGACATGGGAGATGATCCGAACAAGCGACTCAAGACCAAGACG
AACCCGACAACTCACATGTACACCCATTGCGAGATTCACCCAAGTATGATCTTAGGTAT
CTGTGCTAGTA

>FJ442726.1 *Trichoderma afroharzianum* GJS 00-24
CCTGCTGGCTAAGCTGTTCCGTGGTATCATGCGAAGGATGAACACTGAGTTGGCCAAC
TACCTGAGACGATGCGTTGAGGGCAACCGACACTTCAACCTGGCTGTTGGTATCAAGC
CCGGCACGCTTTCAAACGGACTGAAGTATTCGCTTGCCACAGGAAACTGGGGTGATCA
GAAGAAGGCCATGAGCTCAACTGCCGGTGTGTCCCAGGTGCTTAACCGTTACACGTTT
GCTTCGACCTTGTCACATTTGCGTCGTACCAACACTCCTATCGGGAGAGATGGTAAGCT
AGCGAAGCCTCGACAGCTTCACAACACGCATTGGGGCTTGGTCTGCCCAGCCGAGAC
ACCCGAAGGACAGGCCTGTGGTCTGGTCAAGAACTTGTCTTTGATGTGTTACGTCAGT
GTCGGTTCTCCCTCCGAGCCTCTGATTGAGTTCATGATCAACAGAGGTATGGAAGTCGT
CGAAGAGTACGAGCCGCTGCGGTATACTCATGCTACAAAGATTTTTGTGAACGGTGTCT
GGGTTGGAGTTCACCAAGACCCTAAGCACTTGGTGAACCAGGTTCTGGATACTCGTCG
CAAGTCCTATCTGCAATACGAAGTCTCTCTCGTGAGAGAAATTCGAGACCAGGAATTC
AAAATCTTTTCCGATGCAGGTCGTGTCATGCGACCAGTCTTTACCGTTCAGCAGGAAG
ATGATCCGGAAACGGGCATCAACAAGGGCCACCTGGTATTGACCAAGGAGCTCGTCAA
TAGATTGGCCAAGGAGCAGGCTGAGCCTCCGGAAGACCCCAGCATGAAGATTGGATG
GGAGGGATTGATCAGGGCTGGTGCGGTTGAATATCTCGACGCCGAGGAAGAGGAGAC
GGCCATGATCTGCATGACACCAGAGGATCTCGAGCTGTATCGTCTTCAGAAGGCCGGT
ATCAACACTGAGGAAGACATGGGAGATGATCCGAACAAGCGACTCAAGACCAAGACG
AACCCGACAACTCACATGTACACCCATTGCGAGATTCACCCAAGTATGATCTTAGGTAT
CTGTGCTAGTA

>KX632572.1 *Trichoderma atrobrunneum* T42
CCTGCTGGCCAAGCTGTTCCGTGGTATCATGCGAAGGATGAACACTGAATTGGCCAAC
TATCTGAGACGGTGCGTTGAGGGCAACCGACACTTCAACCTGGCTGTGGGTATCAAGC
CCGGCACGCTTTCAAACGGATTGAAGTATTCGCTTGCCACAGGAAACTGGGGTGATCA
GAAGAAGGCCATGAGCTCAACTGCAGGCGTGTCCCAGGTGCTTAACCGATACACGTTT
GCTTCGACCCTGTCACATTTGCGTCGTACCAACACCCCTATCGGAAGAGATGGTAAGCT
GGCAAAGCCTCGACAGCTTCACAACACGCATTGGGGTTTGGTCTGCCCAGCCGAGAC
ACCCGAAGGACAGGCCTGTGGTCTGGTCAAGAACTTGTCTTTGATGTGTTACGTCAGT
GTCGGTTCTCCCTCTGAGCCTCTGATTGAGTTCATGATCAACAGAGGTATGGAAGTTGT
CGAAGAGTACGAGCCGCTGAGGTATCCTCATGCTACAAAGATTTTTGTGAACGGTGTC
TGGGTTGGAGTTCACCAAGACCCTAAGCACTTGGTGAACCAGGTTCTGGATACTCGTC
GCAAGTCCTATCTGCAATACGAAGTCTCTCTCGTGAGAGAAATTCGAGACCAGGAATT
TAAAATCTTTTCCGACGCAGGTCGTGTCATGCGACCAGTCTTTACCGTTCAACAGGAA
GATGACCCGGAAACGGGCATCAACAAGGGCCACCTGGTATTGACCAAGGAGCTCGTC
AATAGATTGGCCAAGGAGCAGGCTGAGCCTCCGGAAGACCCCAGCATGAAGATTGGAT

GGGAGGGATTGATCAGGGCTGGTGCGGTTGAATATCTCGACGCCGAGGAAGAGGAGA
CGTCCATGATCTGCATGACGCCAGAGGATCTCGAGCTGTATCGTCTTCAGAAGGCCGGT
ATTAACACTGAGGAAGACATGGGAGATGACCCGAACAAGCGACTAAAGACCAAGACG
AACCCGACAACTCACATGTACACCCATTGCGAGATTCACCCAAGTATGATCTTAGGTAT
CTGTGCTAGTATCATTCCTTTCCCCGATCACAACCAGGTATGTTGTCCACCCTCTCAGTC
TTAT

>KX632573.1 *Trichoderma atrobrunneum* T57
CCTGCTGGCCAAGCTGTTCCGTGGTATCATGCGAAGGATGAACACTGAATTGGCCAAC
TATCTGAGACGATGCGTTGAGGGTAACCGACACTTCAACCTTGCTGTTGGTATCAAGCC
CGGCACGCTCTCCAACGGATTGAAGTATTCGCTTGCCACAGGAAACTGGGGTGATCAG
AAGAAGGCCATGAGCTCAACTGCAGGTGTGTCCCAGGTGCTTAACCGTTACACGTTTG
CTTCGACCCTATCACATTTGCGTCGTACCAATACTCCTATCGGAAGAGATGGTAAGCTG
GCAAAGCCTCGTCAGCTTCACAACACGCATTGGGGTTTGGTCTGCCCAGCCGAGACAC
CCGAAGGACAGGCCTGTGGTCTGGTCAAGAACTTGTCTTTGATGTGTTACGTCAGTGT
CGGTTCTCCCTCCGAACCTCTGATTGAGTTCATGATCAACAGAGGTATGGAAGTTGTCG
AAGAATACGAGCCGCTGCGCTATCCTCATGCTACAAAGATTTTTGTGAACGGTGTCTGG
GTTGGAGTTCATCAAGACCCTAAGCACTTGGTGAACCAGGTTCTAGATACTCGTCGCA
AGTCCTATCTGCAATACGAAGTCTCTCTCGTGAGAGAAATTCGAGACCAGGAATTCAA
AATCTTTTCCGACGCAGGCCGTGTCATGAGACCAGTCTTTACCGTTCAGCAGGAAGAT
GACCCGGAAACGGGCATCAACAAGGGCCACCTGGTATTGACCAAGGAGCTCGTCAAT
AGATTGGCCAAGGAGCAGGCTGAACCTCCGGAAGACCCCAGCATGAAGATTGGATGG
GAGGGATTGATTAGGGCTGGTGCGGTTGAATATCTCGACGCCGAGGAAGAGGAGACGT
CCATGATCTGCATGACGCCAGAGGATCTCGAGCTGTATCGTCTTCAGAAGGCCGGTATT
AACACTGAGGAAGACATGGGAGATGATCCGAACAAGCGACTGAAGACCAAGACGAA
CCCGACAACTCACATGTACACCCATTGCGAGATTCACCCAAGTATGATCTTAGGTATCT
GTGCTAGTATCATTCCTTTCCCCGATCACAATCAGGTATGTTGTCCACCCTTCCAGTCTT
AT

>KX632571.1 *Trichoderma atrobrunneum* T39
CCTGCTGGCCAAGCTGTTCCGTGGTATCATGCGAAGGATGAACACTGAATTGGCCAAC
TATCTGAGACGATGCGTTGAGGGTAACCGACACTTCAACCTTGCTGTTGGTATCAAGCC
CGGCACGCTCTCCAACGGATTGAAGTATTCGCTTGCCACAGGAAACTGGGGTGATCAG
AAGAAGGCCATGAGCTCAACTGCAGGTGTGTCCCAGGTGCTTAACCGTTACACGTTTG
CTTCGACCCTATCACATTTGCGTCGTACCAATACTCCTATCGGAAGAGATGGTAAGCTG
GCAAAGCCTCGTCAGCTTCACAACACGCATTGGGGTTTGGTCTGCCCAGCCGAGACAC
CCGAAGGACAGGCCTGTGGTCTGGTCAAGAACTTGTCTTTGATGTGTTACGTCAGTGT
CGGTTCTCCCTCCGAACCTCTGATTGAGTTCATGATCAACAGAGGTATGGAAGTTGTCG
AAGAATACGAGCCGCTGCGCTATCCTCATGCTACAAAGATTTTTGTGAACGGTGTCTGG
GTTGGAGTTCATCAAGACCCTAAGCACTTGGTGAACCAGGTTCTAGATACTCGTCGCA
AGTCCTATCTGCAATACGAAGTCTCTCTCGTGAGAGAAATTCGAGACCAGGAATTCAA
AATCTTTTCCGACGCAGGCCGTGTCATGAGACCAGTCTTTACCGTTCAGCAGGAAGAT
GACCCGGAAACGGGCATCAACAAGGGCCACCTGGTATTGACCAAGGAGCTCGTCAAT
AGATTGGCCAAGGAGCAGGCTGAACCTCCGGAAGACCCCAGCATGAAGATTGGATGG

GAGGGATTGATTAGGGCTGGTGCGGTTGAATATCTCGACGCCGAGGAAGAGGAGACGT
CCATGATCTGCATGACGCCAGAGGATCTCGAGCTGTATCGTCTTCAGAAGGCCGGTATT
AACACTGAGGAAGACATGGGAGATGATCCGAACAAGCGACTGAAGACCAAGACGAA
CCCGACAACTCACATGTACACCCATTGCGAGATTCACCCAAGTATGATCTTAGGTATCT
GTGCTAGTATCATTCCTTTCCCCGATCACAATCAGGTATGTTGTCCACCCTTCCAGTCTT

>KJ665241.1 *Trichoderma atrobrunneum* S3
TGCTGGCCAAGCTGTTCCGTGGTATCATGCGAAGGATGAACACTGAGTTGGCCAACTA
CCTGAGACGATGCGTTGAGGGCAACCGACACTTCAACCTGGCTGTGGGTATCAAGCCC
GGCACGCTTTCAAACGGATTGAAGTATTCGCTTGCCACAGGAAACTGGGGTGATCAGA
AGAAGGCCATGAGCTCAACTGCAGGCGTGTCCCAGGTGCTTAACCGATACACGTTTGC
TTCGACCCTGTCACATTTGCGTCGTACCAACACCCCTATCGGAAGAGATGGTAAGCTGG
CAAAGCCTCGACAGCTTCACAACACGCATTGGGGTTTGGTCTGCCCAGCCGAGACAC
CCGAAGGACAGGCCTGTGGTCTGGTCAAGAACTTGTCTTTGATGTGTTACGTCAGTGT
CGGTTCTCCCTCTGAGCCTCTGATTGAGTTCATGATCAACAGAGGTATGGAAGTTGTCG
AAGAGTACGAGCCGCTGAGGTATCCTCATGCTACAAAGATTTTTGTGAACGGTGTCTG
GGTTGGAGTTCACCAAGACCCTAAGCACTTGGTGAACCAGGTTCTGGATACTCGTCGC
AAGTCCTATCTGCAATACGAAGTCTCTCTCGTGAGAGAAATTCGAGACCAGGAATTTA
AAATCTTTTCCGACGCAGGTCGTGTCATGCGACCAGTCTTTACCGTTCAACAGGAAGA
TGACCCGGAAACGGGCATCAACAAGGGCCACCTGGTATTGACCAAGGAGCTCGTCAA
TAGATTGGCCAAGGAGCAGGCTGAGCCTCCGGAAGACCCCAGCATGAAGATTGGATG
GGAGGGATTGATCAGGGCTGGTGCGGTTGAATATCTCGACGCCGAGGAAGAGGAGAC
GTCCATGATCTGCATGACGCCAGAGGATCTCGAGCTGTATCGTCTTCAGAAGGCCGGTA
TTAACACTGAGGAAGACATGGGAGATGACCCGAACAAGCGACTAAAGACCAAGACGA
ACCCGACAACTCACATGTACACCCATTGCGAGATTCACCCAAGTATGATCTTAGGTATC
TGTGCTAGTATCATTCCTTTCCCCGATCACAACCAGGTATG

>FJ442777.1 *Trichoderma atrobrunneum* GJS 98-183
CCTGCTGGCCAAGCTGTTCCGTGGTATCATGCGAAGGATGAACACTGAGTTGGCCAAC
TACCTGAGACGATGCGTTGAGGGCAACCGACATTTCAACCTTGCTGTTGGTATCAAGC
CCGGCACGCTTTCAAACGGATTGAAGTATTCGCTTGCCACAGGCAACTGGGGTGATCA
GAAGAAGGCCATGAGCTCAACTGCAGGTGTGTCCCAGGTGCTTAACCGTTACACGTTT
GCTTCGACCTTGTCGCATTTGCGTCGTACCAATACTCCTATCGGAAGAGATGGTAAGCT
GGCAAAGCCTCGACAGCTTCACAACACGCATTGGGGTTTGGTCTGCCCAGCCGAGAC
ACCCGAAGGACAGGCTTGTGGTCTGGTCAAGAACTTGTCTTTGATGTGTTACGTCAGT
GTCGGTTCTCCCTCTGAACCTCTCATTGAGTTCATGATCAACAGAGGTATGGAAGTCGT
CGAAGAGTACGAGCCTCTGCGGTATCCTCATGCTACAAAGATTTTTGTGAACGGTGTCT
GGGTTGGAGTCCACCAAGACCCTAAGCACTTGGTGAACCAGGTTCTGGACACTCGTC
GCAAGTCCTATCTGCAATACGAAGTCTCTCTCGTGAGAGAAATTCGAGACCAGGAATT
CAAAATCTTTTCCGACGCTGGCCGTGTCATGCGACCAGTCTTTACCGTTCAACAGGAA
GATGACCCGGAAACGGGCATCAACAAGGGCCACCTGGTATTGACCAAGGAGCTCGTC
AATAGATTGGCCAAGGAGCAGGCTGAGCCTCCGGAAGACCCCAGCATGAAGATTGGAT
GGGAGGGATTGATCAGGGCTGGTGCGGTTGAATATCTCGACGCCGAGGAAGAGGAGA
CGTCCATGATCTGCATGACGCCAGAGGATCTCGAGCTGTATCGTCTTCAGAAGGCCGGT

ATTAACACTGAGGAAGACATGGGAGATGACCCGAACAAGCGACTAAAGACCAAGACC
AACCCGACAACTCACATGTACACCCATTGCGAGATTCACCCAAGTATGATCTTAGGCAT
CTGTGCTAGTA

>FJ442724.1 *Trichoderma atrobrunneum* GJS 04-67
CCTGCTGGCCAAGCTGTTCCGTGGTATCATGCGAAGGATGAACACTGAGTTGGCCAAC
TACCTGAGACGATGCGTTGAGGGCAACCGACACTTCAACCTGGCTGTGGGTATCAAGC
CCGGCACGCTTTCAAACGGATTGAAGTATTCGCTTGCCACAGGAAACTGGGGTGATCA
GAAGAAGGCCATGAGCTCAACTGCAGGCGTGTCCCAGGTGCTTAACCGATACACGTTT
GCTTCGACCCTGTCACATTTGCGTCGTACCAACACCCCTATCGGAAGAGATGGTAAGCT
GGCAAAGCCTCGACAGCTTCACAACACGCATTGGGGTTTGGTCTGCCCAGCCGAGAC
ACCCGAAGGACAGGCCTGTGGTCTGGTCAAGAACTTGTCTTTGATGTGTTACGTCAGT
GTCGGTTCTCCCTCTGAGCCTCTGATTGAGTTCATGATCAACAGAGGTATGGAAGTTGT
CGAAGAGTACGAGCCGCTGAGGTATCCTCATGCTACAAAGATTTTTGTGAACGGTGTC
TGGGTTGGAGTTCACCAAGACCCTAAGCACTTGGTGAACCAGGTTCTGGATACTCGTC
GCAAGTCCTATCTGCAATACGAAGTCTCTCTCGTGAGAGAAATTCGAGACCAGGAATT
TAAAATCTTTTCCGACGCAGGTCGTGTCATGCGACCAGTCTTTACCGTTCAACAGGAA
GATGACCCGGAAACGGGCATCAACAAGGGCCACCTGGTATTGACCAAGGAGCTCGTC
AATAGATTGGCCAAGGAGCAGGCTGAGCCTCCGGAAGACCCCAGCATGAAGATTGGAT
GGGAGGGATTGATCAGGGCTGGTGCGGTTGAATATCTCGACGCCGAGGAAGAGGAGA
CGTCCATGATCTGCATGACGCCAGAGGATCTCGAGCTGTATCGTCTTCAGAAGGCCGGT
ATTAACACTGAGGAAGACATGGGAGATGACCCGAACAAGCGACTAAAGACCAAGACG
AACCCGACAACTCACATGTACACCCATTGCGAGATTCACCCAAGTATGATCTTAGGTAT
CTGTGCTAGTA

>MZ675866.1 *Trichoderma camerunense* Vimi-17.0025
cdsTGTTCCGTGGTATCATGCGAAGGATGAACACTGAATTGGCCAACTACCTGAGACGG
TGTGTTGAGGGTAACCGACACTTCAACCTTGCTGTTGGTATCAAGCCCGGCACGCTCT
CAAACGGATTGAAGTATTCGCTTGCCACAGGAAACTGGGGTGATCAGAAGAAGGCCAT
GAGCTCGACTGCAGGTGTGTCACAGGTGCTCAACCGTTACACGTTTGCTTCGACCTTG
TCACATTTGCGTCGTACCAATACTCCTATCGGAAGAGATGGTAAGCTGGCAAAGCCTCG
ACAGCTTCACAACACGCATTGGGGTTTGGTCTGTCCTGCCGAGACACCCGAAGGACA
GGCCTGTGGTCTGGTCAAGAACTTGTCTTTGATGTGTTACGTCAGTGTCGGTTCTCCCT
CCGAGCCTCTGATTGAATTCATGATCAACAGAGGTATGGAGGTCGTCGAAGAGTATGA
GCCGCTGCGGTATCCTCATGCTACAAAGATTTTTGTGAACGGTGTCTGGGTTGGAGTTC
ACCAAGACCCTAAGCACTTGGTGAACCAGGTTCTGGACACTCGTCGCAAGTCCTATCT
GCAGTACGAAGTCTCTCTCGTGAGAGAAATTCGAGACCAGGAATTCAAAATCTTTTCC
GACGCAGGCCGTGTCATGCGACCAGTCTTTACCGTTCAGCAGGAAGATGACCCGGAA
ACGGGCATCAACAAGGGTCACCTGGTATTGACCAAGGAGCTCGTCAATAGATTGGCCA
AGGAGCAGGCTGAGCCTCCGGAAGATCCCAGCATGAAGATCGGATGGGAGGGATTAAT
CAGGGCTGGTGCGGTTGAATATCTCGACGCCGAGGAAGAGGAGACGTCCATGATCTGC
ATGACGCCAGAGGATCTCGAGCTGTATCGTCTTCAGAAGGCCGGTATTAACACCGAGG
AAGACATGGGAGATGACCCGAACAAGCGATTAAAGACCAAGACAAACCCGACAACTC
ACATGTACACCCATTGCGAGATTCACCCAAGTATGATCTTAGGCATCTGTGCTAGTATCA

TTCCTTTCCCCGA

>MZ675855.1 *Trichoderma camerunense* Vimi-17.0043
CTGTTCCGTGGTATCATGCGAAGGATGAACACTGAATTGGCCAACTACCTGAGACGGT
GTGTTGAGGGTAACCGACACTTCAACCTTGCTGTTGGTATCAAGCCCGGCACGCTCTC
AAACGGATTGAAGTATTCGCTTGCCACAGGAAACTGGGGTGATCAGAAGAAGGCCAT
GAGCTCGACTGCAGGTGTGTCACAGGTGCTCAACCGTTACACGTTTGCTTCGACCTTG
TCACATTTGCGTCGTACCAATACTCCTATCGGAAGAGATGGTAAGCTGGCAAAGCCTCG
ACAGCTTCACAACACGCATTGGGGTTTGGTCTGTCCTGCCGAGACACCCGAAGGACA
GGCCTGTGGTCTGGTCAAGAACTTGTCTTTGATGTGTTACGTCAGTGTCGGTTCTCCCT
CCGAGCCTCTGATTGAATTCATGATCAACAGAGGTATGGAGGTCGTCGAAGAGTATGA
GCCGCTGCGGTATCCTCATGCTACAAAGATTTTTGTGAACGGTGTCTGGGTTGGAGTTC
ACCAAGACCCTAAGCACTTGGTGAACCAGGTTCTGGACACTCGTCGCAAGTCCTATCT
GCAGTACGAAGTCTCTCTCGTGAGAGAAATTCGAGACCAGGAATTCAAAATCTTTTCC
GACGCAGGCCGTGTCATGCGACCAGTCTTTACCGTTCAGCAGGAAGATGACCCGGAA
ACGGGCATCAACAAGGGTCACCTGGTATTGACCAAGGAGCTCGTCAATAGATTGGCCA
AGGAGCAGGCTGAGCCTCCGGAAGATCCCAGCATGAAGATCGGATGGGAGGGATTAAT
CAGGGCTGGTGCGGTTGAATATCTCGACGCCGAGGAAGAGGAGACGTCCATGATCTGC
ATGACGCCAGAGGATCTCGAGCTGTATCGTCTTCAGAAGGCCGGTATTAACACCGAGG
AAGACATGGGAGATGACCCGAACAAGCGATTAAAGACCAAGACAAACCCGACAACTC
ACATGTACACCCATTGCGAGATTCACCCAAGTATGATCTTAGGCATCTGTGCTAGTATCA
TTCCTTTCCCCG

>MZ675854.1 *Trichoderma camerunense* Vimi-17.0045
CGTGGTATCATGCGAAGGATGAACACTGAATTGGCCAACTACCTGAGACGGTGTGTTG
AGGGTAACCGACACTTCAACCTTGCTGTTGGTATCAAGCCCGGCACGCTCTCAAACGG
ATTGAAGTATTCGCTTGCCACAGGAAACTGGGGTGATCAGAAGAAGGCCATGAGCTCG
ACTGCAGGTGTGTCACAGGTGCTCAACCGTTACACGTTTGCTTCGACCTTGTCACATTT
GCGTCGTACCAATACTCCTATCGGAAGAGATGGTAAGCTGGCAAAGCCTCGACAGCTT
CACAACACGCATTGGGGTTTGGTCTGTCCTGCCGAGACACCCGAAGGACAGGCCTGT
GGTCTGGTCAAGAACTTGTCTTTGATGTGTTACGTCAGTGTCGGTTCTCCCTCCGAGCC
TCTGATTGAATTCATGATCAACAGAGGTATGGAGGTCGTCGAAGAGTATGAGCCGCTG
CGGTATCCTCATGCTACAAAGATTTTTGTGAACGGTGTCTGGGTTGGAGTTCACCAAGA
CCCTAAGCACTTGGTGAACCAGGTTCTGGACACTCGTCGCAAGTCCTATCTGCAGTAC
GAAGTCTCTCTCGTGAGAGAAATTCGAGACCAGGAATTCAAAATCTTTTCCGACGCAG
GCCGTGTCATGCGACCAGTCTTTACCGTTCAGCAGGAAGATGACCCGGAAACGGGCAT
CAACAAGGGTCACCTGGTATTGACCAAGGAGCTCGTCAATAGATTGGCCAAGGAGCA
GGCTGAGCCTCCGGAAGATCCCAGCATGAAGATCGGATGGGAGGGATTAATCAGGGCT
GGTGCGGTTGAATATCTCGACGCCGAGGAAGAGGAGACGTCCATGATCTGCATGACGC
CAGAGGATCTCGAGCTGTATCGTCTTCAGAAGGCCGGTATTAACACCGAGGAAGACAT
GGGAGATGACCCGAACAAGCGATTAAAGACCAAGACAAACCCGACAACTCACATGTA
CACCCATTGCGAGATTCACCCAAGTATGATCTTAGGCATCTGTGCTAGTATCATTCCTTT
CCCCGATC

>MZ675862.1 *Trichoderma camerunense* Vimi-17.0034
CGAAGGATGAACACTGAATTGGCCAACTACCTGAGACGGTGTGTTGAGGGTAACCGA
CACTTCAACCTTGCTGTTGGTATCAAGCCCGGCACGCTCTCAAACGGATTGAAGTATTC
GCTTGCCACAGGAAACTGGGGTGATCAGAAGAAGGCCATGAGCTCGACTGCAGGTGT
GTCACAGGTGCTCAACCGTTACACGTTTGCTTCGACCTTGTCACATTTGCGTCGTACCA
ATACTCCTATCGGAAGAGATGGTAAGCTGGCAAAGCCTCGACAGCTTCACAACACGCA
TTGGGGTTTGGTCTGTCCTGCCGAGACACCCGAAGGACAGGCCTGTGGTCTGGTCAA
GAACTTGTCTTTGATGTGTTACGTCAGTGTCGGTTCTCCCTCCGAGCCTCTGATTGAAT
TCATGATCAACAGAGGTATGGAGGTCGTCGAAGAGTATGAGCCGCTGCGGTATCCTCAT
GCTACAAAGATTTTTGTGAACGGTGTCTGGGTTGGAGTTCACCAAGACCCTAAGCACT
TGGTGAACCAGGTTCTGGACACTCGTCGCAAGTCCTATCTGCAGTACGAAGTCTCTCT
CGTGAGAGAAATTCGAGACCAGGAATTCAAAATCTTTTCCGACGCAGGCCGTGTCATG
CGACCAGTCTTTACCGTTCAGCAGGAAGATGACCCGGAAACGGGCATCAACAAGGGT
CACCTGGTATTGACCAAGGAGCTCGTCAATAGATTGGCCAAGGAGCAGGCTGAGCCTC
CGGAAGATCCCAGCATGAAGATCGGATGGGAGGGATTAATCAGGGCTGGTGCGGTTGA
ATATCTCGACGCCGAGGAAGAGGAGACGTCCATGATCTGCATGACGCCAGAGGATCTC
GAGCTGTATCGTCTTCAGAAGGCCGGTATTAACACCGAGGAAGACATGGGAGATGACC
CGAACAAGCGATTAAAGACCAAGACAAACCCGACAACTCACATGTACACCCATTGCG
AGATTCACCCAAGTATGATCTTAGGCATCTGTGCTAGTATCATTCCTTTCCCCGATCACA
ACCAG

>OR548108.1 *Trichoderma endophyticum* 99
GAATTGGCCAACTACCTGAGACGGTGTGTTGAGGGTAACCGACACTTCAACCTTGCTG
TTGGTATCAAGCCCGGCACGCTCTCAAACGGATTGAAGTATTCGCTTGCCACAGGAAA
CTGGGGTGATCAGAAGAAGGCCATGAGCTCGACTGCAGGTGTGTCACAGGTGCTTAA
CCGTTACACGTTTGCTTCGACCTTGTCACATTTGCGTCGTACCAATACTCCTATCGGAA
GAGATGGTAAGCTGGCAAAGCCTCGACAGCTTCACAACACGCATTGGGGTTTGGTCTG
TCCTGCCGAGACACCCGAAGGACAGGCCTGTGGTCTGGTCAAGAACTTGTCTTTGATG
TGTTACGTCAGTGTCGGTTCTCCCTCCGAGCCTCTGATTGAATTCATGATCAACAGAGG
TATGGAGGTCGTCGAAGAGTATGAGCCGCTGCGGTATCCTCATGCTACAAAGATTTTTG
TGAACGGTGTCTGGGTTGGAGTTCACCAAGACCCTAAGCACTTGGTGAACCAGGTTCT
GGATACTCGTCGCAAGTCCTATCTGCAATACGAAGTCTCTCTCGTGAGAGAAATTCGAG
ACCAGGAATTCAAAATCTTTTCCGACGCAGGCCGTGTCATGCGACCAGTCTTTACCGTT
CAGCAGGAAGATGACCCGGAAACGGGCATCAACAAGGGCCACCTGGTTTTGACCAAG
GAGCTCGTCAATAGATTGGCCAAGGAGCAAGCTGAACCTCCGGAAGACCCCAGCATG
AAGATCGGATGGGAGGGACTGATTAGGGCTGGTGCGGTTGAATATCTCGACGCCGAGG
AAGAGGAGACGTCCATGATCTGCATGACGCCTGAGGATCTCGAGCTGTATCGCCTTCA
GAAGGCCGGTATTAACACTGAGGAAGACATGGGAGATGATCCGAACAAGCGACTAAA
GACGAAGACGAACCCGACAACTCATATGTACACCCACTGCGAGATTCACCCAAGTATG
ATCTTAGGTATCTGTGCTAGTATCATTCCTTTCCCCGATCACAACCAGGTAT

>PP500719.1 *Trichoderma rifaii* 77JCR
TGCTGGCCAAGCTGTTCCGTGGTATCATGCGAAGGATGAACACTGAATTGGCCAACTAT
CTGAGACGATGCGTTGAGGGTAACCGACACTTCAACCTTGCTGTCGGTATCAAGCCCG

GCACGCTCTCAAACGGATTGAAGTATTCGCTTGCCACAGGAAACTGGGGTGATCAGAA
GAAGGCCATGAGCTCAACTGCAGGTGTGTCCCAGGTGCTTAACCGTTACACATTTGCT
TCGACGCTGTCACATTTGCGTCGTACCAACACTCCTATCGGAAGAGATGGTAAGCTGG
CAAAGCCTCGACAGCTTCACAACACGCATTGGGGTTTGGTCTGCCCAGCCGAGACAC
CCGAAGGACAGGCCTGTGGTCTGGTCAAGAACTTGTCTTTGATGTGTTACGTCAGTGT
CGGTTCTCCCTCTGAACCTCTGATTGAGTTCATGATCAACAGAGGTATGGAAGTCGTCG
AAGAGTACGAGCCGCTGCGGTATCCTCATGCTACAAAGATTTTTGTGAACGGTGTCTG
GGTTGGAGTTCACCAAGACCCTAAGCACTTGGTGAACCAGGTTCTGGACACTCGTCGC
AAGTCCTATCTGCAGTACGAAGTCTCTCTCGTGAGAGAAATTCGAGACCAGGAATTCA
AAATCTTTTCCGACGCAGGCCGTGTCATGCGACCAGTCTTTACCGTTCAGCAGGAAGA
TGACCCGGAAACGGGCATCAACAAGGGCCACCTGGTATTGACCAAGGAGCTCGTCAA
TAGATTGGCCAAGGAGCAAGCTGAACCTCCGGAAGACCCCAGCATGAAGATCGGATG
GGAGGGACTGATTAGGGCTGGTGCGGTTGAATATCTCGACGCCGAGGAAGAGGAGAC
GTCCATGATCTGCATGACGCCTGAGGATCTCGAGCTGTATCGCCTTCAGAAGGCCGGTA
TTAACACTGAGGAAGACATGGGAGATGATCCGAACAAGCGACTAAAGACGAAGACGA
ACCCGACAACTCATATGTACACCCACTGCGAGATTCACCCAAGTATGATCTTAGGGATC
TGTGCTAGTATCATTCCTTTCCCCGATC

>PP500718.1 *Trichoderma rifaii* 73JES
TGCTGGCCAAGCTGTTCCGTGGTATCATGCGAAGGATGAACACTGAATTGGCCAACTAT
CTGAGACGATGCGTTGAGGGTAACCGACACTTCAACCTTGCTGTCGGTATCAAGCCCG
GCACGCTCTCAAACGGATTGAAGTATTCGCTTGCCACAGGAAACTGGGGTGATCAGAA
GAAGGCCATGAGCTCAACTGCAGGTGTGTCCCAGGTGCTTAACCGTTACACATTTGCT
TCGACGCTGTCACATTTGCGTCGTACCAACACTCCTATCGGAAGAGATGGTAAGCTGG
CAAAGCCTCGACAGCTTCACAACACGCATTGGGGTTTGGTCTGCCCAGCCGAGACAC
CCGAAGGACAGGCCTGTGGTCTGGTCAAGAACTTGTCTTTGATGTGTTACGTCAGTGT
CGGTTCTCCCTCTGAACCTCTGATTGAGTTCATGATCAACAGAGGTATGGAAGTCGTCG
AAGAGTACGAGCCGCTGCGGTATCCTCATGCTACAAAGATTTTTGTGAACGGTGTCTG
GGTTGGAGTTCACCAAGACCCTAAGCACTTGGTGAACCAGGTTCTGGACACTCGTCGC
AAGTCCTATCTGCAGTACGAAGTCTCTCTCGTGAGAGAAATTCGAGACCAGGAATTCA
AAATCTTTTCCGACGCAGGCCGTGTCATGCGACCAGTCTTTACCGTTCAGCAGGAAGA
TGACCCGGAAACGGGCATCAACAAGGGCCACCTGGTATTGACCAAGGAGCTCGTCAA
TAGATTGGCCAAGGAGCAAGCTGAACCTCCGGAAGACCCCAGCATGAAGATCGGATG
GGAGGGACTGATTAGGGCTGGTGCGGTTGAATATCTCGACGCCGAGGAAGAGGAGAC
GTCCATGATCTGCATGACGCCTGAGGATCTCGAGCTGTATCGCCTTCAGAAGGCCGGTA
TTAACACTGAGGAAGACATGGGAGATGATCCGAACAAGCGACTAAAGACGAAGACGA
ACCCGACAACTCATATGTACACCCACTGCGAGATTCACCCAAGTATGATCTTAGGGATC
TGTGCTAGTATCATTCCTTTCCCCGATC

>FJ442720.1 *Trichoderma rifaii* DIS 337F
CCTGCTGGCCAAGCTGTTCCGTGGTATCATGCGAAGGATGAACACTGAATTGGCCAAC
TACCTGAGACGGTGTGTTGAGGGTAACCGACACTTCAACCTTGCTGTTGGTATCAAGC
CCGGCACGCTCTCAAACGGATTGAAGTATTCGCTTGCCACAGGAAACTGGGGTGATCA
GAAGAAGGCCATGAGCTCAACTGCAGGCGTGTCCCAGGTGCTTAACCGTTACACGTTT

GCTTCGACCCTATCACATTTGCGTCGTACCAACACTCCTATCGGAAGAGATGGTAAGCT
GGCAAAGCCTCGACAGCTTCACAACACGCATTGGGGTTTGGTCTGCCCAGCCGAGAC
ACCCGAAGGACAGGCCTGTGGTCTGGTCAAAAACCTGTCTTTGATGTGTTACGTCAGT
GTCGGTTCTCCCTCCGAACCTCTGATTGAGTTCATGATCAACAGAGGTATGGAAGTCGT
TGAAGAGTACGAGCCGCTGCGGTATCCTCATGCTACAAAGATTTTTGTGAACGGTGTCT
GGGTTGGAGTTCACCAAGACCCTAAGCACTTGGTGAACCAGGTCCTGGACACTCGTC
GCAAGTCCTATCTGCAATACGAAGTCTCTCTCGTGAGAGAAATTCGAGACCAGGAATT
CAAAATCTTTTCCGACGCAGGCCGTGTCATGCGACCAGTCTTTACCGTTCAGCAGGAA
GATGACCCGGAAACGGGCATCAACAAGGGCCACCTGGTTTTGACCAAGGAGCTCGTC
AATAGATTGGCCAAGGAGCAAGCTGAACCTCCGGAAGACCCCAGCATGAAGATCGGA
TGGGAGGGACTGATTAGGGCTGGTGCGGTTGAATATCTCGACGCCGAGGAAGAGGAG
ACGTCCATGATCTGCATGACGCCTGAGGATCTCGAGCTGTATCGCCTTCAGAAGGCCG
GTATTAACACTGAGGAAGACATGGGGGATGATCCGAACAAGCGACTAAAGACGAAGA
CGAACCCGACAACTCATATGTACACCCACTGCGAGATTCACCCAAGTATGATCTTAGGT
ATCTGTGCTAGTA

>KJ665337.1 *Trichoderma simmonsii* S7
TGCTGGCTAAGCTGTTCCGTGGTATCATGCGAAGGATGAACACTGAATTGGCCAACTAT
CTGAGACGGTGCGTTGAGGGCAACCGACACTTCAACCTGGCTGTGGGTATCAAGCCC
GGCACACTTTCAAACGGATTGAAGTATTCGCTTGCCACAGGAAACTGGGGTGATCAGA
AGAAGGCCATGAGCTCAACTGCTGGTGTGTCTCAGGTGCTTAACCGTTACACATTTGC
TTCGACCTTGTCACATTTGCGTCGTACCAACACCCCTATCGGAAGAGATGGTAAGCTGG
CAAAGCCTCGACAGCTTCACAACACGCATTGGGGTTTGGTCTGCCCAGCCGAGACAC
CCGAAGGACAGGCTTGTGGTCTGGTCAAGAACTTGTCTTTGATGTGTTACGTCAGTGT
CGGTTCTCCCTCTGAACCTCTGATTGAGTTCATGATCAACAGAGGTATGGAAGTCGTCG
AAGAATACGAGCCTCTGCGATATCCTCATGCTACAAAGATTTTTGTGAACGGTGTCTGG
GTTGGAGTTCACCAAGACCCTAAGCACTTGGTGAACCAGGTTCTGGATACTCGTCGCA
AGTCCTATCTGCAATACGAAGTCTCTCTCGTGAGAGAAATTCGAGACCAGGAATTCAA
AATCTTTTCCGACGCAGGCCGTGTCATGCGACCTGTCTTTACCGTTCAGCAGGAAGATG
ACCCCGAAACGGGCATCAACAAGGGCCACCTTGTATTGACCAAGGAACTCGTCAATAG
ATTGGCCAAGGAGCAGGCTGAGCCTCCGGAAGACCCCAGCATGAAGATTGGATGGGA
GGGATTGATCAGGGCTGGTGCGGTTGAATATCTCGACGCCGAGGAAGAGGAGACGTCC
ATGATCTGCATGACGCCAGAGGATCTCGAGCTGTATCGTCTTCAGAAGGCCGGTATTAA
CACCGAGGAAGACATGGGAGATGATCCGAACAAGCGATTGAAGACCAAGACGAACCC
GACAACCCATATGTACACCCACTGCGAGATTCACCCAAGTATGATCTTAGGTATCTGTG
CTAGTATCATTCCTTTCCCCGATCACAACCAGGTATG

>FJ442710.1 *Trichoderma simmonsii* GJS 92-100
GGTCCCTTGCTGGCTAAGCTGTTCCGTGGTATCATGCGAAGGATGAACACTGAATTGGC
CAACTATCTGAGACGGTGCGTTGAGGGCAACCGACACTTCAACCTGGCTGTGGGTATC
AAGCCCGGCACACTTTCAAACGGATTGAAGTATTCGCTTGCCACAGGAAACTGGGGTG
ATCAGAAGAAGGCCATGAGCTCAACTGCTGGTGTGTCTCAGGTGCTTAACCGTTACAC
ATTTGCTTCGACCTTGTCACATTTGCGTCGTACCAACACCCCTATCGGAAGAGATGGTA
AGCTGGCAAAGCCTCGACAGCTTCACAACACGCATTGGGGTTTGGTCTGCCCAGCCG

AGACACCCGAAGGACAGGCTTGTGGTCTGGTCAAGAACTTGTCTTTGATGTGTTACGT
CAGTGTCGGTTCTCCCTCTGAACCTCTGATTGAGTTCATGATCAACAGAGGTATGGAAG
TCGTCGAAGAATACGAGCCTCTGCGATATCCTCATGCTACAAAGATTTTTGTGAACGGT
GTCTGGGTTGGAGTTCACCAAGACCCTAAGCACTTGGTGAACCAGGTTCTGGATACTC
GTCGCAAGTCCTATCTGCAATACGAAGTCTCTCTCGTGAGAGAAATTCGAGACCAGGA
ATTCAAAATCTTTTCCGACGCAGGCCGTGTCATGCGACCTGTCTTTACCGTTCAGCAGG
AAGATGACCCCGAAACGGGCATCAACAAGGGCCACCTTGTATTGACCAAGGAACTCG
TCAATAGATTGGCCAAGGAGCAGGCTGAGCCTCCGGAAGACCCCAGCATGAAGATTG
GATGGGAGGGATTGATCAGGGCTGGTGCGGTTGAATATCTCGACGCCGAGGAAGAGG
AGACGTCCATGATCTGCATGACGCCAGAGGATCTCGAGCTGTATCGTCTTCAGAAGGC
CGGTATTAACACCGAGGAAGACATGGGAGATGATCCGAACAAGCGACTAAAGACCAA
GACGAACCCGACAACTCATATGTACACCCACTGCGAGATTCACCCAAGTATGATCTTAG
GTATCTGTGCTAGTA

>FJ442757.1 *Trichoderma simmonsii* GJS 91-138
GGTCCCTTGCTGGCTAAGCTGTTCCGTGGTATCATGCGAAGGATGAACACTGAATTGGC
CAACTATCTGAGACGGTGCGTTGAGGGCAACCGACACTTCAACCTGGCTGTGGGTATC
AAGCCCGGCACACTTTCAAACGGATTGAAGTATTCGCTTGCCACAGGAAACTGGGGTG
ATCAGAAGAAGGCCATGAGCTCAACTGCTGGTGTGTCTCAGGTGCTTAACCGTTACAC
ATTTGCTTCGACCTTGTCACATTTGCGTCGTACCAACACCCCTATCGGAAGAGATGGTA
AGCTGGCAAAGCCTCGACAGCTTCACAACACGCATTGGGGTTTGGTCTGCCCAGCCG
AGACACCCGAAGGACAGGCTTGTGGTCTGGTCAAGAACTTGTCTTTGATGTGTTACGT
CAGTGTCGGTTCTCCCTCTGAACCTCTGATTGAGTTCATGATCAACAGAGGTATGGAAG
TCGTCGAAGAATACGAGCCTCTGCGATATCCTCATGCTACAAAGATTTTTGTGAACGGT
GTCTGGGTTGGAGTTCACCAAGACCCTAAGCACTTGGTGAACCAGGTTCTGGATACTC
GTCGCAAGTCCTATCTGCAATACGAAGTCTCTCTCGTGAGAGAAATTCGAGACCAGGA
ATTCAAAATCTTTTCCGACGCAGGCCGTGTCATGCGACCTGTCTTTACCGTTCAGCAGG
AAGATGACCCCGAAACGGGCATCAACAAGGGCCACCTTGTATTGACCAAGGAACTCG
TCAATAGATTGGCCAAGGAGCAGGCTGAGCCTCCGGAAGACCCCAGCATGAAGATTG
GATGGGAGGGATTGATCAGGGCTGGTGCGGTTGAATATCTCGACGCCGAGGAAGAGG
AGACGTCCATGATCTGCATGACGCCAGAGGATCTCGAGCTGTATCGTCTTCAGAAGGC
CGGTATTAACACCGAGGAAGACATGGGAGATGATCCGAACAAGCGATTGAAGACCAA
GACGAACCCGACAACCCATATGTACACCCACTGCGAGATTCACCCAAGTATGATCTTAG
GTATCTGTGCTAGTA

>MT587315.1 *Trichoderma lixii* TLiC8
GGCCAAGCTATTCCGTGGTATCATGCGAAGGATGAACACTGAATTGGCCAACTATCTGA
GACGGTGCGTTGAGGGCAACCGACACTTCAACCTGGCCGTGGGTATCAAGCCAGGCA
CGCTTTCAAACGGATTGAAGTATTCGCTTGCTACAGGAAACTGGGGTGATCAGAAGAA
GGCCATGAGCTCAACTGCAGGTGTGTCTCAGGTGCTTAATCGTTACACGTTTGCCTCAA
CCTTGTCGCATTTGCGTCGTACCAACACTCCCATCGGAAGAGATGGTAAGCTGGCAAA
GCCTCGACAGCTTCACAACACGCATTGGGGTTTGGTCTGCCCAGCCGAGACGCCCGA
AGGACAGGCTTGTGGTCTGGTCAAGAACTTGTCTTTGATGTGTTACGTCAGTGTCGGT
TCTCCTTCCGAACCTCTGATTGAGTTCATGATCAACAGAGGTATGGAAGTCGTCGAAG

AGTATGAGCCGCTGCGGTATCCTCATGCTACAAAGATTTTTGTGAACGGTGTCTGGGTT
GGAGTTCACCAAGACCCTAAGCACTTGGTGAACCAGGTTCTGGACACTCGTCGCAAG
TCCTATCTGCAGTACGAAGTCTCTCTCGTGAGAGAAATTCGAGACCAGGAATTCAAAA
TCTTTTCCGACGCAGGTCGTGTCATGCGACCAGTCTTTACCGTTCAGCAGGAAGATGA
CCCGGAAACGGGCATCAACAAGGGCCACCTGGTATTGACCAAGGAGCTCGTCAATAG
ATTGGCTAAGGAGCAGGCTGAGCCTCCGGAAGACCCCAGCATGAAGATTGGATGGGA
GGGGTTGATTAGGGCTGGTGCAGTTGAATATCTCGACGCTGAGGAAGAGGAGACGTCC
ATGATCTGCATGACGCCAGAGGATCTCGAGCTGTATCGTCTTCAGAAGGCCGGTATTAA
CACTGAGGAAGACATGGGAGATGATCCGAACAAGCGACTGAAGACCAAGACGAACCC
GACAACTCACATGTACACCCATTGCGAGATTCACCCAAGTATGATCTTAGGTATCTGTG
CTAGCATCATTCCTTTCCCCGATCACAACCAGGTATGTTGTCCACCTCCCAGTCTTATCA
ACGAGGAAAATACTAACGGTGTGTATA

>FJ442771.1 *Trichoderma lixii* GJS 97-106
CCTGCTGGCCAAGCTGTTCCGTGGCATCATGCGAAGGATGAACACTGAATTGGCCAAC
TACCTGAGACGGTGTGTTGAGGGTAACCGACACTTCAACCTTGCTGTTGGTATCAAGC
CCGGCACGCTCTCAAACGGATTGAAGTATTCGCTTGCCACAGGAAACTGGGGTGATCA
GAAGAAGGCCATGAGCTCGACTGCAGGTGTGTCACAGGTGCTTAACCGTTACACGTTT
GCTTCGACCTTGTCACATTTGCGTCGTACCAATACTCCTATCGGAAGAGATGGTAAGCT
GGCAAAGCCTCGACAGCTTCACAACACGCATTGGGGTTTGGTCTGTCCTGCCGAGACA
CCCGAAGGACAGGCCTGTGGTCTGGTCAAGAACTTGTCTTTGATGTGTTACGTCAGTG
TCGGTTCTCCCTCCGAGCCTCTGATTGAATTCATGATCAACAGAGGTATGGAGGTCGTA
GAAGAGTATGAGCCGCTGCGGTATCCTCATGCTACAAAGATTTTTGTGAACGGTGTCTG
GGTTGGAGTTCACCAAGACCCTAAGCACTTGGTGAACCAGGTTCTGGACACTCGTCGC
AAGTCCTATCTGCAGTACGAAGTCTCTCTCGTGAGAGAAATTCGAGACCAGGAATTCA
AAATCTTTTCCGACGCAGGCCGTGTCATGCGACCAGTCTTTACCGTTCAGCAGGAAGA
TGACCCGGAAACGGGCATCAACAAGGGTCACCTGGTATTGACCAAGGAGCTCGTCAAT
AGATTGGCCAAGGAGCAGGCTGAGCCTCCGGAAGATCCCAGCATGAAGATCGGATGG
GAGGGATTAATCAGGGCTGGTGCGGTTGAATATCTCGACGCCGAGGAAGAGGAGACG
TCCATGATCTGCATGACGCCAGAGGATCTCGAGCTGTATCGTCTTCAGAAGGCCGGTAT
TGACACTGAGGAAGACATGGGAGATGACCCGAACAAGCGACTAAAGACCAAGACAA
ACCCGACAACTCACATGTACACCCATTGCGAGATTCACCCAAGTATGATCTTAGGCATC
TGTGCTAGTA
