# Supplementary material for: Genomic Characterization and Establishment of a Genetic Manipulation System for Trichoderma sp. (Harzianum Clade) LZ117
Source: J Fungi (Basel). 2024 Oct 7;10(10):697. doi: 10.3390/jof10100697 (PMC11508783; doi:10.3390/jof10100697)
Supplement: Supplementary file 1 [file jof-10-00697-s001.zip › Supplementary Materials S2.pdf]

>*Trichoderma sp.* LZ117
GGGGGGGGGAGGTGCACTCTCACGTGTCGTTTCGTACGTACAATCCTGTTCCCTCACG
TCGGCATCATTTGCCGGTCTGATTCTCAAACACTTGTGCTAACCATCGCCTTCTAGGGG
TGCGTATTCCATCAATCATCTTGAATGAGATCGATCGAACACAATACTGACTTGCTATAA
CAGCCACGTCGACTCCGGAAAGTCGACCACCGTAAGTTGCTCCCTCTTCTTTGCTCC
GACATCAAACGCCGTTTGATGCGGGACATCTCTTGAACACAGGGCTAACCATTCATCAT
ACAGACCGGTCACTTGATCTACCAGTGCGGTGGTATCGACCGTCGTACCATCGAGAAG
TTCGAGAAGGTAAGCTTCAACTGATTTCGCCTCGATTCTTCCTCCTTCACATTCAATTG
TGCCCGACAATTCTGCAGAGAATTTTGGTGTCGACAATTTTTCATCACCCCGCTTTCCA
TTACCCCTCCTTTGCAGCGACGCAAATTTTTTTTGCTGCCGTTTGATTTTTAGTGGGGTT
CTTTGTGCAACCCCACTAGCTCACTGCTTTTTTTTGTGCTTCACTCTCACTTCCCATCCA
TCATTCAACGTGCTCTGTGTCTTTGGTCATTCAGCGATGCTAACCACTTTTTCCATCAAT
AGGAAGCCGCCGAACTCGGCAAGGGTTCCTTCAAGTACGCTTGGGTTCTTGACAAGCT
CAAGGCCGAGCGTGAGCGTGGTATCACCATCGACATTGCTCTGTGGAAGTTCGAGACT
CCCAAGTACTATGTCACCGTCATTGGTATGCCTTCATCAATCTCATGGTGCAACTGCGA
GCTAGTGCTAACATGCAATTCACAGACGCTCCCGGCCACCGTGATTTCATCAAGAACAT
ATAGGGGGACCC

>KP292611.1 *Trichoderma guizhouense* NJAU 4742
ACGTGGTCGTTATCGTACGTATCATCCTTTCTCACGTCGGCATCATTCGCCGGTCTGATT
CTCAGACTTGTGCTAACCATCGCCTTCTAGGGGTGCGTATTCCATCAATCATCTTGAATG
AGATCGATCGAACACAGTACTGACTTGCTACAACAGCCACGTCGACTCCGGAAAGTCG
ACCACCGTAAGTTACACCCTCTCTTGCTCCGATATCAAACGTCGTTTGATGCGGGACAT
CTACTCTTGAACACAGGGCTAACCATTCATCATACAGACCGGTCACTTGATCTACCAGT
GCGGTGGTATCGACCGTCGTACCATCGAGAAGTTCGAGAAGGTAAGCGTCAACTGATT
TTCGCCTCGATTCCTCTTCTTTCATATTCAATTGTGCCCGACAATTCTTCAGAGACTTTT
TGGGTCGACAATTTTTCGTCACCCCGCTTTCCATTACCCCTCCTTTGCAGCGACGCAAA
TTTTTTTTGCTGCCGTTTGATTTTTAGTGGGGTTCTCTGTGCAACCCCACTAGCTCACTG
CTTTTTTTGTGCTTCATTCACTTCCCAGTCATCATTCAACGTGCTCTGTGTCTTTGGTTAT
TCAACGATGCTAACCACTTTTCCATCAATAGGAAGCCGCCGAACTCGGCAAGGGTTCC
TTCAAGTACGCTTGGGTTCTTGACAAGCTCAAGGCCGAGCGTGAGCGTGGTATCACCA
TCGACATTGCTCTGTGGAAGTTCGAGACTCCCAAGTACTATGTCACCGTCATTGGTATG
TCTACTTCATCAACTTCATGTATGCAATTGCAACCCAGTGCTAACAGGCAATCACAGAC
GC

>KU238063.1 Trichoderma guizhouense TS73
CTCACTTCGGCATCGTTCGCCGGTCTGATTCTCAAACTTGTGCTAACCATCGCCTTCTA
GGGGTGCGTATTCCATCAATCATCTTGAATGAGATCGATCGAACACAATACTGACTTGC
TACAACAGCCACGTCGACTCCGGAAAGTCGACCACCGTAAGTTACACCCTCTCTTGCT
CCGATATCAAACGTCGTTTGATGCGGGACATCTACTCTTGAACTCAGGGCTAACCATGC
ATCATACAGACCGGTCACTTGATCTACCAGTGCGGTGGTATCGACCGTCGTACCATCGA
GAAGTTCGAGAAGGTAAGCTTCAACTGATTTTCGCCTCGATTCCTCTGCTTTCAAATTC
AATTGTGCCCGACAATTCTTCAGAGAATTTTCGTGTCGACAGTTTTTCATCACCCCGCC
TTCCATTACCCCTCCTTTGCAGCGACGCAAATTTTTTTTGCTACCGTTTGATTTTTAGT

GGGGTTCTCTGTGCACAACCCCACTAGCTCACTGCTTTTTTTCTTTGCTTCACTCACTT
CCCAGTTATCATTCAACGTGCTCTGTGTCTTTGATCATTCAACGATGCTAACCACTTTTC
CATCAATAGGAAGCCGCCGAACTCGGCAAGGGTTCCTTCAAGTACGCTTGGGTTCTTG
ACAAGCTCAAGGCCGAGCGTGAGCGTGGTATCACCATCGACATTGCTCTGTGGAAGTT
CGAGACTCCCAAGTACTATGTCACCGTCATTGGTATGTCTACTTCATCAACTTCATGCTG
CAATTGCAACCCAGTGCTAACAGGCAATTCACA

>AY937440.1 Trichoderma guizhouense GJS 97-28
GAAGGTAAGCTTCAACTGATTTTCGCCTCGATTCCTCTTTCATATTCAATTGTGCCCGAC
AATTCTTCAGAGAATTTTCGTGTCGACAATTTTTCATCACCCCGCTTTCCATTACCCCTC
CTTTGCAGCGACGCAAATTTTTTTTGCTGCCGTTTGATTTTTAGTGGGGTTCTCTGTGCA
ACCCCACTATCTCACTGCTTTTTTTTGTGCTTCACTCACTTCCCAGTCATTCAACGTGCT
CTGTGTCTTTGGTCATTCAACGATGCTAACCACTTTTCCATCAATAGGAAGCCGCCGAA
CTCGGCAAGGGTTCCTTCAAGTACGCTTGGGTTCTTGACAAGCTCAAGGCCGAGCGTG
AGCGTGGTATCACCATCGACATTGCTCTGTGGAAGTTCGAGACTCCCAAGTACTATGTC
ACCGTCATTGGTATGTCTACTTATCAACTTCATGCTGCAATTGCAACCCAGTGCTAACA
GGCAATTCACAGACGCTCCCGGCCACCGTGATTTCATCAAGAACAT

>KU738448.1 Trichoderma harzianum ER074
ACGTGGTCGTTATCGTACGTATCATCCTGTTCCCTCACGTCGGCATCATTCGCCGCTCTG
ATTCCAAACGCTTGTGCTAACCATCGACTTCTAGGGGTGCGTATTCCATCAATTATCTTG
AATGAGATCGACCGAACACAATACTGACTTGCTACAACAGCCACGTCGACTCCGGAAA
GTCGACCACCGTAAGTTGCACCCTCTCTTGCTCCGATATCAAACGTCGTTTAATGGGGA
CACATACTCTTGAACACAGGGCTAACCATTCATCATACAGACCGGTCACTTGATCTACC
AGTGCGGTGGTATCGACCGTCGTACCATCGAGAAGTTCGAGAAGGTAAGCTTCAACTG
ATTTTCGCCTCGATTCTTCCTCCTTCACATTCAATTGTGCCCGACAATTCTGCAGAGAAT
TTTCGTGTCGACAATTTTTCATCACCCCGCTTTCCATTACCCCTCCTTTGCAGCGACGCA
AATTTTTTTGCTGTCGTTTGGTTTTTAGTGGGGTTCTCTGTGCAACCCCACTAGCTCACT
GCTTTTTCCTGCTTCACTCTCACTTCCTCATCATCATTCAACACGCTCTGTGTCTTTGGT
CATTCAGCGATGCTAACCACTTTTCCATCAATAGGAAGCCGCCGAACTCGGCAAGGGT
TCCTTCAAGTACGCTTGGGTTCTTGACAAGCTCAAGGCCGAGCGTGAGCGTGGTATCA
CCATCGACATTGCTCTGTGGAAGTTCGAGACTCCCAAGTACTATGTCACCGTCATTGGT
ATGCCTTCATCAATCTCATGGTGCAACTGCGAGCTAGTGCTAACATGCAATTCACAGAC
GCTCCCGGCCACCGTGATTTCATCAAGAAC

>MN927126.1 Trichoderma harzianum AT73-2
ACGTGGTCGTTATCGTACGTACAATCCTGTTCCCTCACGTCGGCATCATTCGCCGGTCT
GATTCTCAAACACTTGTGCTAACCATTGCCTTCTAGGGGTGCGTATTCCATCAATCATCT
TGAATGAGATCGATCGAACACAATACTGACTTGCTATAACAGCCACGTCGACTCCGGA
AAGTCGACCACCGTAAGTTGCGCCCTCTCTTGCTCCGATATCAAACATCGTTTGATGCG
GGACACCTATTCTTGAACACAGGGCTAACCATGCATAATACAGACCGGTCACTTGATCT
ACCAGTGCGGTGGTATCGACCGTCGTACCATCGAGAAGTTCGAGAAGGTAAGCTTCAA
CTGATTTTCGCCTCGATTCTTCCTCTCTCCACATTCAATTGTGCCCGACAATTCTGCAGA
GAATTTTCGTGTCGACAATTTTTCATCACCCCGCTTTCCATTACCCCTCCTTTGCAGCGA

CGCAAATTTTTTTGCTGTCGTTTGGTTTTAGTGGGGTTCTCTGTGCAACCCCACTAG
CTCCCTGCTTTTTCCTGCTTCACTCTCACTTCCTCGTCATCATTCAACGTGCTCTGCGTC
TTTGGTCATTCAGCGACGCTAACCACTTTTACATCAATAGGAAGCCGCCGAACTCGGCA
AGGGTTCCTTCAAGTACGCTTGGGTTCTTGACAAGCTCAAGGCCGAGCGTGAGCGTGG
TATCACCATTGACATTGCTCTGTGGAAGTTCGAGACTCCCAAGTACTATGTCACCGTCA
TTGGTAAGTCTTCACTAAGTTCATGCTGCAATTGCGGACCAGTGCTAACAGGCAATTCA
CAGACGCTCCCGGCCACCGTGATTTCATCAAGAAC

>AF348099.1 Trichoderma inhamatum CBS 273.78
CTACCAGTGCGGTGGTATCGACCGTCGTACCATCGAGAAGTTCGAGAAGGTAAGCTTC
AACTGATTTTCGCCTCGATCCTCCCTCTACATTCAATTGAACCCGACAATTCTGAAGAG
AATTTTCGTGTTCGACAATTTTTCATCACCCCGCTTTCCATTACCCCTCCTTTGCAGCGA
CGCAAATTTTTTTGCTGTCTGTTGGTTTTAGTGGGGTTTCTTGTGCACCCCACTAGCTC
ACCTGTATTTTTCTGCTTCACTCACTTCCCAGCCATCATTCAGCGTGTTCTGTGTCCTTG
GTCATTCAGCGATGCTAACCACTTTTCCATCAATAGGAAGCCGCCGAACTCGGCAAGG
GTTCCTTCAAGTACGCTTGGGTTCTTGACAAGCTCAAGGCCGAGCGTGAGCGTGGTAT
CACCATCGACATTGCTCTGTGGAAGTTCGAGACTCCCAAGTACTATGTCACCGTCATTG
GTATGTCTTCTTCATTAACTTCATGCTTCAATTGCAAGTCAGTGCTAACAGGCAATTCAC
AGACGCTCCCGGCCACCGTGATTTCATCAAGAACAT

>FJ463347.1 Trichoderma lentiforme DIS 173F
GAGAAGGTAAGCTTCAACTGATTTTCGCCTCGATTCTCCCTCCTCCAAATTCAATTGTG
CCCGACGATTCTGAAGAGAATTTTCGTGTCGACAATTTTTCGTCACCCCGCTTTCCATT
ACCCCTCCTTTGCAGCGACGCAAATTTTTTTGCTGTCTTTTGGTTTTAGTGGGGTTTCTT
GTGCACCCCACTAGCTCACTGCTTTTTTTTTTTTTGGCTTCACTCCCACTTCCCCGCCAT
TCAACGTACTCTGTGTCTTTGGTCATTCAGCGATGCTAACCACTTTTCCATCAATAGGA
AGCCGCCGAACTCGGCAAGGGTTCCTTCAAGTACGCTTGGGTTCTTGACAAGCTCAAG
GCCGAGCGTGAGCGTGGTATCACCATCGACATTGCTCTGTGGAAGTTCGAGACTCCCA
AGTACTATGTCACCGTCATTGGTATGTCTTCTTCATCAACTTCATGCTTCAATTGCAAGC
CAGTGCTAACAGGCAATTCACAGACGCTCCCGGCCACCGTGATTTCATCAAGAACAT

>FJ463310.1 Trichoderma lentiforme DIS 218E
CTTCAACTGATTTTCGCCTCGATTCTCCCTCCTCCAAATTCAATTGTGCCCGACGATTCT
GAAGAGAATTTTCGTGTCGACAATTTTTCGTCACCCCGCTTTCCATTACCCCTCCTTTGC
AGCGACGCAAATTTTTTTGCTGTCTTTTGGTTTTAGTGGGGTTTCTTGTGCACCCCACT
AGCTCACTGCTTTTTTTTTTTTTGGCTTCACTCTCACTTCCCCGCCATTCAACGTACTCTG
TGTCTTTGGTCATTCAGCGATGCTAACCACTTTTCCGTCAATAGGAAGCCGCCGAACTC
GGCAAGGGTTCCTTCAAGTACGCTTGGGTTCTTGACAAGCTCAAGGCCGAGCGTGAG
CGTGGTATCACCATCGACATTGCTCTGTGGAAGTTCGAGACTCCCAAGTACTATGTCAC
CGTCATTGGTATGTCTTCTTCATCAACTTCATGCTTCAATTGCAAGCCAGTGCTAACAG
GCAATTCACAGACGCTCCCGGCCACCGTGATTTCATCAAGAACAT

>FJ463346.1 Trichoderma lentiforme DIS 246J
TTCACTGATTTTCGCCTCGATTCTCCCTTCTCCAAATTCAATTGTGCCCGACGATTCTGA

AGAGAATTTTCGTGTCGACAATTTTTCGTCACCCCGCTTTCCATTACCCCTCCTTTGCAG
CGACGCAAATTTTTTTGCTGTCTTTTGGTTTTAGTGGGGTTTCTTGTGCACCCCACTAG
CTCACTGCTTTTTTTTTTTTGGCTTCACTCTCACTTCCCCGCCATTCAACGTACTCTGTG
TCTTTGGTCATTCAGCGATGCTAACCACTTTTCCGTCAATAGGAAGCCGCCGAACTCGG
CAAGGGTTCCTTCAAGTACGCTTGGGTTCTTGACAAGCTCAAGGCCGAGCGTGAGCGT
GGTATCACCATCGACATTGCTCTGTGGAAGTTCGAGACTCCCAAGTACTATGTCACCGT
CATTGGTATGTCTTCTTCATCAACTTCATGCTTCAATTGCAAGCCAGTGCTAACAGGCA
ATTCACAGACGCTCCCGGCCACCGGATCATCAAG

>AF348093.1 Trichoderma afarasin GJS 99-227
CTACCAGTGCGGTGGTATCGACCGTCGTACCATCGAGAAGTTCGAGAAGGTAAGCTTC
AACTCATTTTCACCTCAACTCTCCCTCCACATTCAATTGTGCCCGACAATTCTGCAGAG
AATTTTCGTGTCGACAATTTTTCATCACCCCGCTTTGCATTACCCCTCCTTTGCAGCGA
CGCAAATTTTTTTGCTGTTGTTTGGTTTTAGTGGGGTTTCTTGTGCACCCCCACTAACT
CACTGCTTTGTTTTCTGCTTCGCTCTCACTTCCCAGCCATCATTCAACGTGCTCTGTGTC
TCGTCACTTTCAGCGATGCTAACCGCTTTTCTATCAATAGGAAGCCGCCGAACTCGGCA
AGGGTTCCTTCAAGTACGCTTGGGTTCTTGACAAGCTCAAGGCCGAGCGTGAGCGTGG
TATCACCATCGACATTGCTCTGTGGAAGTTCGAGACTCCCAAGTACTATGTCACCGTCA
TTGGTATGTTCTTTCCATCAACTTCACACAGCGATTACAAGCCAGTGCTAACAAGCAAT
TCACAGACGCTCCCGGCCACCGTGATTTCATCAAGAACAT

>FJ463327.1 Trichoderma afarasin GJS 06-98
GAGAAGGTAAGCTTCAACTCATTTTCACCTCAATTCTCCCTCCACATTCAATTGTGCCC
GACAATTCTGCAGAGAATTTTGATGTCGACAATTTTTCATCACCCCGCTTTGCATTACCC
CTCCTTTGCAGCGACGCAAAATTTTTTTGCTGTCGTTTGGTTTTAGTGGGGTTTCTTGT
GCACCCCCACTAGCTCACTGCTTTTTTTCCTGCTTCGCTCTCACTTCCCGGCCATCATTC
AACGTGCTCTGTGTCTCGTCACTTTCAGCGATGCTAACCGCTTTTCTATCAATAGGAAG
CCGCCGAACTCGGCAAGGGTTCCTTCAAGTACGCTTGGGTTCTTGACAAGCTCAAGGC
CGAGCGTGAGCGTGGTATCACCATCGACATTGCTCTGTGGAAGTTCGAGACTCCCAAG
TACTATGTCACCGTCATTGGTATGTTCTTTCCATCAACTTCACACAGCGATTACAAGCCA
GTGCTAACAAGCAATTCACAGACGCTCCCGGCCACCGTGATTTCATCAAGAACAT

>FJ463400.1 Trichoderma afarasin DIS 314F
AGAAGGTAAGCTTCAACTCATTTTCACCTCAATTCTCCCTCCACATTCAATTGTGCCCG
ACAATTCTGCAGAGAATTTTGGTGTCGACAATTTTTCATCACCCCGCTTTGCATTACCCC
TCCTTTGCAGCGACGCAAAATTTTTTTGCTGTCGTTTGGTTTTAGTGGGGTTTCTTGTG
CACCCCCACTAGCTCACTGCTTTTTTTCCTGCTTGGCTCTCACTTCCCGGCCATCATTCA
ACGTGCTCTGTGTCTCGTCACTTTCAGCGATGCTAACCGCTTTTCTATCAATAGGAAGC
CGCCGAACTCGGCAAGGGTTCCTTCAAGTACGCTTGGGTTCTTGACAAGCTCAAGGCC
GAGCGTGAGCGTGGTATCACCATCGACATTGCTCTGTGGAAGTTCGAGACTCCCAAGT
ACTATGTCACCGTCATTGGTATGTTCTTTCTATCAACTTCACACAGCGATTACAAGCCAG
TGCTAACAAGCAATTCACAGACGCTCCCGGCCACCGTGATTTCATCAAGAACAT

>FJ463299.1 Trichoderma atrobrunneum GJS 05-100

AGGTAAGCTTCAACTGATTTTCGCCTCGATTCTTCCTCCTTCACATTCAATTGTGCCCGA
CAATTCTGCAGAGAATTTTCGTGTCGACAATTTTTCATCACCCCGCTTTCCATTACCCCT
CCTTTGCAGCGACGCAAATTTTTTTGCTGTCGTTTGGTTTTTAGTGGGGTTCTCTGTGC
AACCCCACTAGCTCACTGCTTTTTCCTGCTTCACTCTCACTTCCTCATCATCATTCAACA
CGCTCTGTGTCTTTGGTCATTCAGCGATGCTAACCACTTTTCCATCAATAGGAAGCCGC
CGAACTCGGCAAGGGTTCCTTCAAGTACGCTTGGGTTCTTGACAAGCTCAAGGCCGAG
CGTGAGCGTGGTATCACCATCGACATTGCTCTGTGGAAGTTCGAGACTCCCAAGTACTA
TGTCACCGTCATTGGTATGCCTTCATCAATCTCATGGTGCAACTGCGAGCTAGTGCTAA
CATGCAATTCACAGACGCTCCCGGCCACCGTGATTTCATCAAGAACAT

>FJ463360.1 Trichoderma atrobrunneum GJS 04-67
GAGAAGGTAAGCTTCAACTGATTTTCGCCTCGATTCTTCCTCCTTCACATTCAATTGTG
CCCGACAATTCTGCAGAGAATTTTCGTGTCGACAATTTTTCATCACCCCGCTTTCCGTT
ACCCCTCCTTTGCAGCGACGCAAAATTTTTTTGCTGTCGTCTGGTTTTTAGTGGGGTT
CTCTGTGCAACCCCACTAGCTCACTGCTTTTTCCTGCTTCACTCTCACTTCCTCGTCATC
ATTCAACACGCTCTGTGTCTTTGGTCATTCAGCGATGCTAACCACTTTTCCATCAATAGG
AAGCCGCCGAACTCGGCAAGGGTTCCTTCAAGTACGCTTGGGTTCTTGACAAGCTCAA
GGCCGAGCGTGAGCGTGGTATCACCATCGACATTGCTCTGTGGAAGTTCGAGACTCCC
AAGTACTATGTCACCGTCATTGGTATGTCTTCATCAATCTCATGGTGCAACTGCGAGCTA
GTGCTAACATGCAATTCACAGACGCTCCCGGCCACCGTGATTTCATCAAGAACAT

>FJ463304.1 Trichoderma atrobrunneum GJS 05-469
GAGAAGGTAAGCTTCAACTGATTTTCGCCTCGATTCTTCCTCCTTCACATTCAATTGTG
CCCGACAATTCTGCAGAGAATTTTCGTGTCGACAATTTTTCATCACCCCGCTTTCCATTA
CCCCTCCTTTGCAGCGACGCAAAATTTTTTTGCTGTCGTTTGGTTTTTAGTGGGGTTCT
CTGTGCAACCCCACTAGCTCACTGCTTTTTCCTGCTTCACTCTCACTTCCTAGTCATCAT
TCAACACGCTTTGTGGCTTTGGTCATTCAGCGATGCTAACCACTTTTCCATCAATAGGA
AGCCGCCGAACTCGGCAAGGGTTCCTTCAAGTACGCTTGGGTTCTTGACAAGCTCAAG
GCCGAGCGTGAGCGTGGTATCACCATCGACATTGCTCTGTGGAAGTTCGAGACTCCCA
AGTACTATGTCACCGTCATTGGTATGTCCTCATCAATCTCATGGTGCAACTGCGAGCTAG
TGCTAACATGCAATTCACAGACGCTCCCGGCCACCGTGATTTCATCAAGAACAT

>FJ463303.1 Trichoderma atrobrunneum GJS 05-467
GAGAAGGTAAGCTTCAACTGATTTTCGCCTCGATTCTTCCTCCTTCACATTCAATTGTG
CCCGACAATTCTGCAGAGAATTTTCGTGTCGACAATTTTTCATCACCCCGCTTTCCATTA
CCCCTCCTTTGCAGCGACGCAAAATTTTTTTGCTGTCGTTTGGTTTTTAGTGGGGTTCT
CTGTGCAACCCCACTAGCTCACTGCTTTTTCCTGCTTCACTCTCACTTCCTAGTCATCAT
TCAACACGCTTTGTGGCTTTGGTCATTCAGCGATGCTAACCACTTTTCCATCAATAGGA
AGCCGCCGAACTCGGCAAGGGTTCCTTCAAGTACGCTTGGGTTCTTGACAAGCTCAAG
GCCGAGCGTGAGCGTGGTATCACCATCGACATTGCTCTGTGGAAGTTCGAGACTCCCA
AGTACTATGTCACCGTCATTGGTATGTCCTCATCAATCTCATGGTGCAACTGCGAGCTAG
TGCTAACATGCAATTCACAGACGCTCCCGGCCACCGTGATTTCATCAAGAACAT

>FJ463392.1 Trichoderma atrobrunneum GJS 05-101

GAGAAGGTAAGCTTCAACTGATTTTCGCCTCGATTCTTCCTCCTTCACATTCAATTGTG
CCCGACAATTCTGCAGAGAATTTTCGTGTCGACAATTTTTCATCACCCCGCTTTCCATTA
CCCCTCCTTTGCAGCGACGCAAAAAATTTTTTGCTGTCGTTTGGTTTTAGTGGGGTTC
TCTGTGCAACCCCACTAGCTCACTGCTTTTTCCTGCTTCACTCTCACTTCCTAGTCATCA
TTCAACACGCTTTGTGGCTTTGGTCATTCAGCGATGCTAACCACTTTTCCATCAATAGG
AAGCCGCCGAACTCGGCAAGGGTTCCTTCAAGTACGCTTGGGTTCTTGACAAGCTCAA
GGCCGAGCGTGAGCGTGGTATCACCATCGACATTGCTCTGTGGAAGTTCGAGACTCCC
AAGTACTATGTCACCGTCATTGGTATGTCCTCATCAATCTCATGGTGCAACTGCGAGCTA
GTGCTAACATGCAATTCACAGACGCTCCCGGCCACCGTGATTTCATCAAGAACAT

>FJ463297.1 Trichoderma atrobrunneum GJS 05-106
AAGGTAAGCTTCAACTGATTTTCGCCTCGATTCTTCCTCCTTCACATTCAATTGTGCCCG
ACAATTCTGCAGAGAATTTTCGTGTCGACAATTTTTCATCACCCCGCTTTCCATTACCCC
TCCTTTGCAGCGACGCAAAAAATTTTTTGCTGTCGTTTGGTTTTAGTGGGGTTCTCTG
TGCAACCCCACTAGCTCACTGCTTTTTCCTGCTTCACTCTCACTTCCTAGTCATCATTCA
ACACGCTTTGTGGCTTTGGTCATTCAGCGATGCTAACCACTTTTCCATCAATAGGAAGC
CGCCGAACTCGGCAAGGGTTCCTTCAAGTACGCTTGGGTTCTTGACAAGCTCAAGGCC
GAGCGTGAGCGTGGTATCACCATCGACATTGCTCTGTGGAAGTTCGAGACTCCCAAGT
ACTATGTCACCGTCATTGGTATGTCCTCATCAATCTCATGGTGCAACTGCGAGCTAGTGC
TAACATGCAATTCACAGACGCTCCCGGCCACCGTGATTTCATCAAGAACAT

>AF348108.1 Trichoderma camerunense GJS 99-231
CTACCAGTGCGGTGGTATCGACCGTCGTACCATCGAGAAGTTCGAGAAGGTAAGCTTC
AACTTATTTTCGCCTCGATTCTCCCTTCACATTCAATTGTGCCCGACAATTCTGCAGAGA
ATTTTCTTGTCAACAATTTTTCATCACCCCGCTTTGCATTACCCCTCCTTTGCAGCGACG
CAAATTTTTTTGCTGTCGTTTGGTTTTAGTGGGGTTTCTTGTGCACCCCACTAGCTCAC
TACTTTTTTTCTGCTTCGCTCTCACTTCCCAGCCATCATTCAACGTGCTCCGTGTCATCA
CTTTCAGCGATGCTAACCACTTTTCCATCAATAGGAAGCCGCCGAACTCGGCAAGGGT
TCCTTCAAGTACGCTTGGGTTCTTGACAAGCTCAAGGCCGAGCGTGAGCGTGGTATCA
CCATCGACATTGCTCTGTGGAAGTTCGAGACTCCCAAGTACTATGTCACCGTCATTGGT
ATGTTCTTTCCATCAACTTCACACAGCGATCACAAGCCAGTGCTAACAAGCAATTCACA
GACGCTCCCGGCCACCGTGATTTCATCAAGAACAT

>AF348107.1 Trichoderma camerunense GJS 99-230
CTACCAGTGCGGTGGTATCGACCGTCGTACCATCGAGAAGTTCGAGAAGGTAAGCTTC
AACTCATTTTCGCCTCGATTCTCCCTTCACATTCAATTGTGCCCGACAATTCTGCAGAG
AATTTTCTTGTCAACAATTTTTCATCACCCCGCTTTGCATTACCCCTCCTTTGCAGCGAC
GCAAATTTTTTTGCTGTCGTTTGGTTTTAGTGGGGTTTCTTGTGCACCCCACTAGCTCA
CTACTTTTTTTCTGCTTCGCTCTCACTTCCCAGCCATCATTCAACGTGCTCCGTGTCATC
ACTTTCAGCGATGCTAACCACTTTTCCATCAATAGGAAGCCGCCGAACTCGGCAAGGG
TTCCTTCAAGTACGCTTGGGTTCTTGACAAGCTCAAGGCCGAGCGTGAGCGTGGTATC
ACCATCGACATTGCTCTGTGGAAGTTCGAGACTCCCAAGTACTATGTCACCGTCATTGG
TATGTTCTTTCCATCAACTTCACACAGCGATCACAAGCCAGTGCTAACAAGCAATTCAC
AGACGCTCCCGGCCACCGTGATTTCATCAAGAACAT

>MH371381.1 Trichoderma endophyticum GJS 08-127
GAGAAGGTAAGCTTCAACTCATTTTCACCTCAACTCTCCCTCCACATTCAATTGTGCCC
GACAATTCTGCAGAGAATTTTCGTGTCGACAATTTTTTCATCACCCCGCTTTGCATTACC
CCTCCTTTGCAGCGACGCAAAATTTTTTTTGCTGTTGTTTGGTTTTAGTGGGGTTTCTTG
TGCACCCCCACTAACTCACTGCTTTTTTTTCTGCTTCGCTCTCACTTCCCAGCCATCATT
CAACGTGCTCTGTGTCTCGTCACTTTCAGCGATGCTAACCGCTTTTCTATCAATAGGAA
GCCGCCGAACTCGGCAAGGGTTCCTTCAAGTACGCTTGGGTTCTTGACAAGCTCAAGG
CCGAGCGTGAGCGTGGTATCACCATCGACATTGCTCTGTGGAAGTTCGAGACTCCCAA
GTACTATGTCACCGTCATTGGTATGTTCTTTCCATCAACTTCACACAGCGATTACAAGCC
AGTGCTAACAAGCAATTCACAGACGCTCCCGGCCACCGTGATTTCATCAAGAACAT

>MH371384.1:1-531 Trichoderma endophyticum GJS 08-184
GAGAAGGTAAGCTTCAACTCATTTTCACCTCAACTCTCCCTCCACATTCAATTGTGCCC
GACAATTCTGCAGAGAATTTTCGTGTCGACAATTTTTTCATCACCCCGCTTTGCATTACC
CCTCCTTTGCAGCGACGCAAAAATTTTTTTGCTGTTGTTTGGTTTTAGTGGGGTTTCTT
GTGCACCCCCACTAACTCACTGCTTTTTTTTCTGCTTCGCTCTCACTTCCCAGCCATCAT
TCAACGTGCTCTGTGTCTCGTCACTTTCAGCGATGCTAACCGCTTTTCTATCAATAGGA
AGCCGCCGAACTCGGCAAGGGTTCCTTCAAGTACGCTTGGGTTCTTGACAAGCTCAAG
GCCGAGCGTGAGCGTGGTATCACCATCGACATTGCTCTGTGGAAGTTCGAGACTCCCA
AGTACTATGTCACCGTCATTGGTATGTTCTTTCCATCAACTTCACACAGCGATTACAAGC
CAGTGCTAACAAGCAATTCACAGACGCTCCCGGCCACCGTGATTTCATCAAGAACAT

>MH371383.1:1-531 Trichoderma endophyticum GJS 08-177
GAGAAGGTAAGCTTCAACTCATTTTCACCTCAACTCTCCCTCCACATTCAATTGTGCCC
GACAATTCTGCAGAGAATTTTCGTGTCGACAATTTTTTCATCACCCCGCTTTGCATTACC
CCTCCTTTGCAGCGACGCAAAAATTTTTTTGCTGTTGTTTGGTTTTAGTGGGGTTTCTT
GTGCACCCCCACTAACTCACTGCTTTTTTTTCTGCTTCGCTCTCACTTCCCAGCCATCAT
TCAACGTGCTCTGTGTCTCGTCACTTTCAGCGATGCTAACCGCTTTTCTATCAATAGGA
AGCCGCCGAACTCGGCAAGGGTTCCTTCAAGTACGCTTGGGTTCTTGACAAGCTCAAG
GCCGAGCGTGAGCGTGGTATCACCATCGACATTGCTCTGTGGAAGTTCGAGACTCCCA
AGTACTATGTCACCGTCATTGGTATGTTCTTTCCATCAACTTCACACAGCGATTACAAGC
CAGTGCTAACAAGCAATTCACAGACGCTCCCGGCCACCGTGATTTCATCAAGAACAT

>MH371382.1:1-531 Trichoderma endophyticum GJS 08-175
GAGAAGGTAAGCTTCAACTCATTTTCACCTCAACTCTCCCTCCACATTCAATTGTGCCC
GACAATTCTGCAGAGAATTTTCGTGTCGACAATTTTTTCATCACCCCGCTTTGCATTACC
CCTCCTTTGCAGCGACGCAAAAATTTTTTTGCTGTTGTTTGGTTTTAGTGGGGTTTCTT
GTGCACCCCCACTAACTCACTGCTTTTTTTTCTGCTTCGCTCTCACTTCCCAGCCATCAT
TCAACGTGCTCTGTGTCTCGTCACTTTCAGCGATGCTAACCGCTTTTCTATCAATAGGA
AGCCGCCGAACTCGGCAAGGGTTCCTTCAAGTACGCTTGGGTTCTTGACAAGCTCAAG
GCCGAGCGTGAGCGTGGTATCACCATCGACATTGCTCTGTGGAAGTTCGAGACTCCCA
AGTACTATGTCACCGTCATTGGTATGTTCTTTCCATCAACTTCACACAGCGATTACAAGC
CAGTGCTAACAAGCAATTCACAGACGCTCCCGGCCACCGTGATTTCATCAAGAACAT

>MG822718.1 Trichoderma neotropicale GJS 08-182
GAGAAGGTAAGCTTCAACTCATTTTCACCTCAACTCTCCCTCCACATTCAATTGTGCCC
GACAATTCTGCAGAGAATTTTCGTGTCGAGAATTTTTTCATCACCCCGCTTTGCATTACC
CCTCCTTTGCAGCGACGCAAAATTTTTTTGCTGTTGTTTGGTTTTAGTGGGGTTTCTTGT
GCACCCCCACTAACTCACTGCTTTTTTTTCTGCTTCGCTCTCACTTCCCAGCCATCATTC
AACGTGCTCTGTGTCTCGTCACTTTCAGCGATGCTAACCGCTTTTCTATCAATAGGAAG
CCGCCGAACTCGGCAAGGGTTCCTTCAAGTACGCTTGGGTTCTTGACAAGCTCAAGGC
CGAGCGTGAGCGTGGTATCACCATCGACATTGCTCTGTGGAAGTTCGAGACTCCCAAG
TACTATGTCACCGTCATTGGTATGTTCTTTCCATCAACTTCACACAGCGATTACAAGCCA
GTGCTAACAAGCAATTCACAGACGCTCCCGGCCACCGTGATTTCATCAAGAACAT

>MG822719.1 Trichoderma neotropicale GJS 08-183
GTAAGCTTCAACTCATTTTCACCTCAACTCTCCCTCCACATTCAATTGTGCCCGACAATT
CTGCAGAGAATTTTCGTGTCGACAATTTTTTCATCACCCCGCTTTGCATTACCCCTCCTT
TGCAGCGACGCAAAATTTTTTTGCTGTTGTTTGGTTTTAGTGGGGTTTCTTGTGCACC
CCCACTAACTCACTGCTTTTTTTTCTGCTTCGCTCTCACTTCCCAGCCATCATTCAACGT
GCTCTGTGTCTCGTCACTTTCAGCGATGCTAACCGCTTTTCTATCAATAGGAAGCCGCC
GAACTCGGCAAGGGTTCCTTCAAGTACGCTTGGGTTCTTGACAAGCTCAAGGCCGAGC
GTGAGCGTGGTATCACCATCGACATTGCTCTGTGGAAGTTCGAGACTCCCAAGTACTAT
GTCACCGTCATTGGTATGTTCTTTCCATCAACTTCACACAGCGATTACAAGCCAGTGCT
AACAAGCAATTCACAGACGCTCCCGGCCACCGTGATTTCATCAAGAACAT

>MZ927309.1 Trichoderma rifaii NN37
GTTATCGTACGTATTATCCCTTCTTCACGTCGGCATCAGTCGCCACTCTGATTCTCAAAC
ACTTGTGCTAACTACCATCTTCTAGGGGTGCGTATTCCATCAATCATCTTGAATGAGATC
GATCGAACACCGTACTGACTTGCTACAACAGCCACGTCGACTCCGGAAAGTCGACCAC
CGTGAGTTACACCCTCTTCTCCTGCTCCGATATCAAACGTCGTTTGATACGGGACATCT
ACTCTTTGAACACAGGGCTAACCATTTATCATACAGACCGGTCACTTGATCTACCAGTG
CGGTGGTATCGACCGTCGTACCATCGAGAAGTTCGAGAAGGTAAGCTTCAACTCATTTT
CGCCTCGATTCTCCCTCCACATTCAATTGTGCCCGACAATTCTGCAGAGAATTTTCGCG
TCGACAATTTTTCATCACCCCGCTTTGCATTACCCCTCCTTTGCAGCGACGCAAAATTTT
TTTTGCTGTCGTTTGGTTTTAGTGGGGTTTCTTGTGCACCCCACTAGCTCACTACTTTTT
TTCTGCTTCGCTCTCACTTCCCAGCCATCATTCAACGTGCTCTGTGTCATCACTTTCAGC
GATGCTGACCACTTTTCCATCAATAGGAAGCCGCCGAACTCGGCAAGGGTTCCTTCAA
GTATGCTTGGGTTCTTGACAAGCTCAAGGCCGAGCGTGAGCGTGGTATCACCATCGAC
ATTGCTCTGTGGAAGTTCGAGACTCCCAAGTACTATGTCACCGTCATTGGTATGTTCTTT
CCATCAATTTCACACAGCGATTACAAGCCAGTGCTAACAAGCAATTCACAGACGCTCC
CGGCCACCGTGATTTCATCAAGAACAT

>MK644113.1 Trichoderma rifaii J2-2CR
CCATCGAGAAGTTCGAGAAGGTAAGCTTCAACTCATTTTCACCTCGATTCTCCCTCCAC
ATTCAATTGTGCCCGACAATTCTGCAGAGAATTTTCGCGTCGACAATTTTTCATCACCC
CGCTTTGCATTACCCCTCCTTTGCAGCGACGCAAATTTTTTTGGCTGTCGTTTGGTTTTA

GTGGGGTTTCTTGTGCACCCCACTAGCTCACTACTTTTTTTCTGCTTCGCTCTCATTTCC
CAGCCATCATTCAACGTGCTCTGTGTCATCACTTTCAGCGATGCTAACCACTTTTCCATC
AATAGGAAGCCGCCGAACTCGGCAAGGGTTCCTTCAAGTACGCTTGGGTTCTTGACAA
GCTCAAGGCCGAGCGTGAGCGTGGTATCACCATCGACATTGCTCTGTGGAAGTTCGAG
ACTCCCAAGTACTATGTCACCGTCATTGGTATGTTCTTTCCATCAACTTCACACAGCGAT
TACAAGCCAGTGCTAACAAGCAATTCACAGACGCTCCCGGCCACCGTGATTTCATCAA
GAACAT

>OQ454454.1 Trichoderma rifaii Th14
GAGAAGTTCGAGAAGGTAAGCTTCAACTCATTTTCGCCTCGATTCTCCCTCCACATTCA
ATTGTGCCCGACAATTCTGCAGAGAATTTTCGCGTCGACAATTTTTCATCACCCCGCTT
TGCATTACCCCTCCTTTGCAGCGACGCAAAATTTTTTTTGCTGTCGTTTGGTTTTAGTGG
GGTTTCTTGTGCACCCCACTAGCTCACTACTTTTTTTCTGCTTCGCTCTCACTTCCCAGC
CATCATTCAACGTGCTCTGTGTCATCACTTTCAGCGATGCTGACCACTTTTCCATCAATA
GGAAGCCGCCGAACTCGGCAAGGGTTCCTTCAAGTATGCTTGGGTTCTTGACAAGCTC
AAGGCCGAGCGTGAGCGTGGTATCACCATCGACATTGCTCTGTGGAAGTTCGAGACTC
CCAAGTACTATGTCACCGTCATTGGTATGTTCTTTCCATCAATTTCACACAGCGATTACA
AGCCAGTGCTAACAAGCAATTCACAGACGCTCCCGGCCACCGTGATTTCATCAAGAAC
AT

>FJ463324.1 Trichoderma rifaii DIS 355B
GAGAAGGTAAGCTTCAACTCATTTTCGCCTCGATTCTCCCTCCACATTCAATTGTGCCC
GACAATTCTGCAGAGAATTTTCGCGTCGACAATTTTTCATCACCCCGCTTTGCATTACC
CCTCCTTTGCAGCGACGCAAATTTTTTTTGCTGTCGTTTGGTTTTAGTGGGGTTTCTTGT
GCACCCCACTAGCTCACTACTTTTTTTCTGCTTCGCTCTCACTTCCCAGCCACCATTCA
ACGTGCTCTGTGTCATCACTTTCAGCGATGCTAACCACTTTTCCATCAATAGGAAGCCG
CCGAACTCGGCAAGGGTTCCTTCAAGTACGCTTGGGTTCTTGACAAGCTCAAGGCCGA
GCGTGAGCGTGGTATCACCATCGACATTGCTCTGTGGAAGTTCGAGACTCCCAAGTAC
TATGTCACCGTCATTGGTATGTTCTTTCCATCAAGTTCACACAGCGATTACAAGCCGGT
GCTAACAAGCAATTCACAGACGCTCCCGGCCACCGTGATTTCATCAAGAACAT

>FJ463321.1 Trichoderma rifaii Dis 337F
GAGAAGGTAAGCTTCAACTCATTTTCGCCTCGATTCTCCCTCCACATTCAATTGTGCCC
GACAATTCTGCAGAGAATTTTCGCGTCGACAATTTTTCATCACCCCGCTTTGCATTACC
CCTCCTTTGCAGCGACGCAAATTTTTTTTGCTGTCGTTTGGTTTTAGTGGGGTTTCTT
GTGCACCCCACTAGCTCACTACTTGTTTTCTGCTTCGCTCTCACTTCCCAGCCATCATTC
AACGTGCTCTGTGTCATCACCGATGCTAACCACCTTTCCATCAATAGGAAGCCGCCGAA
CTCGGCAAGGGTTCCTTCAAGTACGCTTGGGTTCTTGACAAGCTCAAGGCCGAGCGTG
AGCGTGGTATCACCATCGACATTGCTCTGTGGAAGTTCGAGACTCCCAAGTACTATGTC
ACCGTCATTGGTATGTTCTTTCCATCAATTTCACACAGCGATTACAAGCCAGTGCTAAC
AAGCGATTCACAGACGCTCCCGGCCACCGTGATTTCATCAAGAACAT

>ON364343.1 Trichoderma simmonsii JKB000479
ACGTGGTCGTTATCGTACGTATTATCCCTTCTTCACGTCGACATCAGTCGCCGCTCTGAT

TCTCAAACACTTGTGCTAACCACCATCTTCTAGGGGTGCGTATTCCATCAATCATCTTGA
GTGAGATCGATCGAACACAGTACTGACTTGTTACAACAGCCACGTCGACTCCGGAAAG
TCGACCACCGTGAGTTGCACCCTCTTATCCTGCTTCGATATCAAACGTCGTTTGATACG
GGACATCTACTCTTTGAACACAGGGCTAACAATTTATCATACAGACCGGTCACTTGATC
TACCAGTGCGGTGGTATCGACCGTCGTACCATCGAGAAGTTCGAGAAGGTAAGCTTCA
ACTCATTTTCGCCTCGATTCTCCCTCCACATTCAATTGTGTCCGACAATTCTGCAGAGA
ATTTTCGTGTCAACAATTTTTCATCACCCCGCTTTGCATTACCCCTCCTTTGCAGCGACG
CAAAAATTTTTTGCTGTCGTTTGGTTTTAGTGGGGTTTCTTGTGCACCCCACTAGCTCG
TTTTTTCTGCTTCGCTCTCACTTCCCAGCCATCATTCAACGTATTCTGTGTCTCGTCACT
TTCAGCGATGCTAACCACTTTTCCATCAATAGGAAGCCGCCGAACTCGGCAAGGGTTC
CTTCAAGTACGCTTGGGTTCTTGACAAGCTCAAGGCCGAGCGTGAGCGTGGTATCACC
ATCGACATTGCTCTGTGGAAGTTCGAGACTCCCAAGTACTATGTCACCGTCATTGGTAT
GTTCTTTCCATCAATTTCACACAGCGATTACAAGCCAGTGCTAACAAGCAATTCACAGA
CGCTCCCGGCCACCGTGATTTCATCAAGAACAT

>MW803053.1 Trichoderma simmonsii T137
GAGAAGGTAAGCTTCAACTCATTTTCGCCTCGATTCTCCCTCCACATTCAATTGTGTCC
GACAATTCTGCAGAGAATTTTCGTGTCAACAATTTTTCATCACCCCGCTTTGCATTACCC
CTCCTTTGCAGCGACGCAAAAATTTTTTGCTGTCGTTTGGTTTTAAGTGGGGTTTCTTG
TGCACCCCACTAGCTCGTTTTTTCTGCTTCGCTCTCACTTCCCAGCCATCATTCAACGTA
TTCTGTGTCTCGTCACTTTCAGCGATGCTAACCACTTTTCCATCAATAGGAAGCCGCCG
AACTCGGCAAGGGTTCCTTCAAGTACGCTTGGGTTCTTGACAAGCTCAAGGCCGAGCG
TGAGCGTGGTATCACCATCGACATTGCTCTGTGGAAGTTCGAGACTCCCAAGTACTATG
TCACCGTCATTGGTATGTTCTTTCCATCAATTTCACACAGCGATTACAAGCCAGTGCTAA
CAAGCAATTCACAGACGCTCCCGGCCACCGTGATTTCATCAAGAACAT

>EF392749.1 Hypocrea lixii DAOM 233986
GTCGTTATCGTACGTATCATCCTTTCTTCATGTCGGCATCATTCGCCGCTCTGATTCTCAA
ACACTTGTGCTAACCACCATCTTCTAGGGGTGCGTATTCCATCAATCATCTTGAATGAG
ATCGATCGAACACAGTACTGACTTGCTACAACAGCCACGTCGACTCCGGAAAGTCGAC
CACCGTGAGTTGCACCCTCTTCTCCTGCTCCGATATCAAACGTCGTTTGATACGGGACA
TCTACTCTTTGAACACAGGGCTAATCATTTATCATACAGACCGGTCACTTGATCTACCAG
TGCGGTGGTATCGACCGTCGTACCATCGAGAAGTTCGAGAAGGTAAGCTTCAACTCAT
TTTCGCCTCGATTCTCCCTCCACATTTAATTGTGCCCGATAATTCTGCAGAGAATTTTCG
TGTCGACAATTTTTCATCACCCCGATTTGCATTACCCCTCCTTTGCAGCGACGCAAATTT
TTTTGGCTGTCGTTTGGTTTTAGTGGGGTTTCTCGTGCACCCCACTAGGTCACTGCTTT
TTTTCTGCTTCGCTCTTACTGCCCAGCCATCATTCAACGTGCTCTGCGTCTCATCACTTT
CAGCGATGCTAACCACTTTTCCATCAATAGGAAGCCGCCGAACTCGGCAAGGGTTCCT
TCAAGTACGCTTGGGTTCTTGACAAGCTCAAGGCCGAGCGTGAGCGTGGTATCACCAT
CGACATTGCTCTGTGGAAGTTCGAGACTCCCAAGTACTATGTCACCGTCATTGGTATGT
TCTTTCCATCAACTTCACACAGCGATTACAAGCCAGTGCTAACAAGCAATTCACAGAC
GCTCCCGGCCACCGTGATTTCATCAAGAACAT

>OQ362346.1 Trichoderma afroharzianum BThr37

ACGTGGTCGTTATCGTACGTACAATCCTGTTCCCTCACGTCGGCATCATTCGCCGGTCT
GATTCTCAAACACTTGTGCTAACCATTGCCTTCTAGGGGTGCGTATTCCATCAATCATCT
TGAATGAGATCGATCGAACACAATACTGACTTGCTATAACAGCCACGTCGACTCCGGA
AAGTCGACCACCGTAAGTTGCGCCCTCTCTTGCTCCGATATCAAACGTCGTTTGATGCG
GGACACCTATTCTTGAACACAGGGCTAACCATGCATAATACAGACCGGTCACTTGATCT
ACCAGTGCGGTGGTATCGACCGTCGTACCATCGAGAAGTTCGAGAAGGTAACCTTCAA
CTGATTTTCGCCTCGATTCTTCCTCTCTTCACATTCAATTGTGCCCGACAATTCTGCAGA
GAATTTTCGTGTCGACAATTTTTCATCACCCCGCTTTCCATTACCCCTCCTTTCCAGCGA
CGCAAATTTTTTTTTCTGTCGTTTGGTTTTTAGTGGGGTTCTCTGTGCAACCCCACTAGC
TCCCTGCTTTTTCCTGCTTCACTCTCACTTCCTCGTCATCATTCAACGTGCTCTGCGTCT
TTGGTCATTCAGCGACGCTAACCACTTTTCCATCAATAGGAAGCCGCCGAACTCGGCA
AGGGTTCCTTCAAGTACGCTTGGGTTCTTGACAAGCTCAAGGCCGAGCGTGAGCGTGG
TATCACCATTGACATTGCTCTGTGGAAGTTCGAGACTCCCAAGTACTATGTCACCGTCA
TTGGTAAGTCTTCACTAAGTTCATGCTGCAATTGCGGACCAGTGCTAACAGGCAATTCA
CAGACGCTCCCGGCCACCGTGATTTCATCAAGAACAT

>OR146260.1 Trichoderma afroharzianum T22
ACGTACAATCCTGTTCCCTCACGTCGGCATCATTCGCCGGTCTGATTCTCAAACACTTG
TGCTAACCATTGCCTTCTAGGGGTGCGTATTCCATCAATCATCTTGAATGAGATCGATCG
AACACAATACTGACTTGCTATAACAGCCACGTCGACTCCGGAAAGTCGACCACCGTAA
GTTGCGCCCTCTCTTGCTCCGATATCAAACGTCGTTTGATGCGGGACACCTATTCTTGA
ACACAGGGCTAACCATGCATAATACAGACCGGTCACTTGATCTACCAGTGCGGTGGTAT
CGACCGTCGTACCATCGAGAAGTTCGAGAAGGTAACCTTCAACTGATTTTCGCCTCGA
TTCTTCCTCTCTTCACATTCAATTGTGCCCGACAATTCTGCAGAGAATTTTCGTGTCGAC
AATTTTTCATCACCCCGCTTTCCATTACCCCTCCTTTCCAGCGACGCAAATTTTTTTTTC
TGTCGTTTGGTTTTTAGTGGGGTTCTCTGTGCAACCCCACTAGCTCCCTGCTTTTTCCTG
CTTCACTCTCACTTCCTCGTCATCATTCAACGTGCTCTGCGTCTTTGGTCATTCAGCGAC
GCTAACCACTTTTCCATCAATAGGAAGCCGCCGAACTCGGCAAGGGTTCCTTCAAGTA
CGCTTGGGTTCTTGACAAGCTCAAGGCCGAGCGTGAGCGTGGTATCACCATTGACATT
GCTCTGTGGAAGTTCGAGACTCCCAAGTACTATGTCACCGTCATTGGTAAGTCTTCACT
AAGTTCATGCTGCAATTGCGGACCAGTGCTAACAGGCAATTCACAGACGCTCCCGGCC
ACCGTGATTTCATCAAGAACAT

>MH395412.1 Trichoderma afroharzianum GJS 08-156
AAGGTAAGCTTCAACTGATTTTCGCCTCGATTCTTCCTCCTCCACATTCAATTGTGCCCG
ACAATTCTGCAGAGAATTTTCGTGTCGACAATTTTTCATCACCCCGCTTTCCATTACCCC
TCCTTTGCAGCGACGCAAATTTTTTTTGCTGTCGTTTGGTTTTTAGTGGGGTTCTCTGTG
CAACCCCACTAGCTCCCTGCTTTTTCCTGCTTCACTCTCACTTCCTCGTCATCATTCAAC
GTGCTCTGCGTCTTTGGTCATTCAGCGACGCTAACCACTTTTCCATCAATAGGAAGCCG
CCGAACTCGGCAAGGGTTCCTTCAAGTACGCTTGGGTTCTTGACAAGCTCAAGGCCGA
GCGTGAGCGTGGTATCACCATTGACATTGCTCTGTGGAAGTTCGAGACTCCCAAGTAC
TATGTCACCGTCATTGGTAAGTCTTCACTAAGTTCATGCTGCAATTGCGGACCAGTGCT
AACAGGCAATTCACAGACGCTCCCGGCCACCGTGATTTCATCAAGAACAT

>FJ463302.1 Trichoderma afroharzianum GJS 04-197
GAGAAGGTAAGCTTCAACTGATTTTCGCCTCGATTCTTCCTCCTCCACATTCAATTGTG
CCCGACAATTCTGCAGAGAATTTTCGTGTCGACAATTTTTCATCACCCCGCTTTCCATTA
CCCCTCCTTTGCAGCGACGCAAATTTTTTTTGCTGTCGTTTGGTTTTTAGTGGGGTTCTC
TGTGCAACCCCACTAGCTCCCTGCTTTTTCCTGCTTCACTCTCACTTCCTCGTCATCATT
CAACGTGCTCTGCGTCTTTGGTCATTCAGCGACGCTAACCACTTTTACATCAATAGGAA
GCCGCCGAACTCGGCAAGGGTTCCTTCAAGTACGCTTGGGTTCTTGACAAGCTCAAGG
CCGAGCGTGAGCGTGGTATCACCATTGACATTGCTCTGTGGAAGTTCGAGACTCCCAA
GTACTATGTCACCGTCATTGGTAAGTCTTCACTAAGTTCATGCTGCAATTGCGGACCAG
TGCTAACAGGCAATTCACAGACGCTCCCGGCCACCGTGATTTCATCAAGAACAT

>FJ463301.1 Trichoderma afroharzianum GJS 04-186
GAGAAGGTAAGCTTCAACTGATTTTCGCCTCGATTCTTCCTCCTCCACATTCAATTGTG
CCCGACAATTCTGCAGAGAATTTTCGTGTCGACAATTTTTCATCACCCCGCTTTCCATTA
CCCCTCCTTTGCAGCGACGCAAATTTTTTTTGCTGTCGTTTGGTTTTTAGTGGGGTTCTC
TGTGCAACCCCACTAGCTCCCTGCTTTTTCCTGCTTCACTCTCACTTCCTCGTCATCATT
CAACGTGCTCTGCGTCTTTGGTCATTCAGCGACGCTAACCACTTTTACATCAATAGGAA
GCCGCCGAACTCGGCAAGGGTTCCTTCAAGTACGCTTGGGTTCTTGACAAGCTCAAGG
CCGAGCGTGAGCGTGGTATCACCATTGACATTGCTCTGTGGAAGTTCGAGACTCCCAA
GTACTATGTCACCGTCATTGGTAAGTCTTCACTAAGTTCATGCTGCAATTGCGGACCAG
TGCTAACAGGCAATTCACAGACGCTCCCGGCCACCGTGATTTCATCAAGAACAT
