# Supplementary material for: Genomic Characterization and Establishment of a Genetic Manipulation System for Trichoderma sp. (Harzianum Clade) LZ117
Source: J Fungi (Basel). 2024 Oct 7;10(10):697. doi: 10.3390/jof10100697 (PMC11508783; doi:10.3390/jof10100697)
Supplement: Supplementary file 1 [file jof-10-00697-s001.zip › Supplementary Materials S3.pdf]

Table S1 Primers used in this work

Primers	Sequences
RPB2-5f	GACGACAGTGATCACTTCGG
RPB2-7cr	CCCATAGCTTGCTTACCCAT
KO-MaltoP-up-F	AAGATGGATGGATAGGCTACTTTGA
KO-MaltoP-up-R	GTTCGGACCGATTCTGTGATTG
KO-MaltoP-dn-F	AAAAGGCAGCGCACAATATCAA
KO-MaltoP-dn-R	GCTATCTTTCCTCATGTTCGGTTG
Hph-MaltoP-F	ACCAATCACAGAATCGGTCCGAACATGCCAGAACTCACCGCGAC
Hph-MaltoP-R	TATTGATATTGTGCGCTGCCTTTTCTATTCCTTTGCCCTCGGTCG
MaltoP-orfcheck-F	TGGCTGGCATCGTTATCGTCAC
MaltoP-orfcheck-R	TACCACCCAATCCGCAGGCACT
MaltoP-UAcheck-F	GGAGTCTCGGTGGTGAGTTGAA
MaltoP-UAcheck-R	GATGTTGGCGACCTCGTATTGG
EO-MaltoP-F	aaccgcggactgcgcatcccatggATGGGCTCTTCAGACGATGAGA
EO-MaltoP-R	GTAACGTTAAGTGTATTCTCTAGA TTAGTAAGAAGACAGTTGGTAGCTG
LZ117-OECre1-F	aaccgcggactgcgcatcccatggATGCAGCGAGCTCAGTCTGC
LZ117-OECre1-R	GTAACGTTAAGTGTATTCTCTAGATTAGACGGGCCGCCAGAGA
PgpdA-F	AAGCTTGCACTATTGATCATCCG
TtrpC-R	GAGTGGAGATGTGGAGTGGGC
Actin-qPCR-F	TCGCTCTTCCTCACGCCATTG

Actin-qPCR-R	CCACGCTCAGCCAGGATCTTC
Cre1-qPCR-F	CAGCACCAGCAGCACCTTCAC
Cre1-qPCR-R	AGGCGAGACGTTAGGCGAGAC

Table S2 CAZymes harbored by selected *Trichoderma* species.

	GH	GT	PL	CE	AA	CBM
Trichoderma sp. LZ117	504	237	12	62	111	136
T. atrobrunneum ITEM 908[a]	262	87	6	69	72	40
T. harzianum B97[a]	266	89	7	69	76	40
T. harzianum T6776 [a]	259	88	7	70	74	52
T. atroviride IMI206040[a]	210	82	7	17	34	77
T. atroviride JMC9410[a]	246	87	9	69	74	63
T. reesei QM6a[a]	199	92	5	16	32	55
T. virens Gv-29-8[a]	219	79	4	20	39	95

[a]Data from Fanelli F, Liuzzi VC, et al., 2018[11].

GH = glycoside hydrolases; GT = glycoside transferases; PL = polysaccharide lyases; CE = carbohydrate esterases; AA = auxiliary activities; CBM = carbohydrate-binding modules.

Table S3 Potential biosynthetic gene clusters in LZ117 showing more than 100% similarity with the known clusters predicted by antiSMASH

Contig	From	To	Type	Most similar known cluster
1	780,945	807,889	RiPP	Informatipeptin
1	2,199,770	2,221,941	Terpene	Geosmin
1	1,081,477	1,102,592	Terpene	Geosmin
1	3,802,833	3,882,383	T1PKS	Macbecin
1	5,416,932	5,521,136	NRPS, T1PKS	Nocardicin
1	13,584	33,619	RiPP	SapB
2	89,387	111,540	Terpene	Geosmin
2	310,491	363,256	Terpene	2-methylisoborneol
2	1,909,868	1,981,053	NRPS	Himastatin
2	5,487,262	5,508,657	Terpene	Geosmin
2	9,591,063	9,610,695	Terpene	Pristinol
2	1,793,929	1,817,420	RiPP	Planosporicin
2	8,525,562	8,643,216	T1PKS	Natamycin
2	8,787,397	8,807,068	RiPP	SapB
2	2,929,660	2,949,713	Terpene	Geosmin
2	4,424,071	4,539,325	T1PKS	Heronamide
2	5,939,841	6,065,465	T1PKS, NRPS	Piericidin A1
2	959,179	1,008,428	NRPS	Coelichelin
2	1,775,843	1,947,936	T1PKS	Nigericin
2	4,289,828	4,330,154	NRPS-like	Echoside
2	5,951,915	5,972,113	Terpene	Geosmin
2	9,576,680	9,596,529	Terpene	Pristinol
2	11,417,357	11,485,648	NRPS, betalactone	Coelichelin
2	523,957	572,095	T1PKS, NRPS	SGH PTMs
2	2,878,270	2,900,451	RiPP	AmfS
3	1,181,380	1,203,859	RiPP	Burhizin
3	1,441,662	1,490,637	NRPS	Rhizomide
3	7,191,342	7,213,981	RiPP	SapB
3	1,932,149	1,989,507	Terpene, NRPS-like	2-methylisoborneol
3	2,292,821	2,354,301	NRPS	Albachelin

3	362,399	421,882	Terpene, NRPS, T1PKS	Antimycin
3	5,128,147	5,147,586	Terpene	Albaflavenone
3	6,972,987	7,040,828	NRPS	Netropsin
4	1,115,235	1,131,575	Terpene	Carotenoid
4	5,467,026	5,642,413	NRPS, T1PKS	Phenalamide A2
4	3,038,616	3,059,139	Terpene	Albaflavenone
4	8,450,351	8,477,657	RiPP	Informatipeptin
5	1,665,792	1,809,065	T1PKS	E-837
5	6,706,375	6,747,305	NRPS-like	Chloramphenicol
5	1,898,267	1,917,916	Terpene	Carotenoid
5	3,934,152	4,019,182	NRPS, T1PKS	Crocacin
5	6,373,638	6,446,022	NRPS, T1PKS	Chondramide A
5	7,162,758	7,435,047	T1PKS, NRPS-like, NRPS	Chondrochloren A
5	429,123	452,684	Terpene	Carotenoid
5	2,412,531	2,470,165	NRPS	Rhodochelin
6	2,390,969	2,410,569	Terpene	2-methylisoborneol
6	8,111,662	8,130,919	Terpene	Geosmin
6	10,469,328	10,490,317	Terpene	Geosmin
6	282,039	304,324	Terpene	Geosmin
6	9,281,860	9,353,915	NRPS, T1PKS	Melithiazol A
6	543,837	566,449	RiPP	Citrulassin D
6	587,990	613,584	Terpene	Isorenieratene
6	5,158,533	5,179,192	Terpene	Geosmin
7	942,106	1,083,781	T1PKS	Nystatin-like Pseudonocardia polyene
7	561,630	609,760	T1PKS, NRPS	SGH PTMs
7	2,913,278	2,935,460	RiPP	AmfS
7	6,555,445	6,843,909	T1PKS, NRPS-like, NRPS	Candicidin
7	136,821	196,004	NRPS	Coelichelin
7	10,469,328	10,490,317	RiPP	Informatipeptin
7	25,590	46,275	Terpene	Geosmin
7	475,946	529,209	RiPP	Citrulassin D

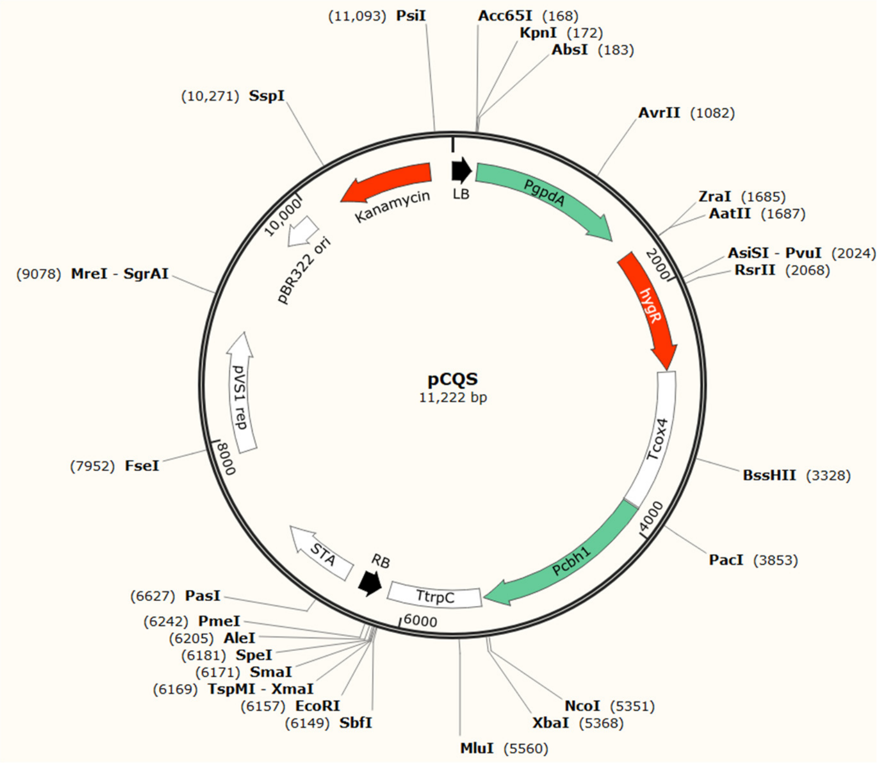

Figure S1. Plasmid pCQS used for the overexpression of targeted genes

Strain ID	Most closely related taxon		Similarity % to Reference Sequences		Molecular identification				
	Species	Reference strain	*rpb2*	*tef1*	Species	Infrageneric group	Precision	Accuracy	Ambiguity
LZ117	*T. atrobrunneum*	ITEM 908	98.15	96.66	*T.* sp. aff. *atrobrunneum*	*Harzianum* Clade	species	accurate	Unambiguous

Figure S2. Molecular identification results. The sequence similarity threshold sufficient for species identification is set as ≥99% for *rpb2* and ≥97% for *tef1* DNA Barcode loci (Cai and Druzhinina, 2021). To achieve high identification precision, sequences were trimmed to the length of diagnostic fragments using *Tricho*MARK (www.trichokey.com).

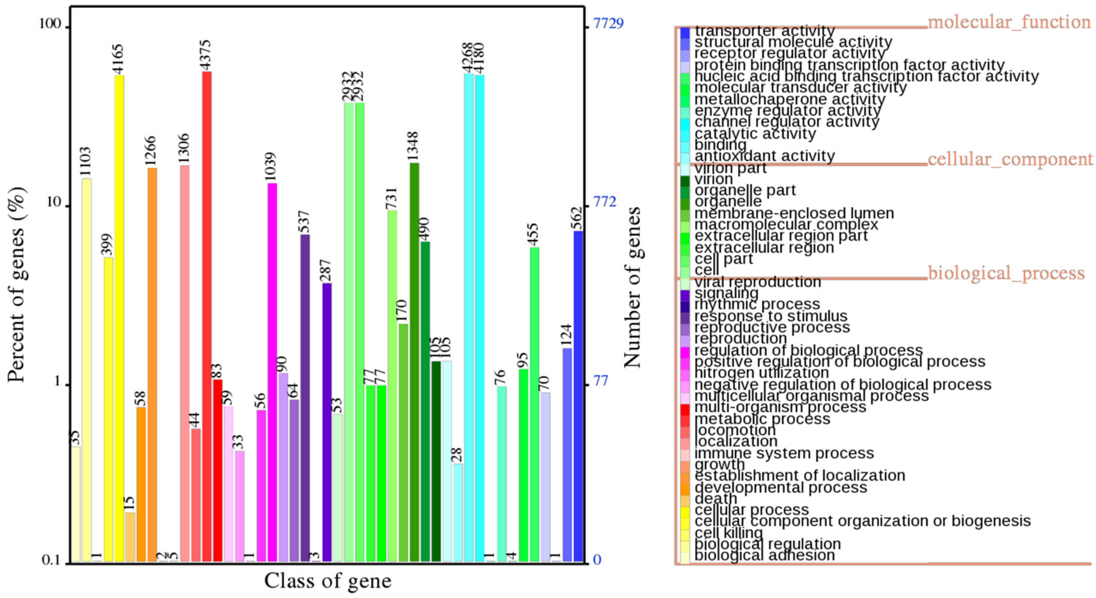

Figure S3. GO functional annotation of *Trichoderma* sp. LZ117. GO function classification were assigned to three major groups including molecular (I), cellular component (II), and biological process (III). The y axis points out the name of the most enriched GOs that belong to upon three different ontologies; while the x axis denotes the number of LZ117 genes.

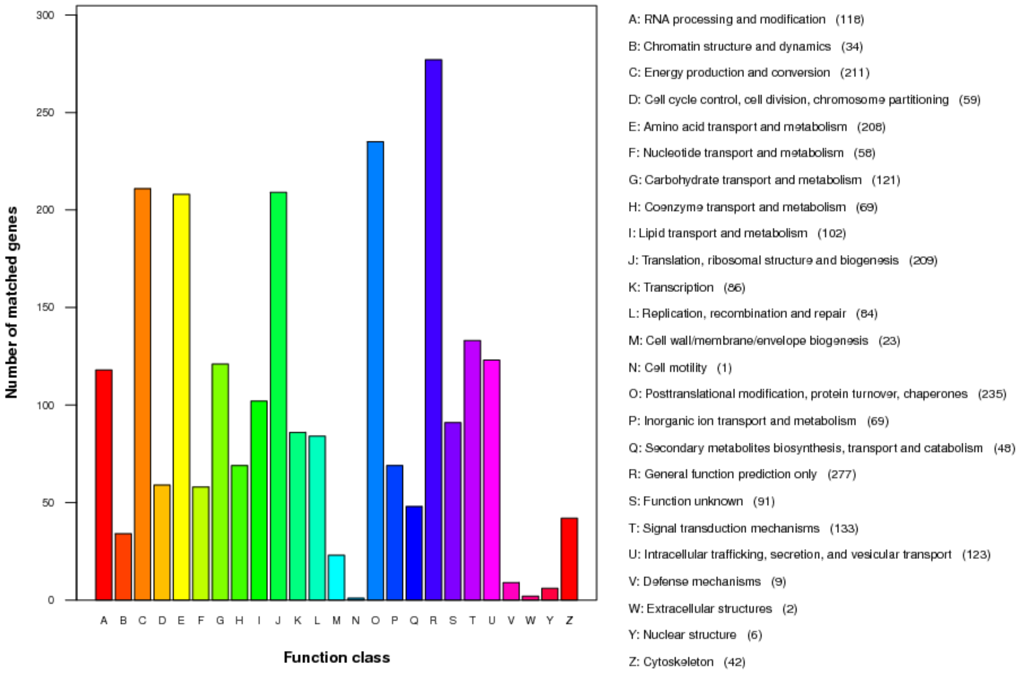

Figure S4. KOG functional classification of *Trichoderma* sp. LZ117 gene. The y axis points out the number of the matched genes, and the x axis denotes the function class.

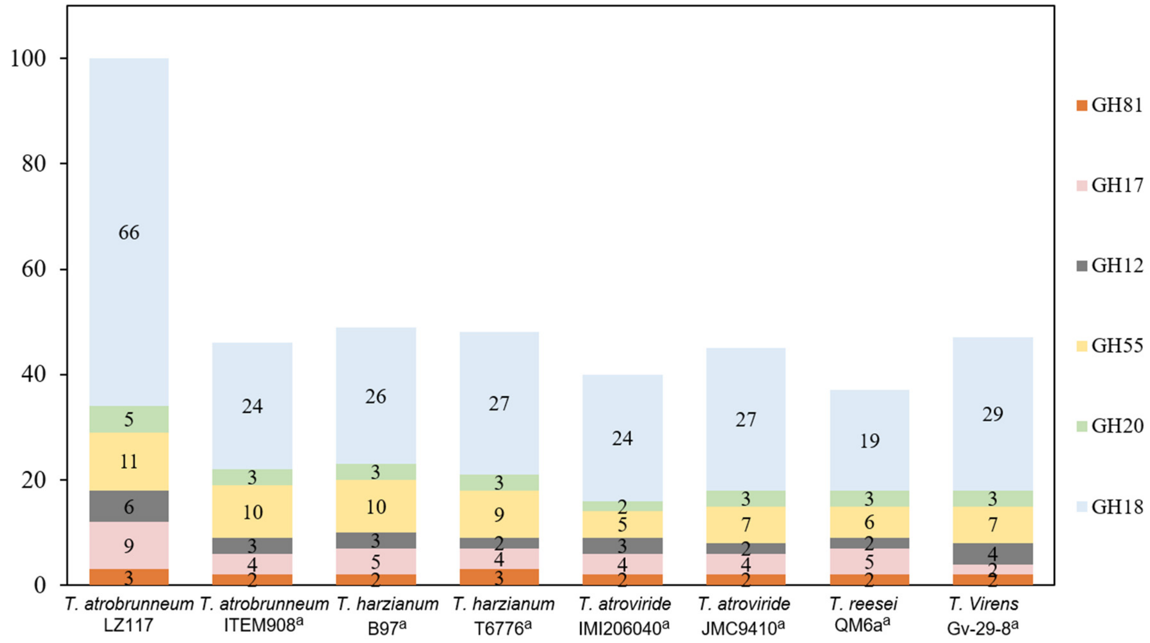

Figure S5. Glycoside hydrolases related to mycoparasitic activity in *Trichoderma* sp. LZ117. [a]Data from Fanelli F, Liuzzi VC, et al., 2018[11]. GH = glycoside hydrolases; chitinases: GH18 and GH20; glucanases: GH55, GH12, GH17 and GH81; cellulases: GH6 and GH7

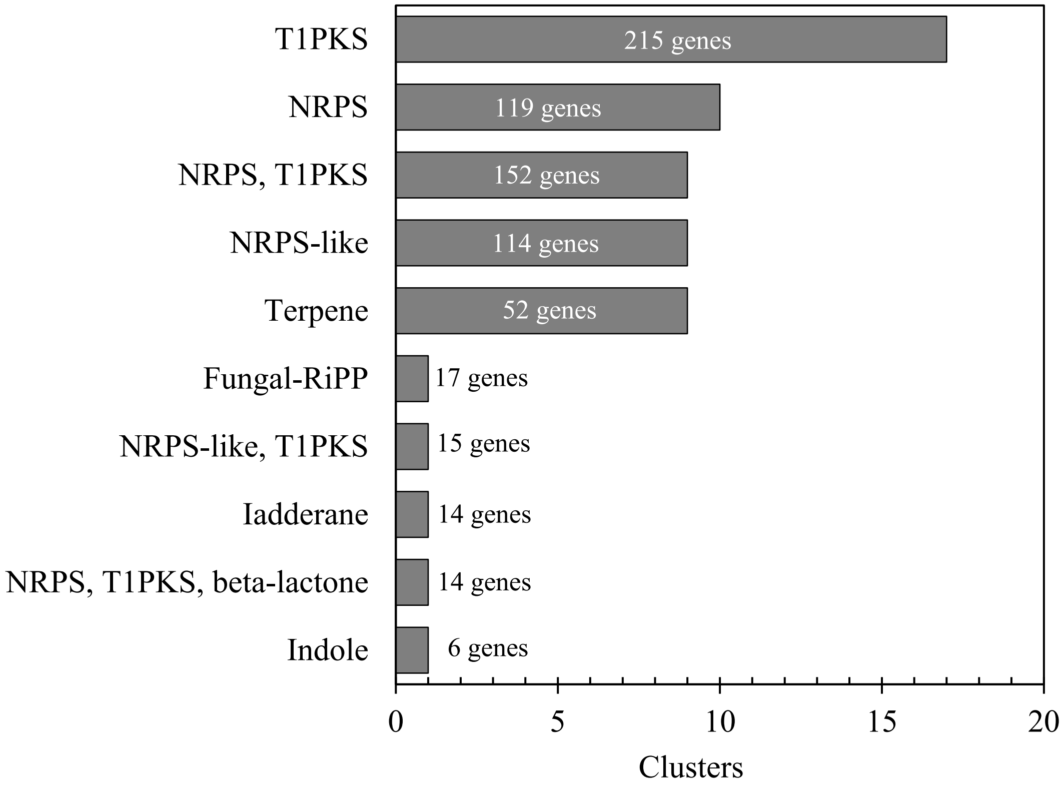

Figure S6. The clusters involved in secondary metabolite synthesis. T1PKS: type I polyketide synthase; NRPS: nonribosomal peptide synthetase; Fungal-RiPP: fungal RiPP with POP or UstH peptidase types and a modification.

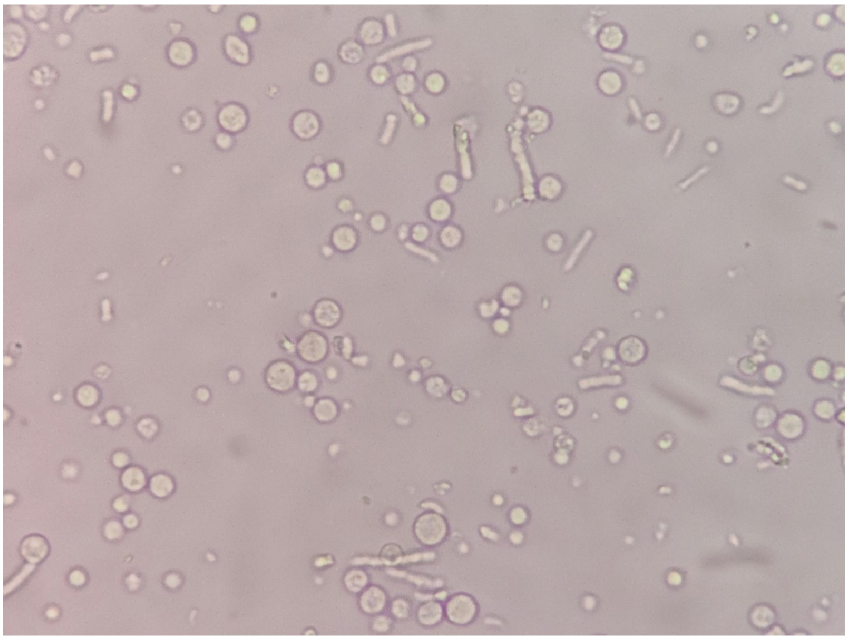

Figure S7. Microscopy of the lysed protoplasts of *Trichoderma* sp. LZ117

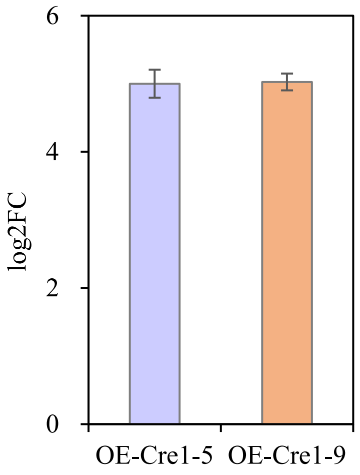

Figure S8. Transcriptional analysis by quantitative reverse transcription polymerase chain reaction for *cre1*. Error bars show the standard deviations. The expression of genes was normalized to that for the reference gene *act* encoding for actin.
