# Supplementary material for: Genomic Characterization and Establishment of a Genetic Manipulation System for Trichoderma sp. (Harzianum Clade) LZ117
Source: J Fungi (Basel). 2024 Oct 7;10(10):697. doi: 10.3390/jof10100697 (PMC11508783; doi:10.3390/jof10100697)
Supplement: Supplementary file 1 [file jof-10-00697-s001.zip › Supplementary Materials S7.pdf]

>*Trichoderma* sp. LZ117
MQRAQSAVDFSNLLNPTSAAGQDSDAEQGSGAMSTAAVTVIKPNGPIPGAQSTETANELP
RPYKCPLCEKAFHRLEHQTRHIRTHTGEKPHACQFPGCSKKFSRSDELTRHSRIHSNPNSR
RGNKGQQQHQQHLHHQGLPHHMHVDGMMPPPVPKAIRSAPTSTLVSPNVSPPHSYSSFV
MPQTPHGSLQPWQRYHNAGKGCKPDRAGNSLWRPV

>*Aspergillus mulundensis* (XP_026607409.1)
MPQSVSSVDFSNLLNPQNTAIPAEVSNTTASATMATGASLLPPMMKGARPAAEEARQDLP
RPYKCPLCERAFHRLEHQTRHIRTHTGEKPHACQFPGCSKRFSRSDELTRHSRIHNNPNSR
RGNKAQHLAAAAAAAAASQDGSPLANNAGSMMPPPSKPITRSAPVSQVGSPDISPPHSFS
NFANHMRPNLSPYSR

>*Aspergillus nidulans* FGSC A4 (XP_663799.1)
MPQPGSSVDFSNLLNPQNNTAIPAEVSNATASATMASGASLLPPMVKGARPAAEEARQDL
PRPYKCPLCERAFHRLEHQTRHIRTHTGEKPHACQFPGCSKRFSRSDELTRHSRIHNNPNSR
RGNKAQHLAAAAAAAAANQDGSAMANNAGSMMPPPSKPITRSAPVSQVGSPDISPPHSF
SNYA

>*Aspergillus versicolor* CBS 583.65 (XP_040667254.1)
MPQQASSVDFSNLLNPQNNTSTTVDSSNSPPTQPSTQSTMATGTSLLPPMMKGGRPAAEE
PRQDLPRPYKCPLCERAFHRLEHQTRHIRTHTGEKPHACQFPGCSKRFSRSDELTRHSRIH
NNPNSRRGNKAQHLAAAAAAAAATHDGTGMPNNAGSMMPPPSKPITRSAPVSQVGSPD
VSPPHSFSNYAQHMRSNLSPYSRNND

>*Fusarium xylarioides* (KAG5768173.1)
MQRAQSAVDFSNLLNPTVPADKESEKPHQGDVEMATAAVTVIKPNGPLPGVQNSENSNEL
PRPYKCPLCDKAFHRLEHQTRHIRTHTGEKPHACQFPGCSKKFSRSDELTRHSRIHNNPNS
RRGNKAAQAHQQQGLPPHMMPDGMMAPPPAPKTIRSAPGSALASPNVSPPHSYSTFALP

>*Fusarium decemcellulare* (KAF5007444.1)
MQRAQTAVDFSNLLNPTAAADKEPEKPQQGDVEMATAAVTVIKPNGPLPGAQASEASNEL
PRPYKCPLCDKAFHRLEHQTRHIRTHTGEKPHACQFPGCSKKFSRSDELTRHSRIHNNPNS
RRGNKAAQQHQQQQQHQMHQQQGLPPHMHPDGMMAPPPAPKTIRSAPTSTLASPNVSP
PHSYSTFALP

>*Fusarium oxysporum* (SCO85024.1)
MQRAQSAVDFSNLLNPTVPADKESEKPQQGDVEMATAAVTVIKPNGPLPGVQNSENSNEL
PRPYKCPLCDKAFHRLEHQTRHIRTHTGEKPHACQFPGCSKKFSRSDELTRHSRIHNNPNS
RRGNKAAQAHQQQQHQMHQQQGLPPHMMPDGMMAPPPAPKTIRSAPGSALASPNVSPP
HSYSTFALP

>*Saccharomyces cerevisiae* (CAA39084.1)
MQSPYPMTQVSNVDDGSLLKESKSKSKVAAKSEAPRPHACPICHRAFHRLEHQTRHMRIH
TGEKPHACDFPGCVKRFSRSDELTRHRRIHTNSHPRGKRGRKKKVVGSPINSASSSATSIPD

LNTANFSPPLPQQHLSPLIPIAIAPKENSSRSSTRKGRKTKFEIGESGGNDPYMVSSPKTMA
KIPVSVKPPPSLALNNMNYQTSSASTALSSLSNSHSGSRLKLNALSSLQMMTPIASSAPRTV
FIDGPEQKQLQQQQNSLSPRYSNTVILPRPRSLTDFQGLNNANPNNNGSLRAQTQSSVQLK
RPSSVLSLNDLLVGQRNTNESDSDFTTGGEDEEDGLKDPSNSSIDNLEQDYLQEQSRKKSK
TSTPTTMLSRSTGRVWSP

>*T. asperellum* CBS 433.97 (XP_024765178.1)
MQRARSAVDFSNLLNPSSATPSQDQGSGAMSTAAVTVIKPNGPIPGAQASESANELPRPYK
CPLCDKAFHRLEHQTRHIRTHTGEKPHACQFPGCSKKFSRSDELTRHSRIHSNPNSRRGNK
GQQQHQQHQQHLHQGMPHPLHVDGMMAPPPAPKAIRSAPASALVSPNVSPPHSYSSFAVP

>*T. atrobrunneum* ITEM908
MQRAQSAVDFSNLLNPTSAAGQDSDAEQGSGAMSTAAVTVIKPNGPIPGAQSTETANELP
RPYKCPLCEKAFHRLEHQTRHIRTHTGEKPHACQFPGCSKKFSRSDELTRHSRIHSNPNSR
RGNKGQQQHQQHLHHQGLPHHMHVDGMMPPPVPKAIRSAPTSTLVSPNVSPPHSYSSFV
MPQTPMAHYNRGNDITMLAKAANQIERETLSGGPS

>*T. atroviride* IMI 206040 (XP_013941427.1)
MQRARSAVDFSNLLNPSSAAPSQDQGSGAMSTAAVTVIKPNGPIPGAQASESANELPRPYK
CPLCDKAFHRLEHQTRHIRTHTGEKPHACQFPGCSKKFSRSDELTRHSRIHSNPNSRRGNK
GQQQHQQHLHQGMPHPLHVDGMMAPPPAPKAIRSAPASALVSPNVSPPHSYSSFAVP

>*T. citrinoviride* (XP_024754019.1)
MQRAQSAVDFSNLLNPTSAAGQDSGAMSTAAVTVIKPNGPIPGTQSTETANELPRPYKCPL
CDKAFHRLEHQTRHIRTHTGEKPHACQFPGCSKKFSRSDELTRHSRIHSNPNSRRGNKGQQ
QHQLHHQGMPHPMHVDGLMHPPAAPKAIRSAPPSTLVSPNVSPPHSYSSFVMPHGP

>*T. gamsii* (XP_018659586.1)
MSTAAVTVIKPNGPIPGAQASESANELPRPYKCPLCDKAFHRLEHQTRHIRTHTGEKPHAC
QFPGCSKKFSRSDELTRHSRIHSNPNSRRGNKGQQQHQQHLHQGMPHPLHVDGMMAPPP
APKAIRSAPASALVSPNVSPPHSYSSFAVP

>*T. harzianum* CBS 226.95 (XP_024780493.1)
MQRAQSAVDFSNLLNPTSAAGQDSDAEQGSGAMSTAAVTVIKPNGPIPGAQSTEAANELP
RPYKCPLCEKAFHRLEHQTRHIRTHTGEKPHACQFPGCSKKFSRSDELTRHSRIHSNPNSR
RGNKGQQQHQQHLHHQGLPHHMHVDGMMPPPVPKAIRSAPTSTLVSPNVSPPHSYSSFV
MPQTP

>*T. koningii* (AFD62913.1)
MQRAQSAVDFSNLLNPTSAAGQDSGAMSTAAVTVIKPNGPIPGTQATETANELPRPYKCPL
CDKAFHRLEHQTRHIRTHTGEKPHACQFPGCSKKFSRSDELTRHSRIHSNPNSRRGNKGQQ
QHQLHHQGMPHPMHVDGLMHPPAAPKAIRSAPPSTLVSPNVSPPHSYSSFVMPHGP

>*T. lixii* (CAA64656.1)

MQRAQSAVDFSNLLNPTSAAGQDSDAEQGSGAMSTAAVTVIKPNGPIPGAQSTEAANELP
RPYKCPLCEKAFHRLEHQTRHIRTHTGEKPHACQFPGCSKKFSRSDELTRHSRIHSNPNSR
RGNKGQQQHQQHLHHQGLPHHMHVDGMMPPPVPKAIRSAPTSTLVSPNVSPPHSYSSFV
MPQTP

>*T. orientale* (AFD50191.1)
MQRAQSAVDFSNLLNPTSAAGQDSGAMSTAAVTVIKPNGPIPGTQATETANELPRPYKCPL
CDKAFHRLEHQTRHIRTHTGEKPHACQFPGCSKKFSRSDELTRHSRIHSNPNSRRGNKGQQ
QHQLHHQGMPHPMHVDGLMHPPAAPKAIRSAPPSTLVSPNVSPPHSYSSFVMPHGP

>*T. reesei QM6a* (XP_006962129.1)
MQRAQSAVDFSNLLNPTSAAGQDSGAMSTAAVTVIKPNGPIPGTQSTETANELPRPYKCPL
CDKAFHRLEHQTRHIRTHTGEKPHACQFPGCSKKFSRSDELTRHSRIHSNPNSRRGNKGQQ
QHQLHHQGMPHPMHVDGLMHPPAAPKAIRSAPPSTLVSPNVSPPHSYSSFVMPHGP

>*T. reesei* RUT C-30 (ETS05463.1)
NGTAGQDSGAMSTAAVTVIKPNGPIPGTQSTETANELPRPYKCPLCDKAFHRLEHQTRHIR
THTGEKPHACTSITC
